# Assessing the effects of 24-epibrassinolide and yeast extract at various levels on cowpea’s morphophysiological and biochemical responses under water deficit stress

**DOI:** 10.1186/s12870-023-04548-6

**Published:** 2023-11-27

**Authors:** Faride Gholami, Mohamad Reza Amerian, Hamid Reza Asghari, Amin Ebrahimi

**Affiliations:** Agronomy and Plant Breeding Department, Faculty of Agriculture, Shahrood University of Technology, Semnan, Iran

**Keywords:** Water deficit stress, Yeast extract, 24-epibrassinolide, Cowpea, Yield components, Morphophysiological and biochemical responses

## Abstract

**Background:**

Due to the factor of water deficit, which has placed human food security at risk by causing a 20% annual reduction in agricultural products, addressing this growing peril necessitates the adoption of inventive strategies aimed at enhancing plant tolerance. One such promising approach is employing elicitors such as 24-epibrassinolide (EBR) and yeast extract, which are potent agents capable of triggering robust defense responses in plants. By employing these elicitors, crops can develop enhanced adaptive mechanisms to combat water deficit and improve their ability to withstand drought condition. This study investigates the impact of different levels of EBR (0, 5, 10 µm) and yeast extract (0 and 12 g/l) on enhancing the tolerance of cowpea to water deficit stress over two growing seasons.

**Results:**

The findings of this study demonstrate that, the combined application of EBR (especially 10 µm) and yeast extract (12 g/l) can increase seed yield (18%), 20-pod weight (16%), the number of pods per plant (18%), total chlorophyll content (90%), and decrease malondialdehyde content (45%) in cowpea, compared to plants grown under water deficit stress without these treatments. Upon implementing these treatments, impressive results were obtained, with the highest recorded values observed for the seed yield (1867.55 kg/ha), 20-pod weight (16.29 g), pods number per plant (9), and total chlorophyll content (19.88 mg g^−1^ FW). The correlation analysis indicated a significant relationship between the seed yield, and total chlorophyll (0.74**), carotenoids (0.82**), weight of 20 seeds (0.67**), and number of pods (0.90**). These traits should be prioritized in cowpea breeding programs focusing on water deficit stress.

**Conclusions:**

The comprehensive exploration of the effects of EBR and yeast extract across various levels on cowpea plants facing water deficit stress presents a pivotal contribution to the agricultural domain. This research illuminates a promising trajectory for future agricultural practices and users seeking sustainable solutions to enhance crops tolerance. Overall, the implications drawn from this study contribute significantly towards advancing our understanding of plant responses to water deficit stress while providing actionable recommendations for optimizing crop production under challenging environmental conditions.

**Supplementary Information:**

The online version contains supplementary material available at 10.1186/s12870-023-04548-6.

## Background

Plants are constantly subjected to various environmental hazards, for instance biotic and abiotic stresses, which significantly impede their growth and yield [1, 2]. These environmental stressors can induce morphological, physiological, metabolic, biochemical, and molecular changes in plants, leading to severe growth inhibition and reduced yields [2, 3]. Drought stress poses a substantial environmental challenge that can profoundly impact both the vegetative and reproductive growth of plants [4]. Drought stress can significantly impact essential physiological processes in plants, including respiration, photosynthesis, and transpiration. This destructive stress alters cell turgor and disrupts the normal opening and closing of stomata, which are crucial for gas exchange. As a result, enzymatic reactions that rely on water availability are affected, leading to reduced plant growth [5]. In order to alleviate the detrimental impacts of water stress on plants, multiple strategies are employed, including the application of growth regulators. The growth regulators’ application offers a valuable approach for manipulating plant physiology and facilitating adaptive responses that enhance plants’ ability to cope with water stress conditions. By utilizing these regulators, researchers aim to promote physiological adjustments in plants that improve their tolerance and yield under limited water availability. This innovative technique holds promise for cultivating crops in sustainable agriculture practices, as it contributes to the development of drought-tolerant varieties and enables efficient utilization of water resources [6].

The utilization of growth regulators to improve drought tolerance in plants has been extensively documented. In comparison to plant breeding techniques, which can be time-consuming and complex, the application of growth regulators, such as epibrassinolides (EBRs) [7, 8], offers a relatively straightforward approach. EBRs are factors that stimulate cell division and promote root and stem growth, improving plant growth and mitigating the negative effects of environmental stresses [9]. EBRs have been found to enhance plant tolerance against salinity [10], drought [11], and high-temperature stresses [12, 13]. Several studies have demonstrated EBRs' positive impact on reducing the effects of drought stress by increasing the activity of antioxidant enzymes and the accumulation of proline. This reduces the production of reactive oxygen species and malondialdehyde (MDA) content [14–17]. Castañeda-Murillo et al. (2022) demonstrated that using EBR analogs can enhance plant tolerance to water deficiency by decreasing membrane lipid peroxidation, increasing photosynthetic pigments content, improving photosystem II efficiency, and promoting plants growth [14]. Likewise, Mohammadi and colleagues demonstrated a significant enhancement in leaf area, yield components, grain yield, protein content, and gas exchange rate in beans through the application of EBRs, under drought stress and normal conditions [18]. These findings highlight the potential of EBRs as an effective tool for enhancing multiple aspects of plants growth and productivity while mitigating the negative impacts of drought stress.

Growth promoters are small molecules that can elicit defense responses in plants [19] and stimulate the production of various secondary metabolites [20]. Yeast extract obtained from the yeast *Saccharomyces cerevisiae* is recognized as a biostimulant [21, 22]. In their research, Abdelaal et al. (2021) highlighted the potential of yeast extract in mitigating the negative impacts of drought stress on two wheat cultivars [23]. Furthermore, they reported positive effects of yeast extract on maize plants under drought stress [24]. These results revealed that yeast extract application shows promise as a beneficial intervention for enhancing plant tolerance and reducing the detrimental effects caused by drought stress. Additional research is required to gain a comprehensive understanding of the underlying mechanisms and to optimize the utilization of yeast extract in the cultivation of different crop species. Abdelaal et al. (2021) demonstrated that the using yeast extract alone or in combination with chitosan can enhance the proline content and activity of antioxidant enzymes while reducing oxidative stress caused by drought stress in garlic plants [25]. Similarly, Alzandi and Naguib (2022) reported that yeast treatment can boost antioxidant enzyme activities and reduce lipid peroxidation. These findings suggest that treating plants with yeast extract may be a viable strategy to improve plant tolerance under drought stress condition [26].

Cowpea (*Vigna sinesis* L.) is a fast-growing annual legume that is highly susceptible to drought stress during the pod-filling and flowering stages [27]. Water deficit stress during these stages has been shown to cause a 50% reduction in cowpea yield [28]. The application of EBR and yeast extract at different concentrations can ameliorate the negative impacts of water deficit stress on cowpea plants, leading to improved physiological and biochemical responses. The main goal of this study is to evaluate the impact of different levels of EBR and yeast extract on the morphophysiological and biochemical responses of cowpea plants under water deficit stress. By investigating these responses, we aim to elucidate the potential benefits and mechanisms underlying the utilizing these treatments in enhancing cowpea’s tolerance to water deficiency stress.

## Results and discussions

### Variance analysis of traits

This study aimed to assess the effects of different levels of EBR and yeast extract on the morphological traits, yield, yield components, and physiological characteristics of cowpea plants subjected to water deficiency stress. Supplementary Table S1 presents the results of the trait variance analysis. The year effect was found to be significant on yield, catalase activity, number of nodes, number of pods, weight of 20 pods, and weight of 20 seeds. Furthermore, the impact of different levels of water deficit was significant at the 5% level on various traits, including number of pods, leaf length, yield, chlorophyll a, chlorophyll b, total chlorophyll, carotenoids, proline, sugar, protein, flavonoids, MDA, catalase activity, superoxide dismutase, activities of guaiacol peroxidase, and ascorbate peroxidase (Supplementary Table S1).

Yeast extract and EBR levels had an impact on all physiological traits except polyphenol oxidase activity; however, only a few morphological and yield-related traits, including number of pods, leaf length, yield, weight of 20 pods, and weight of 20 seeds, were affected by these treatments. The interaction effect of EBR and yeast levels was significant on yield, leaf length, weight of 20 pods, weight of 20 seeds, and number of pods, but only polyphenol oxidase activity, superoxide dismutase activity, MDA, and total phenol were not affected by this interaction. In this study, the triple effects of the treatments had no significant impact on any of the investigated traits, except for total protein, flavonoids content, activities of ascorbate peroxidase, guaiacol peroxidase, and catalase, and weight of 20 pods. However, after comparing the averages, the triple effects of the studied treatments were significant for most of the characteristics (Supplementary Table S1).

### Morphological traits, yield and, yield components

To alleviate the detrimental effects of water deficiency on plants, several strategies are being implemented. These include the exogenous application of diverse chemicals or phytohormones through various methods. One particularly effective method is the exogenous/foliar application of these compounds, where they can be readily absorbed by plant leaves and subsequently transported to other parts of the plant. This allows them to regulate cellular metabolism and alleviate the negative effects caused by environmental stresses. By utilizing this approach, researchers aim to enhance plants tolerance and improve their ability to withstand challenging environmental conditions [26, 29]. Previous studies have highlighted the beneficial effects of EBRs and yeast extract in promoting plants growth under drought and water deficit stresses [30, 31]. However, limited information is available regarding the specific mechanisms through which EBRs and yeast extract improve cowpea growth under water deficit condition. In this study, we explored the potential of two growth stimulants, yeast extract and EBR, to mitigate the detrimental impacts of water deficit stress on cowpea plants. We assessed a range of parameters related to their growth, yield, yield components, photosynthetic capacity, osmotic adjustment, and antioxidant system under water deficit stress.

The mean comparison of the simple effect of year, different yeast extract levels, EBR, and water deficit on evaluated traits of cowpea is represented in Supplementary Tables S2, S3, S4 and S5. The treatment with normal irrigation resulted in the highest yield (1982.37 kg/ha) and weight of 20 seeds (32.89 g), while the treatment with a 16-day irrigation period resulted in the lowest yield (1320.32 kg/ha) and weight of 20 seeds (22.38 g). Under normal irrigation condition, the highest pods number per plant (11) was observed, whereas severe water deficiency treatment led to a lower number of pods (7) (Supplementary Table S3). The findings of this study indicated that water deficiency had a significant negative impact on most morphological traits, yield, and yield components in cowpea (Supplementary Table S3), which is consistent with the findings of other studies on the negative effects of water deficit stress on various plants [4, 23–25, 32–34]. These findings indicate that the impact of drought stress on plants is not limited to a specific species but can be observed across different types of crops. Understanding these common responses to water deficit stress can help researchers and farmers develop effective strategies for mitigating the detrimental effects and improving the tolerance of various plant species under challenging environmental conditions [4, 23, 25, 34]. To cope with environmental stresses, plants employ various mechanisms, including changes in morphological traits, and physiological and biochemical reactions [35].

The utilization of yeast extract led to a notable increase in the number of pods (28%), yield (14%), weight of 20 seeds (25%), and weight of 20 pods (18%) compared to the absence of yeast extract application (under normal irrigation). In our study, the application of yeast extract through spraying was found to alleviate the negative water deficit stress effects on morphological characteristics, yield, and yield components as demonstrated in Supplementary Table S5 and Table 1. Previous studies have shown that the utilizing yeast extract can effectively increase the yield and dry weight of cucumber and the photosynthetic pigments, potassium, phosphorus, and nitrogen content [36]. In the current study, the application of yeast extract resulted in an increase in leaf tissue (Supplementary Table S5), which may be attributed to the enhancement of auxin and cytokinin levels in the plant, leading to improvements in elongation and cell division [36]. The highest number of pods, yield, number of seeds in pods, weight of 20 pods, and weight of 20 seeds were obtained at 12 g of yeast extract, with values of 9.65, 1684.91 kg/ha, 8.55, 35.61 g, and 15.34 g, respectively (Supplementary Table S5). Yeast extract have been recognized as the substance that can improve both the yield and quality of various crops, such as tomato [37], orange trees [38], and wheat [23], in both favorable and challenging growing conditions. These results highlight the potential of yeast extract as a valuable tool for improving crop productivity and tolerance under challenging environmental conditions [37]. Numerous studies have shown that the foliar application of yeast extract has a beneficial effect on tomato plants cultivated under low-temperature conditions during the winter season. This application method has been found to significantly enhance vegetative growth, increase yield, and improve various fruit quality parameters [39]. In this study, the application of yeast extract has been shown to contribute to the recovery of cowpea plants' growth and seed yield components, suggesting that these growth regulators possess mechanisms that can mitigate the detrimental effects of water deficit stress. This recovery can be attributed to the presence of growth-related metabolites in yeast extract, including dissolved substances such as proteins, total soluble sugars, free amino acids, and B-group vitamins. These compounds play a crucial role in supporting the growth and development of cowpea plants, even under water deficit condition [40, 41]. By providing essential nutrients and promoting physiological processes, yeast extract aids in the restoration of cowpea plants’ growth and development, enabling them to overcome the challenges posed by water deficit stress.

**Table 1:** The mean comparison of interaction effects of different levels of water deficit and yeast extract on evaluated traits of cowpea

Treatment		PH	NN	NL	SD	PD	PL	PW20	GW20	NP
**Irri**	**yeast**									
**8**	**0**	26.89 ± 8.82 ab	10.96 ± 0.78 a	2.01 ± 0.1 bc	7.19 ± 1 b	6.93 ± 0.62 a	12.28 ± 1.08 ab	33.06 ± 1.39 a	14.57 ± 2.62 a	9.89 ± 0.69 a
	**12**	27.34 ± 5.96 a	11.21 ± 0.85 a	3.58 ± 0.45 a	9.69 ± 0.54 a	7.09 ± 0.35 a	12.69 ± 3.29 a	35.14 ± 1.06 a	14.71 ± 3.68 a	10.18 ± 1.12 a
**12**	**0**	25.74 ± 7.65 ab	10.86 ± 2.35 a	1.97 ± 0.21 bc	7.07 ± 0.93 b	6.31 ± 0.69 a	11.69 ± 0.95 ab	28.01 ± 1.27 b	14.21 ± 2.53 a	8.02 ± 0.59 b
	**12**	26.09 ± 5.18 ab	11.38 ± 0.81 a	2.38 ± 0.42 b	7.16 ± 0.67 b	7.14 ± 1.23 a	11.75 ± 1.2 ab	30.41 ± 1.29 b	14.29 ± 2.51 a	8.29 ± 0.5 b
**16**	**0**	23.9 ± 6.24 b	10.7 ± 0.65 a	1.86 ± 0.15 c	6.91 ± 0.66 b	6.06 ± 0.31 a	11.36 ± 0.8 b	28.82 ± 1.06 b	14.04 ± 1.77 a	7.11 ± 0.72 c
	**12**	26.01 ± 7.72 ab	11.12 ± 0.73 a	1.9 ± 0.44 c	7.22 ± 1.11 b	6.96 ± 0.46 a	11.47 ± 1.2 b	29.94 ± 1.12 b	14.4 ± 1.86 a	7.47 ± 0.59 c

		LL	Yield	LW	NGP	Chla	Chlb	Total Chl	Car	Proline	Sugar
**8**	**0**	6.66 ± 0.81 ab	1960.38 ± 441.55 a	5.15 ± 0.78 ab	9.37 ± 1.24 a	12 ± 1.23 b	6.05 ± 0.76 b	18.05 ± 1.82 b	13.37 ± 0.76 b	2.52 ± 0.48 f	3.25 ± 0.43 f
	**12**	7.13 ± 0.97 a	1974.94 ± 431.99 a	6.36 ± 0.29 a	10.42 ± 1.17 a	13.42 ± 1.83 a	6.71 ± 1.17 a	20.14 ± 2.67 a	14.35 ± 1.87 a	2.98 ± 0.63 e	4.01 ± 0.68 e
**12**	**0**	6.09 ± 0.91 c	1387.61 ± 251.22 b	3.85 ± 0.57 b	8.25 ± 1.14 ab	10.38 ± 1.37 d	4.48 ± 0.76 d	14.87 ± 1.99 d	11.02 ± 1.11 d	3.39 ± 0.7 d	4.58 ± 0.59 d
	**12**	6.65 ± 1.07 b	1455.79 ± 302.96 b	4.71 ± 0.65 ab	9.1 ± 1.31 ab	11.33 ± 2.24 c	5.25 ± 1.03 c	16.58 ± 3.14 c	11.66 ± 1.71 c	3.82 ± 0.79 c	5.29 ± 0.86 c
**16**	**0**	5.74 ± 0.64 c	1309.43 ± 395.95 b	3.73 ± 0.54 b	6.23 ± 1.09 b	6.77 ± 1.3 f	2.77 ± 0.64 e	9.55 ± 1.83 f	7.77 ± 0.94 f	4.28 ± 1.04 b	5.75 ± 0.7 b
	**12**	5.91 ± 0.64 c	1331.2 ± 239.16 b	4.23 ± 0.61 b	8.27 ± 1.44 ab	8.72 ± 2.49 e	4.2 ± 1.06 d	12.93 ± 3.45 e	8.38 ± 1.81 e	5.04 ± 1.46 a	6.9 ± 0.89 a

		Phenol	Protein	Flavonoid	MDA	SOD	APX	GPX	CAT	PPO
**8**	**0**	23.55 ± 4.44 b	137.77 ± 22.27 d	11.94 ± 1.47 f	6.03 ± 0.67 de	0.033 ± 0.004 f	0.05 ± 0.008 f	0.04 ± 0.004 f	0.023 ± 0.003 e	0.027 ± 0.005 e
	**12**	28.57 ± 5.21 b	175.78 ± 31.33 c	14.98 ± 2.08 e	5.42 ± 0.63 e	0.038 ± 0.004 e	0.06 ± 0.009 e	0.05 ± 0.001 d	0.029 ± 0.007 d	0.039 ± 0.009 c
**12**	**0**	30.5 ± 5.94 b	163.38 ± 22.67 c	17.61 ± 1.81 d	7.98 ± 1.34 c	0.042 ± 0.004 d	0.06 ± 0.009 d	0.05 ± 0.007 e	0.063 ± 0.008 a	0.035 ± 0.005 d
	**12**	37.16 ± 6.55 ab	213.94 ± 54.48 b	19.72 ± 1.9 c	6.68 ± 1.12 d	0.049 ± 0.006 c	0.09 ± 0.002 b	0.07 ± 0.001 b	0.034 ± 0.005 c	0.052 ± 0.001 b
**16**	**0**	49.38 ± 53.45 a	205.11 ± 55.17 b	24.38 ± 4.38 b	11.33 ± 3.07 a	0.055 ± 0.001 b	0.08 ± 0.006 c	0.06 ± 0.001 c	0.033 ± 0.006 c	0.053 ± 0.004 b
	**12**	54.72 ± 5.3 a	277.66 ± 65.96 a	30.66 ± 4.01 a	10 ± 1.81 b	0.061 ± 0.007 a	0.11 ± 0.005 a	0.1 ± 0.003 a	0.043 ± 0.008 b	0.064 ± 0.001 a

Mean comparison was performed by LSD method at 5% probability. Columns with similar letters did not differ significantly

*Irri* Different levels of water deficit, *PH* Plant height, *NN* Number of nodes, *NL* Distance internode, *SD* Stem diameter, *PD* Pod diameter, *PL* Pod length, *PW 20* Weight 20 pods, *GW 20* Weight 20 grains, *NP* Number of pods, *LL* Leaf length, *Yi* Yield, *LW* Leaf width, *NGP* Number of seeds in pods, *Chla* Chlorophyll a, *Chlb* Chlorophyll b, *ChlT* Total chlorophyll, *Car* Carotenoid, *MDA* Malondialdehyde, *CAT* Catalase, *SOD* Superoxide dismutase, *GPX* Guaiacol peroxidase, *APX* Ascorbate peroxidase, *PPO* Polyphenol oxidase

In a study, it was observed that the foliar application of yeast extract had a significant positive impact on various parameters of grains wheat. Specifically, it led to notable enhancements in plant height, spike length, chlorophylls content, and nutrient content [42]. This is attributed to the presence of easily absorbable amino acids in the extract, which enable plants to conserve energy and accelerate their growth or recovery, particularly during critical stages of plant development. By providing readily available nutrients, yeast extract supports and promotes the overall growth and development of plants, contributing to their robustness and tolerance [42, 43]. It has been indicated that exogenous application of yeast extract has a significant positive impact on various growth characteristics, including the leaves number, branches number, shoot length, and shoot fresh and dry weight. Yeast extract may influence nutrient signal transduction, resulting in the production of growth-promoting compounds and the mitigation of stress-induced toxicity [23, 25, 44, 45].

The employing varying EBR concentrations (especially 10 µm) resulted in a significant increase in the number of pods (35%), yield (7%), and weight of 20 pods (27%) compared to the absence of EBR application. The highest number of pods, yield, number of seeds in pods, the weight of 20 pods, and weight of 20 seeds were obtained at the EBR level of 10 µl, with values of 11, 1611.79 (kg/ha), 11, 35.6 g, and 16.81 g, respectively (Supplementary Table S4). The mean comparison of the treatments’ double interaction effects on evaluated cowpea traits is represented in Supplementary Tables S6, S7, S8 and S9. In plants subjected to severe water deficit stress, the application of 10 µl of EBR resulted in a 10%, 8%, 10%, 11%, 10, and 7% increase in the number of pods, leaf length, yield, number of seeds in pods, weight of 20 pods and weight of 20 seeds, respectively, compared to the absence of EBR application in the same irrigation treatment (Table 2). In the current study, the application of yeast extract or EBR, whether used alone or in combination, significantly enhanced the morphological characteristics, yield, and yield components of cowpea plants under water deficit condition. This increase can be attributed to the synergistic effects of yeast extract and EBR in promoting growth and increasing leaf numbers, and plant dry weight compared (Supplementary Tables S5 and S4). These results align with previous findings reported by El-Shawa et al. [44] and Abdelaal et al. [23]. The prior studies have shown that the EBR utilization alone or in combination with other hormones and growth regulators can increase plant yield and stress tolerance [13, 40]. Applying these growth stimulants from pollination to seed ripening has been proven to enhance yield by improving the seed-filling process, delaying senescence, promoting storage and remobilization of nutrients, and preserving cell membrane integrity [46]. These findings highlight the positive effects of using growth stimulants throughout critical stages of plant development.

**Table 2:** The mean comparison of interaction effects of different levels of water deficit and 24-epibrassinolide (B) on evaluated traits of cowpea

Treatment		PH	NN	NL	SD	PD	PL	PW20	GW20	NP	LL
**Irri**	**B**										
**8**	**0**	26.16 ± 7.03 ab	11.11 ± 0.61 a	2.28 ± 1.4 ab	7.26 ± 0.71 b	7.11 ± 0.33 a	12.09 ± 0.93 a	31.7 ± 7.53 abcd	13.97 ± 1.82 ab	9.48 ± 1.03 b	6.62 ± 1.15 ab
	**5**	27.7 ± 8.53 a	11.05 ± 0.58 a	2.74 ± 2.79 ab	7.34 ± 0.81 b	7.08 ± 0.35 a	12.16 ± 1.54 a	32.97 ± 5.36ab	14.7 ± 2.17 ab	10.14 ± 0.93 a	6.29 ± 0.84 bc
	**10**	28.1 ± 6.93 a	11.45 ± 0.69 a	3.16 ± 7.88 a	8.99 ± 0.19 a	7.34 ± 1.52 a	12.28 ± 3.98 a	34.63 ± 9.56 a	15.44 ± 3.72 a	10.49 ± 0.64 a	6.92 ± 1.13 a
**12**	**0**	25.75 ± 7.89 ab	10.86 ± 0.92 a	1.93 ± 1.17 ab	7.07 ± 1.06 b	6.89 ± 0.36 a	11.76 ± 1.52 a	28.75 ± 5.47 de	13.76 ± 1.54 ab	8.05 ± 0.78 c	6.12 ± 1 bc
	**5**	26.51 ± 6.24 ab	10.96 ± 0.71 a	1.94 ± 1 ab	7.2 ± 0.86 b	6.92 ± 0.25 a	11.88 ± 0.89 a	29.36 ± 10.69 cde	14.32 ± 2.36 ab	8.14 ± 0.29 c	6.17 ± 0.51 bc
	**10**	26.57 ± 8.97 ab	11.43 ± 0.82 a	2.07 ± 1.28 ab	7.27 ± 1.16 b	6.96 ± 0.61 a	12.2 ± 0.93 a	32.46 ± 10.52 abc	14.55 ± 1.81 ab	8.27 ± 0.52 c	6.57 ± 0.84 ab
**16**	**0**	23.22 ± 6.5 b	10.64 ± 0.85 a	1.75 ± 0.91 b	6.83 ± 1.04 b	6.84 ± 0.78 a	11.21 ± 1.06 a	26.96 ± 5.34 e	13.62 ± 2.32 b	6.96 ± 0.75 d	5.96 ± 0.94 c
	**5**	25.25 ± 4.27 ab	10.67 ± 2.85 a	1.78 ± 0.97 b	6.7 ± 0.45 b	6.9 ± 0.44 a	11.64 ± 1 a	28.21 ± 4.86 de	14.09 ± 2.68 ab	7.45 ± 0.67 d	6.27 ± 0.129 bc
	**10**	25.31 ± 6.25 ab	11.26 ± 0.78 a	1.89 ± 1.3 ab	7.2 ± 0.54 b	6.94 ± 0.55 a	11.67 ± 1.26 a	30.04 ± 6.41 bcde	14.51 ± 3.63 ab	7.54 ± 0.51 d	6.36 ± 0.66 abc

		Yield	LW	NGP	Chla	Chlb	Total Chl	Car	Proline	Sugar
**8**	**0**	1803.31 ± 335.04 b	4.42 ± 0.67 a	9.29 ± 1.2 b	10.97 ± 0.77 c	5.45 ± 0.5 d	16.42 ± 1.02 d	12.44 ± 0.68 c	2.09 ± 0.13 h	3.01 ± 0.27 i
	**5**	2030.17 ± 521.32 a	4.7 ± 0.82 a	9.46 ± 1.34 b	13.08 ± 1.37 b	6.54 ± 0.78 b	19.62 ± 1.82 b	13.97 ± 0.74 b	2.76 ± 0.38 f	3.59 ± 0.41 h
	**10**	2069.5 ± 407.33 a	4.95 ± 0.91 a	10.41 ± 0.71 a	14.08 ± 1.31 a	7.16 ± 0.83 a	21.25 ± 1.58 a	15.16 ± 1.58 a	3.4 ± 0.31 e	4.29 ± 0.6 f
**12**	**0**	1393.28 ± 204.66 cd	4.21 ± 0.66 a	8.96 ± 1.28 b	8.83 ± 0.83 e	3.96 ± 0.27 f	12.8 ± 0.81 f	9.79 ± 0.78 d	2.69 ± 0.19 f	4.08 ± 0.26 g
	**5**	1378.88 ± 320.61 cd	4.23 ± 0.5 a	9.18 ± 1.79 b	11.16 ± 1.26 c	4.76 ± 0.73 e	15.93 ± 1.87 d	11.83 ± 1.02 c	3.67 ± 0.28 d	4.95 ± 0.33 e
	**10**	1522.88 ± 318.79 c	4.57 ± 0.75 a	8.64 ± 1.57 b	12.58 ± 1.16 b	5.87 ± 0.65 c	18.45 ± 1.45 c	12.41 ± 0.99 c	4.45 ± 0.29 c	5.78 ± 0.59 c
**16**	**0**	1243.18 ± 458.92 d	4.05 ± 0.77 b	7.85 ± 1.19 b	5.5 ± 0.52 f	2.62 ± 0.56 h	8.12 ± 0.75 h	6.75 ± 0.75 f	3.16 ± 0.17 f	5.41 ± 0.5 d
	**5**	1324.49 ± 257.43 cd	4.2 ± 0.9 a	8.29 ± 0.99 b	8.16 ± 1.74 e	3.35 ± 0.7 g	11.52 ± 2.41 g	7.91 ± 0.66 e	4.83 ± 0.63 b	6.32 ± 0.61 b
	**10**	1363.54 ± 148.18 cd	4 ± 0.5 b	8.39 ± 1.71 b	9.58 ± 1.62 d	4.49 ± 1.15 e	14.07 ± 2.67 e	9.58 ± 1.16 d	5.99 ± 0.78 a	7.26 ± 0.76 a

		Phenol	Protein	Flavonoid	MDA	SOD	APX	GPX	CAT	PPO
**8**	**0**	20.19 ± 2 c	128.85 ± 12.07 e	11.73 ± 1.36 g	6.28 ± 0.69 e	0.03 ± 0.002 f	0.04 ± 0.004 h	0.039 ± 0.003 f	0.02 ± 0.001 g	0.025 ± 0.004 f
	**5**	26.58 ± 3.55 bc	151.08 ± 24.28 d	13.08 ± 1.78 g	5.75 ± 0.44 ef	0.037 ± 0.004 e	0.05 ± 0.005 g	0.048 ± 0.008 e	0.024 ± 0.002 f	0.033 ± 0.007 e
	**10**	31.41 ± 2.93 bc	190.41 ± 24.44 c	15.58 ± 2.1 f	5.15 ± 0.46 f	0.04 ± 0.002 e	0.06 ± 0.006 f	0.062 ± 0.01 d	0.033 ± 0.006 d	0.042 ± 0.009 d
**12**	**0**	27 ± 2.76 bc	148.91 ± 14.27 d	16.66 ± 1.3 f	8.58 ± 1.05 c	0.04 ± 0.003 e	0.05 ± 0.003 g	0.05 ± 0.005 e	0.08 ± 0.009 a	0.033 ± 0.005 e
	**5**	33.08 ± 4.69 bc	181.5 ± 16.9 c	18.58 ± 1.31 e	7.41 ± 0.9 d	0.044 ± 0.003 d	0.08 ± 0.009 d	0.064 ± 0.001 cd	0.03 ± 0.004 e	0.043 ± 0.008 d
	**10**	41.41 ± 3.65 b	235.58 ± 49.72 b	20.75 ± 1.35 d	6 ± 0.75 ef	0.052 ± 0.005 c	0.1 ± 0.02 b	0.079 ± 0.01 b	0.036 ± 0.005 c	0.053 ± 0.001 b
**16**	**0**	34.41 ± 4.39 bc	197.16 ± 12.31 c	24.58 ± 2.27 c	12.5 ± 1 a	0.05 ± 0.002 c	0.08 ± 0.004 c	0.066 ± 0.007 c	0.032 ± 0.004 d	0.046 ± 0.006 c
	**5**	40.33 ± 4.84 b	234.08 ± 33.01 b	27.58 ± 2.64 b	11.16 ± 1.11 b	0.056 ± 0.004 b	0.07 ± 0.004 e	0.083 ± 0.001 b	0.035 ± 0.004 c	0.053 ± 0.001 b
	**10**	66.41 ± 61.65 a	292.91 ± 96.95 a	30.41 ± 7.48 a	8.33 ± 3 cd	0.067 ± 0.001 a	0.13 ± 0.04 a	0.11 ± 0.004 a	0.048 ± 0.009 b	0.056 ± 0.007 a

Mean comparison was performed by LSD method at 5% probability. Columns with similar letters did not differ significantly

*Irri* Different levels of water deficit, *B* Different 24-epibrassinolide levels, *PH* Plant height, *NN* Number of nodes, *NL* Distance internode, *SD* Stem diameter, *PD* Pod diameter, *PL* Pod length, *PW 20* Weight 20 pods, *GW 20* Weight 20 grains, *NP* Number of pods, *LL* Leaf length, *Yi* Yield, *LW* Leaf width, *NGP* Number of seeds in pods, *Chla* Chlorophyll a, *Chlb* Chlorophyll b, *ChlT* Total chlorophyll, *Car* Carotenoid, *MDA* Malondialdehyde, *CAT* Catalase, *SOD* Superoxide dismutase, *GPX* Guaiacol peroxidase, *APX* Ascorbate peroxidase, *PPO* Polyphenol oxidase

### Physiological and biochemical traits

#### Pigments content

In the our study, the water deficiency stress (especially severe water deficiency) resulted in a substantial reduction in the concentrations of chlorophyll a (41%), chlorophyll b (44%), total chlorophyll (42%), and carotenoids (43%) in cowpea plants during both seasons (supplementary Table S3). This decline in chlorophyll contents may be attributed to the damaging effects of water deficiency on chlorophyll pigments, which leads to the disruption of light-harvesting protein complexes. Consequently, it reduces carbon dioxide fixation and hampers NADP^+^ production through the Calvin cycle pathway [47, 48]. Our discoveries align with the findings of prior researches [4, 34, 49]. Furthermore, Gedam et al. [50] reported negative impacts on membrane stability index, relative water content, total chlorophyll content, antioxidant enzyme activities, and bulb yield in onion plants subjected to drought stress. However, the application of EBR and yeast extract effectively reduced chlorophyll degradation under water deficiency as shown in supplementary Tables S4 and S5. The preservation or reduction of chlorophyll degradation in plants treated with EBR is likely due to the plant's increased tolerance to oxidative stress, as EBR has antioxidant properties [51]. Spraying EBR (especially 10 µm) on plants subjected to severe water stress treatment resulted in a significant increase of 70%, 71%, 70%, and 41% in the content of chlorophyll a, chlorophyll b, total chlorophyll, and carotenoid, respectively, compared to no EBR spraying (Table 2).

Remarkably, the application of yeast extract to cowpea plants subjected to water deficit stress resulted in a noteworthy enhancement of photosynthetic pigments. This increase in chlorophyll content can be attributed to yeast extract ability to promote chlorophyll formation while simultaneously inhibiting its degradation. Moreover, yeast extract positive impact on chlorophyll concentrations can be attributed to the hormones it produces, such as indole acetic acid (IAA), which facilitate plant growth and provide essential nutrition. These beneficial effects enable cowpea plants to effectively counteract the harmful effects of various toxic compounds, including reactive oxygen species (ROS). By bolstering chlorophyll levels and supporting plant health, yeast extract plays a crucial role in enhancing the resilience and yield cowpea plants under water deficit stress condition [52]. Carotenoids have the ability to reduce the formation of singlet oxygen species through direct or indirect pathways, neutralizing singlet oxygen and reducing the excited triplet state of chlorophyll [53]. Previous studies have shown that EBR can promote the synthesis of carotenoids, inhibiting various reactive oxygen species [51]. The increase in photosynthetic pigment content observed after the yeast extract application in this study is likely due to the cytokinin present in the extract, which can delay leaf senescence [54]. The foliar application of yeast extract has been found to improve various physiological properties in plants. This improvement can be attributed to the bio-regulatory role of yeast extract, which affects the balance between photosynthesis and photorespiration [55]. Additionally, yeast extract has been shown to delay leaf senescence by reducing chlorophyll degradation and enhancing protein and RNA synthesis [55]. Yeast extract also plays a significant role in increasing carbon dioxide release through the fermentation process, which in turn enhances the synthesis of photosynthetic pigments and activates the photosynthesis process. Similar findings have been previously reported for various plants, including flax [56], wheat [42], Chinese carnations [43], and white lupines [57]. In the current investigation, the enhanced vegetative growth and yield of stressed cowpea plants can be partially attributed to the elevated levels of total chlorophyll content and increased antioxidant activities resulting from yeast extract and EBR application.

#### Lipid peroxidation

In our study, we noted a substantial increase in the MDA levels (80%) under water deficit-stressed cowpea plants compared to the control group (supplementary Table S3). The observed elevation in levels can be attributed to the oxidative stress experienced by plant cells under water deficit conditions, which has a detrimental effect on plasma membranes and their permeability. The concurrent increase in proline levels along with MDA serves as an indicator of ongoing oxidative damage within the plants [5, 58]. These findings highlight the detrimental impact of water deficit stress on cowpea plants and emphasize the importance of mitigating oxidative stress to maintain cellular integrity and function. However, the application of yeast extract reduced the content of MDA (20%) in plants grown under water deficiency (supplementary Tables S4 and S5), indicating that this substance has a positive effect on improving plant tolerance by reducing oxidative stress [17, 59]. In our study, the application of EBR (especially 10 µm) enabled the plants to effectively mitigate water deficit stress, resulting in reduced MDA levels (29%) compared to untreated plants. The reduction in lipid peroxidation attributed to EBR was associated with heightened enzymatic antioxidant activities, leading to an improvement in membrane permeability. These results are in line with previous findings reported in [46, 59–61].

#### Total protein and activity of antioxidant enzymes

In the current study, a notable increase in total protein content and antioxidant enzyme activities was observed in water-deficit-stressed cowpea plants when compared to the control group (Supplementary Tables S3). These elevated levels indicate the occurrence of oxidative damage in plants experiencing abiotic stresses [62]. Similar findings have been reported in various plant species under different conditions of abiotic stress [4, 58, 63, 64] and biotic stress [65]. Our results align with previous research conducted by Hafez et al. [34], who noted substantial increases in MDA level and reactive oxygen species in water deficit-stressed barley plants due to damage to plasma membranes and cytoplasm. These collective findings emphasize the detrimental effects of water deficit-induced oxidative stress on plant physiology and highlight the importance of implementing strategies to mitigate such damage for optimal plant health and performance.

The application of EBR increased the activity of antioxidant enzymes in cowpea under normal irrigation condition and water deficit stress. Spraying EBR (especially 10 µm) on plants under water stress treatment resulted in a significant increase of 43%, 22%, 60%, 34%, and 66% in the activities of catalase (CAT), polyphenol oxidase (PPO), ascorbate peroxidase (APX), superoxide dismutase (SOD), and guaiacol peroxidase (GPX), respectively, compared to non-use (Table 1). This enhancement can be attributed to the influence of EBR on the transcription and/or translation of antioxidant genes [66]. The upregulation of these antioxidant enzymes signifies their crucial role in scavenging reactive oxygen species and protecting plant cells from oxidative damage caused by water deficit stress, as evidenced by lower level of MDA in cowpea leaves. Previous studies have demonstrated that treating maize [11]*,* rice plants [67], *Prunus persicae* [68], and tomato [61] plants with EBR can increase the activity of antioxidant enzymes and improve plant tolerance under drought stress condition. Studies have reported that treating plants with EBR can regulate the expression of genes involved in producing catalase, superoxide dismutase, and ascorbate peroxidase, resulting in increased efficiency in water and carbon dioxide use and improved activity of both enzymatic and non-enzymatic antioxidants under abiotic stress [17, 69–71].

Researches have demonstrated that the yeast extract application significantly enhances the activity of peroxidase and catalase enzymes in tomato leaves when exposed to low temperatures. Notably, the highest activities of peroxidase and catalase were observed in tomato plants treated with yeast extract at a concentration of 9 g/L, surpassing both the control group and other treatment groups in both seasons. These findings highlight the significant role of yeast extract in enhancing the enzymatic activity associated with stress response mechanisms in plants, particularly under low-temperature condition [39].

#### Soluble sugar, total proline, phenol and flavonoids content

In our study, we observed significant changes in the soluble sugars and proline content of cowpea plants under water deficit condition (Supplementary Table S3). These alterations are believed to play a crucial role in osmotic adjustment and can potentially influence genes expressions related to plant metabolism, storage, and defense functions either directly or indirectly [72]. The modifications in soluble sugar levels highlight the dynamic nature of plant responses to water deficit stress and suggest their involvement in regulating various physiological processes that contribute to plant adaptation and survival under challenging environmental conditions. Similar to soluble sugars, the accumulation of free proline plays a significant role in osmotic adjustment under water deficit stress. This accumulation is an adaptive response aimed at compensating for plant survival and aiding in drought resistance [73]. Free proline contributes to enhancing plant tolerance by detoxifying ROS and can also physically quench singlet oxygen (_1_O^2^) or directly react with hydroxyl radicals (OH^−^) [74]. Simultaneously applying EBR (especially 10 µm) and yeast extract under water stress condition resulted in a significant increase of 70% and 45% in phenol and 70% and 47% in proline content, respectively, compared to non-application (Tables 1 and 2). Talaat and Shawky [46], as well as Chen et al. [7], illustrated that the application of EBR promoted proline biosynthesis within plant cells. The exogenous EBR application has been shown to increase proline and soluble sugars content, leading to improved tolerance to drought stress in tomato [71]. By promoting proline biosynthesis, EBR treatment effectively strengthens plants’ capacity to withstand water deficit condition and safeguards them against the detrimental effects of oxidative stress. These noteworthy findings underscore the potential of EBR as a valuable tool for bolstering plant resilience when faced with drought-induced stress [18, 30, 75].

Yeast extract is a rich source of amino acids, especially proline, which can increase proline content in plants. A study found that treating wheat with yeast extract increased the plant's endogenous proline content [76]. A recent study has revealed that seed priming with yeast extract has a positive impact on the antioxidant capacity of maize plants when subjected to salt stress. This improvement is attributed to the enhancement of ascorbic acid (AsA) levels and total phenolic compounds, which play a crucial role in reducing oxidative stress and enhancing the plants' tolerance to salt stress. By effectively reducing the oxidative burden, yeast extract seed priming offers a promising approach to improve the resilience and survival of maize plants under salt stress [77]. This present study observed a significant increase in flavonoids content of cowpea plants treated with water deficiency (90%), different levels of EBR (30%) and yeast extract (25%), and their combination (155%) compared to plants grown under normal condition (supplementary tables S3, S4, and S10). The induction of defense reactions in the plant by each of these factors leads to metabolic changes, including the production of flavonoids and phenolic compounds [78]. Recent findings have uncovered that the utilization of various treatments of yeast extract has led to significant improvements in the growth characteristics, anatomical structure, physiological traits, and yield of treated *Lupinus termis* L. Notably, among the different doses of yeast extract, a concentration of 75 mL per liter demonstrated remarkable enhancements in growth characteristics, leaf chlorophyll contents, total soluble sugars, soluble protein, and seed yield. These findings have led researchers to conclude that utilizing an adequate dose of yeast extract can effectively enhance salinity stress tolerance in *Lupinus termis* L, offering a promising strategy to improve their resilience and productivity in salinity-stressed environments [57]. Yeast extract induces the production of endogenous hormones, leading to the accumulation of secondary metabolites like total soluble sugars, phenolic compounds, flavonoids, and glycosides [79].

The precise mechanisms underlying this phenomenon are still being investigated, but studies suggest that EBR's impact on plant growth and tolerance is multifaceted and contributes significantly to enhancing stress tolerance [31]. Previous studies have revealed the effectiveness of foliar or EBR exogenous application in regulating plant development and physiological processes under biotic and abiotic stresses [18, 30, 31]. These reports highlight the potential of EBR as a valuable tool for enhancing plant tolerance and improving yield when faced with various environmental stresses. The triple interaction effects of the treatments on most of the investigated traits in this research were not significant, although the average comparison results showed that these effects are significant for most of the traits (supplementary Tables S10, S11, S12, S13 and S14).

#### Principal component analysis

Hosseini et al. (2018) employed principal component analysis (PCA) to identify the most significant traits in the data set and to gain a better understanding of the trends and relationships among these traits for the genotypes [80]. Apart from its applications in grouping and clustering, PCA can also be employed to quantify variability in different groups of variables. Additionally, it can be used to test for differences in complex traits among groups of individuals by utilizing PC scores in univariate statistical analyses [81]. The PCA results revealed that the first six components accounted for 97.8% of the available variation, with the first two components explaining 57.16% of the total variation. The first component, responsible for 37.45% of the total diversity, included traits such as proline, sugars, phenol, total protein, flavonoids, antioxidant enzymes (excluding polyphenol oxidase), and leaf length. Meanwhile, the second component, which accounted for 19.71% of the variation, included traits such as chlorophylls content, MDA content, number of pods, weight of 20 pods, and yield. The third component, with a 18.48% share, included polyphenol oxidase, leaf width, plant height, number of nodes, pod diameter, pod length, and the number of seeds in pods.

The first component of the analyzed traits consisted of physiological characteristics, which are crucial defense mechanisms for plants under abiotic stress. Therefore, selecting superior genotypes based on physiological traits may be an important strategy for future breeding programs (Table 3). The presence of pigments content highlights the importance of increasing plant efficiency and yield. Therefore, selecting plants with higher pigments content under water stress condition may lead to the development of plants with higher yield. In a study aimed at assessing the impact of EBR on enhancing the tolerance of maize hybrids under water deficit and drought stress conditions, the relationships between various agronomic and physiological traits were examined using PCA. The analysis revealed that the first two principal components accounted for a significant portion of the variance, approximately 91.81%. Specifically, the first and second components contributed to 80.52% and 11.29% of the total variance, respectively [8].

**Table 3:** Eigen values, eigen vectors and cumulative variance of the investigated traits in this study

Traits	Components					
	**1**	**2**	**3**	**4**	**5**	**6**
**NP**	-.395	**.850**	-.144	.084	-.020	.070
**Chla**	.018	**.971**	-.059	-.031	-.036	-.192
**Chlb**	.003	**.989**	-.089	.034	.023	-.031
**Total Chl**	.013	**.984**	-.070	-.008	-.015	-.136
**Car**	-.203	**.954**	-.046	-.007	.038	-.150
**Proline**	**.939**	-.070	.245	-.031	-.066	-.151
**Sugar**	**.940**	-.257	.136	-.039	-.118	.047
**Phenol**	**.666**	-.148	.409	.027	-.135	.034
**Protein**	**.983**	.019	-.010	-.024	-.100	.053
**Flavo**	**.883**	-.424	.015	-.037	-.090	.094
**MDA**	.124	**-.927**	-.055	-.036	.024	.210
**SOD**	**.917**	-.272	.239	-.004	-.111	.057
**APX**	**.917**	-.050	-.007	-.073	.030	.067
**GPX**	**.972**	.012	-.103	-.081	-.075	.015
**CAT**	**.64**	-.134	.019	-.142	-.026	.103
**PPO**	.183	-.152	**.949**	.056	-.083	-.017
**LL**	**-.613**	-.187	.007	-.089	.649	-.138
**LW**	-.174	-.167	**.921**	.011	.153	-.093
**PH**	-.256	.303	**-.725**	.245	.203	-.022
**NN**	-.017	-.216	**-.814**	.169	.048	.035
**Nl**	-.172	.098	.025	.095	**.908**	-.037
**SD**	.048	-.325	-.124	**.761**	-.042	.075
**PD**	.130	.129	**.802**	.035	.200	.204
**PL**	-.243	-.141	**.691**	.437	.132	.049
**PW20**	-.018	**.656**	-.040	.485	.474	.081
**GW20**	.087	-.121	.206	**.680**	-.058	.091
**NGP**	.120	-.152	**.959**	.065	-.066	-.027
**Yield**	-.406	**.783**	-.189	.165	.160	.155
**Eigen values**	10.48	5.50	5.12	1.83	1.39	1.10
**Cumulative of variance (%)**	37.45	57.160	75.64	84.03	93.26	97.8

*PH* Plant height, *NN* Number of nodes, *NL* Distance internode, *SD* Stem diameter, *PD* Pod diameter, *PL* Pod length, *PW 20* Weight 20 pods, *GW 20* Weight 20 grains, *NP* Number of pods, *LL* Leaf length, *Yi* Yield, *LW* Leaf width, *NGP* Number of seeds in pods, *Chla* Chlorophyll a, *Chlb* Chlorophyll b, *ChlT* Total chlorophyll, *Car* Carotenoid, *MDA* Malondialdehyde, *CAT* Catalase, *SOD* Superoxide dismutase, *GPX* Guaiacol peroxidase, *APX* Ascorbate peroxidase, *PPO* Polyphenol oxidase

Eigenvalues are significant ≥ 0.50

Girgel (2021) reported that in their study on beans, the first and second components accounted for 38% and 20% of the total diversity, respectively, while the third component contained 16%. The first component included traits such as chlorophyll a and b, carotenoids, number of pods per plant, length of pods, and weight of 20 seeds, while the second component had the highest coefficients for MDA, plant height, number of seeds in pods, and proline content [82]. In another study, the first 7 components contributed 74%, with the first and second components accounting for 35% of the total diversity of bean genotypes. In this study, the first component had the highest coefficients for number of pods per plant, seed size, and seed yield traits, while the traits of 20 seed weight and internode distance had a greater contribution in the second component [83]. The examples provided demonstrate how PCA can be useful in identifying crucial traits associated with stress tolerance in beans. These findings also showcase the potential of PCA to inform breeding programs aimed at developing stress-tolerant cultivars.

**Fig. 1:**
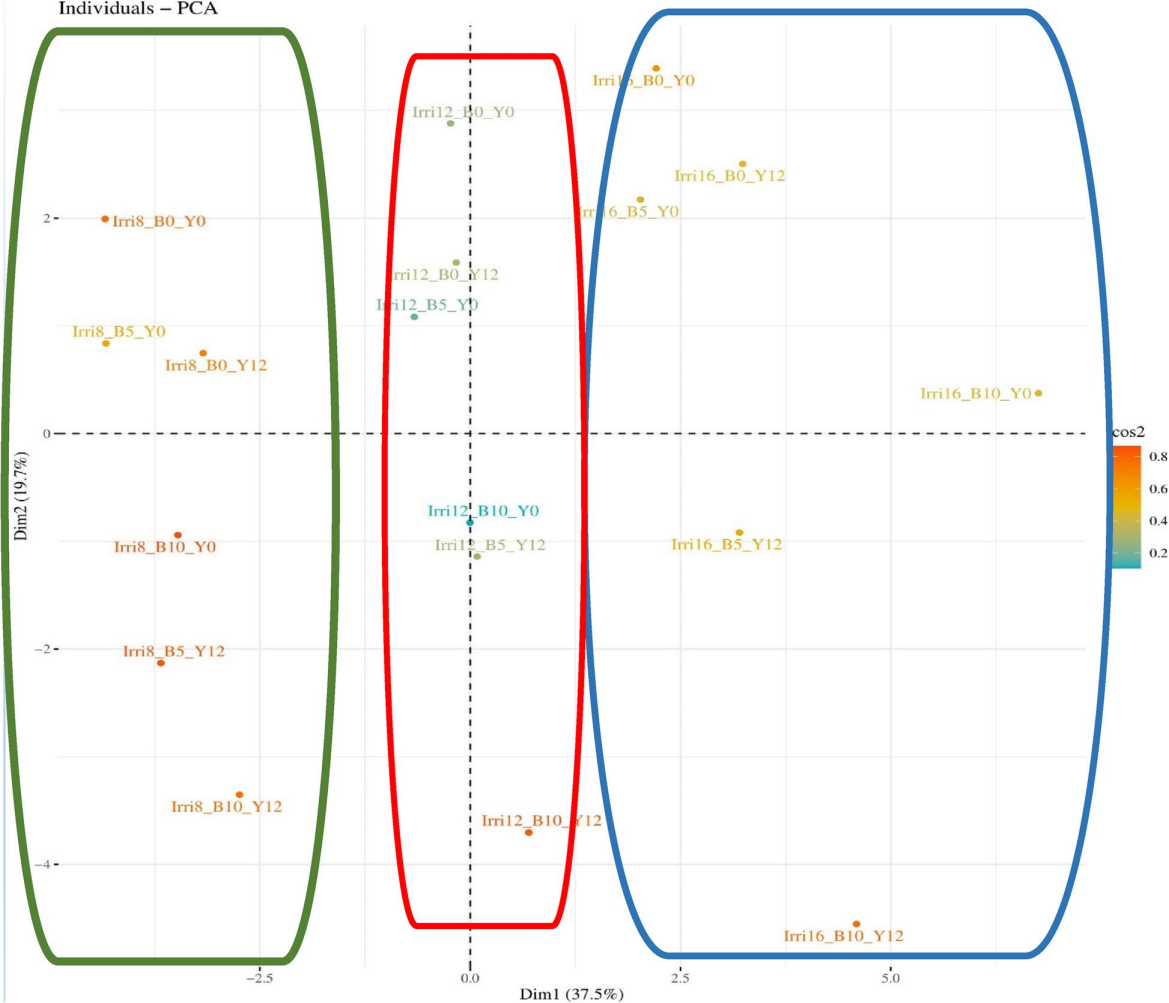
The distribution of treatments based on the first and second components

In current study, the PCA results indicated that the assessed treatments accounted for 57.2% of the total variation, with the first and second components contributing 37.5% and 19.7%, respectively. The first two components almost completely separated all the investigated treatments from each other, with the three irrigation treatments of 8, 12, and 16 days being grouped in separate clusters (Table 3). These findings were consistent with the results obtained from the heatmap, where the three irrigation treatments of 8, 12, and 16 days were separated and placed in distinct groups (Fig. 1).

#### Correlation between traits

Evaluating the correlation coefficients between different traits can facilitate making more accurate decisions about indirect selection indicators and eliminating ineffective traits. Correlation between traits may result from pleiotropy or high linkage between genes controlling these traits [84]. The results of the correlation analysis indicated a significant positive correlation between seed yield and pod length (0.45**), pod width (0.60**), weight of 20 seeds (0.67**), number of seeds in pods (0.65**), number of pods (0.90**), total chlorophyll (0.74**), carotenoids (0.82**), leaf length (0.67**), and leaf width (0.65**). The seed number in pods showed a significant positive correlation with total phenol (0.80**), leaf width (0.90**), and PPO activity (0.90**). The glutathione peroxidase activity exhibited a positive and significant correlation with total proline (0.88**), sugars (0.90**), protein (0.98**), flavonoids (0.87**), SOD activity (0.85**), and APX activity (0.91**).

Moreover, there was a significant negative correlation between MDA and chlorophyll a (-0.93**), chlorophyll b (-0.93**), total chlorophyll (-0.94**), carotenoids (-0.93**), and yield (-0.66**) (Table 4). Earlier research has shown that there is a strong correlation between the number of seeds per plant and per pods with yield, which makes them a feasible indirect selection index for genotypes with high yield [60]. In rice plants, it has been demonstrated that there is a positive or negative significant correlation between root-related traits with certain antioxidant enzymes [85].

**Table 4:** The correlation coefficients among the investigated traits in this study

	NP	Chla	Chlb	ToChl	Car	Prolin	Suger	Phenol	Protein	Flav	MDA	SOD	APX	GPX	CAT
NP	1	0.79^**^	0.85^**^	0.81^**^	0.89^**^	-0.46^*^	-0.61^**^	-0.48^*^	-0.34^ns^	-0.68**	-0.77^**^	-0.61^**^	-0.39^ns^	-0.32^ns^	-0.2^ns^
Chla		1	0.97^**^	0.99^**^	0.95^**^	-0.02^ns^	-0.23^ns^	-0.17^ns^	0.02^ns^	-0.41^*^	-0.93^**^	-0.26^ns^	-0.05^ns^	0.02^ns^	-0.11^ns^
Chlb			1	0.98^**^	0.95^**^	-0.08^ns^	-0.26^ns^	-0.2^ns^	0.01^ns^	-0.42^*^	-0.93^**^	-0.29^ns^	-0.05^ns^	0.02^ns^	-0.16^ns^
ToChl				1	0.95^**^	-0.04^ns^	-0.24^ns^	-0.18^ns^	0.01^ns^	-0.41^*^	-0.94^**^	-0.27^ns^	-0.05^ns^	0.02^ns^	-0.13^ns^
Car					1	-0.24^ns^	-0.44^*^	-0.32^ns^	-0.19^ns^	-0.6^*^	-0.93^*^	-0.47^**^	-0.21^ns^	-0.17^ns^	-0.15^ns^
Prolin						1	0.94^**^	0.82^**^	0.91^**^	0.86^**^	0.15^ns^	0.94^**^	0.81^**^	0.88^**^	0.09^ns^
Suger							1	0.78^**^	0.93^**^	0.96^**^	0.36^ns^	0.98^**^	0.85**	0.9**	0.13^ns^
Phenol								1	0.65^**^	0.67^**^	0.18^ns^	0.84^**^	0.58^**^	0.57^**^	0.07^ns^
Protein									1	0.87^**^	0.12^ns^	0.91^**^	0.91^**^	0.98^**^	0.09^ ns^
Flav										1	0.54^**^	0.94^**^	0.8^**^	0.87**	0.15^ns^
MDA											1	0.37^ns^	0.14^ns^	0.11^ns^	0.18^ns^
SOD												1	0.84^**^	0.86^**^	0.11^ns^
APX													1	0.91^**^	0.14^ns^
GPX														1	0.13^ns^
CAT															1

	PPO	LL	LW	PH	NN	NL	SD	PD	PL	PW20	GW20	NGP	Yield
NP	-0.3^ns^	0.09^ns^	-0.16^ns^	0.44^*^	0.01^ns^	0.12^ns^	-0.25^ns^	-0.14^ns^	-0.03^ns^	0.59^**^	-0.11^ns^	-0.28^ns^	0.95^**^
Chla	-0.2^ns^	-0.2^ns^	-0.21^ns^	0.34^ns^	-0.81^**^	0.07^ns^	-0.44	0.04^ns^	-0.2^ns^	0.57^**^	-0.27^ns^	-0.2^ns^	0.7^**^
Chlb	-0.22^ns^	-0.16^ns^	-0.21^ns^	0.38^ns^	-0.12^ns^	0.12^ns^	-0.29^ns^	0.05^ns^	-0.19^ns^	0.65^**^	-0.2^ns^	-0.22^ns^	0.78^**^
ToChl	-0.21^ns^	-0.19^ns^	-0.22^ns^	0.36^ns^	-0.16^ns^	0.09^ns^	-0.39^ns^	0.05^ns^	-0.2^ns^	0.6^**^	-0.25^ns^	-0.21^ns^	0.74^**^
Car	-0.22^ns^	0.02^ns^	-0.13^ns^	0.36^ns^	-0.17^ns^	0.15^ns^	-0.41^*^	0.01^ns^	-0.1^ns^	0.61^**^	-0.26^ns^	-0.21^ns^	0.82^**^
Prolin	0.42^*^	-0.57^**^	0.08^ns^	-0.44^*^	-0.21^ns^	-0.21^ns^	-0.07^ns^	0.24^ns^	-0.08^ns^	-0.13^ns^	0.03^ns^	0.36^ns^	-0.49^*^
Suger	0.33^ns^	-0.6^**^	-0.02^ns^	-0.45^*^	-0.11^ns^	-0.28^ns^	0.16^ns^	0.17^ns^	-0.13^ns^	-0.26^ns^	0.19^ns^	0.27^ns^	-0.62^**^
Phenol	0.83^**^	-0.48^*^	0.54^**^	-0.73^**^	-0.54^**^	-0.21^ns^	0.04^ns^	0.61^**^	0.34^ns^	-0.19^ns^	0.23^ns^	0.8^**^	-0.53^**^
Protein	0.18^ns^	-0.64^**^	-0.18^ns^	-0.27^ns^	0.009^ns^	-0.28^ns^	0.04^ns^	0.08^ns^	-0.24^ns^	-0.04^ns^	0.16^ns^	0.11^ns^	-0.36^ns^
Flav	0.24^ns^	-0.52^**^	-0.07^ns^	-0.4^ns^	0.05^ns^	-0.27^ns^	0.24^ns^	0.06^ns^	-0.18^ns^	-0.35^ns^	0.22^ns^	0.19^ns^	-0.66^**^
MDA	0.09^ns^	0.08^ns^	0.06^ns^	-0.3^ns^	0.23^ns^	-0.1^ns^	0.39^ns^	-0.14^ns^	0.08^ns^	-0.57^*^	0.33^ns^	0.08^ns^	-0.66^**^
SOD	0.44^*^	-0.59^**^	0.08^ns^	-0.51^**^	-0.15^ns^	-0.27^ns^	0.12^ns^	0.23^ns^	-0.01^ns^	-0.24^ns^	0.23^ns^	0.38^ns^	-0.62^**^
APX	0.16^ns^	-0.46^*^	-0.14^ns^	-0.27^ns^	0.01^ns^	-0.21^ns^	0.09^ns^	0.08^ns^	-0.18^ns^	-0.06^ns^	0.18^ns^	0.1^ns^	-0.42^*^
GPX	0.96^**^	-0.61^**^	-0.25^ns^	-0.23^ns^	0.09^ns^	-0.25^ns^	0.05^ns^	0.004^ns^	-0.35^ns^	-0.09^ns^	0.11^ns^	0.03^ns^	-0.36^ns^
CAT	0.03^ns^	0.02^ns^	0.03^ns^	-0.2^ns^	-0.05^ns^	-0.16^ns^	-0.01^ns^	0.02^ns^	-0.45^*^	-0.55^**^	0.43^*^	0.01^ns^	-0.27^ns^
PPO	1	-0.13^ns^	0.88^**^	-0.74^**^	-0.66^**^	-0.09^ns^	-0.05^ns^	0.74^**^	0.65^**^	-0.17^ns^	0.18^ns^	0.99^**^	-0.36^ns^
LL		1	0.31^ns^	0.15^ns^	0.08^ns^	0.57^**^	-0.1^ns^	-0.08^ns^	0.24^ns^	0.15^ns^	-0.12^ns^	-0.08^ns^	0.21^ns^
LW			1	-0.62^**^	-.062^**^	0.13^ns^	-0.12^ns^	0.66^**^	0.73^**^	-0.08^ns^	0.09^ns^	0.91^**^	-0.17^ns^
PH				1	0.64^**^	0.24^ns^	0.05^ns^	-0.39^ns^	-0.31^ns^	0.41^*^	-0.41^*^	-0.73^**^	0.49^**^
NN					1	-0.02^ns^	0.16^ns^	-0.67^**^	-0.36^ns^	-0.01^ns^	-0.15^ns^	-0.66^**^	0.09^ns^
NL						1	-0.05^ns^	0.18^ns^	0.17^ns^	0.48^*^	-0.15^ns^	-0.06^ns^	0.26^ns^
SD							1	0.1^ns^	-0.03^ns^	-0.17^ns^	0.38^ns^	-0.06^ns^	-0.19^ns^
PD								1	0.46^*^	0.18^ns^	0.18^ns^	0.75^**^	-0.12^ns^
PL									1	0.21^ns^	0.05^ns^	0.68^**^	0.01^ns^
PW20										1	-0.18^ns^	-0.15^ ns^	0.71^**^
GW20											1	0.17^ ns^	-0.06^ns^
NGP												1	-0.33^ns^
Yield													1

*PH* Plant height, *NN* Number of nodes, *NL* Distance internode, *SD* Stem diameter, *PD* Pod diameter, *PL* Pod length, *PW 20* Weight 20 pods, *GW 20* Weight 20 grains, *NP* Number of pods, *LL* Leaf length, *Yi* Yield, *LW* Leaf width, *NGP* Number of seeds in pods, *Chla* Chlorophyll a, *Chlb* Chlorophyll b, *ChlT* Total chlorophyll, *Car* Carotenoid, *MDA* Malondialdehyde, *CAT* Catalase, *SOD* Superoxide dismutase, *GPX* Guaiacol peroxidase, *APX* Ascorbate peroxidase, *PPO* Polyphenol oxidase

^ns^Correlation is not significant

^*^Correlation is significant at the 0.05 level

^**^Correlation is significant at the 0.01 level

The present study found a positive and significant correlation between these two traits (the number of seeds in pods and leaf width), suggesting that these traits could be used as an indirect selection index in future breeding programs for cowpea plants under water stress. Moreover, a larger leaf length and width can lead to a higher leaf area index, potentially boosting the growth rate by increasing the presence of photosynthetic pigments and improving the photosynthesis rate [86]. In the present study, a robust and statistically significant correlation was observed between yield and leaf length, leaf width, as well as pigments content. This finding suggests that the treatment of plants with yeast extract and EBR resulted in increased dry matter accumulation, ultimately leading to improved yield. The positive effects on plant growth and productivity can be attributed to the enhanced development of leaves, characterized by increased length and width. Additionally, the optimization of pigments content likely contributed to improved photosynthetic efficiency and biomass production. These results highlight the potential of using yeast extract and EBR as effective factors for promoting crop yield enhancement in agricultural systems. Given the strong and significant correlation between seed yield and certain traits evaluated in this study, selecting based on these traits can enhance seed yield under water stress condition. In a breeding program, it is crucial to take into account both the direct and indirect effects of favorable traits on seed yield. By comprehensively analyzing both the direct and indirect effects of traits related to seed yield, breeders can make informed decisions about which characteristics to prioritize in their breeding programs. This approach ensures that breeding programs focus on developing varieties with multiple desirable traits that collectively enhance seed yield potential [87–89].

#### Cluster analysis

**Fig. 2:**
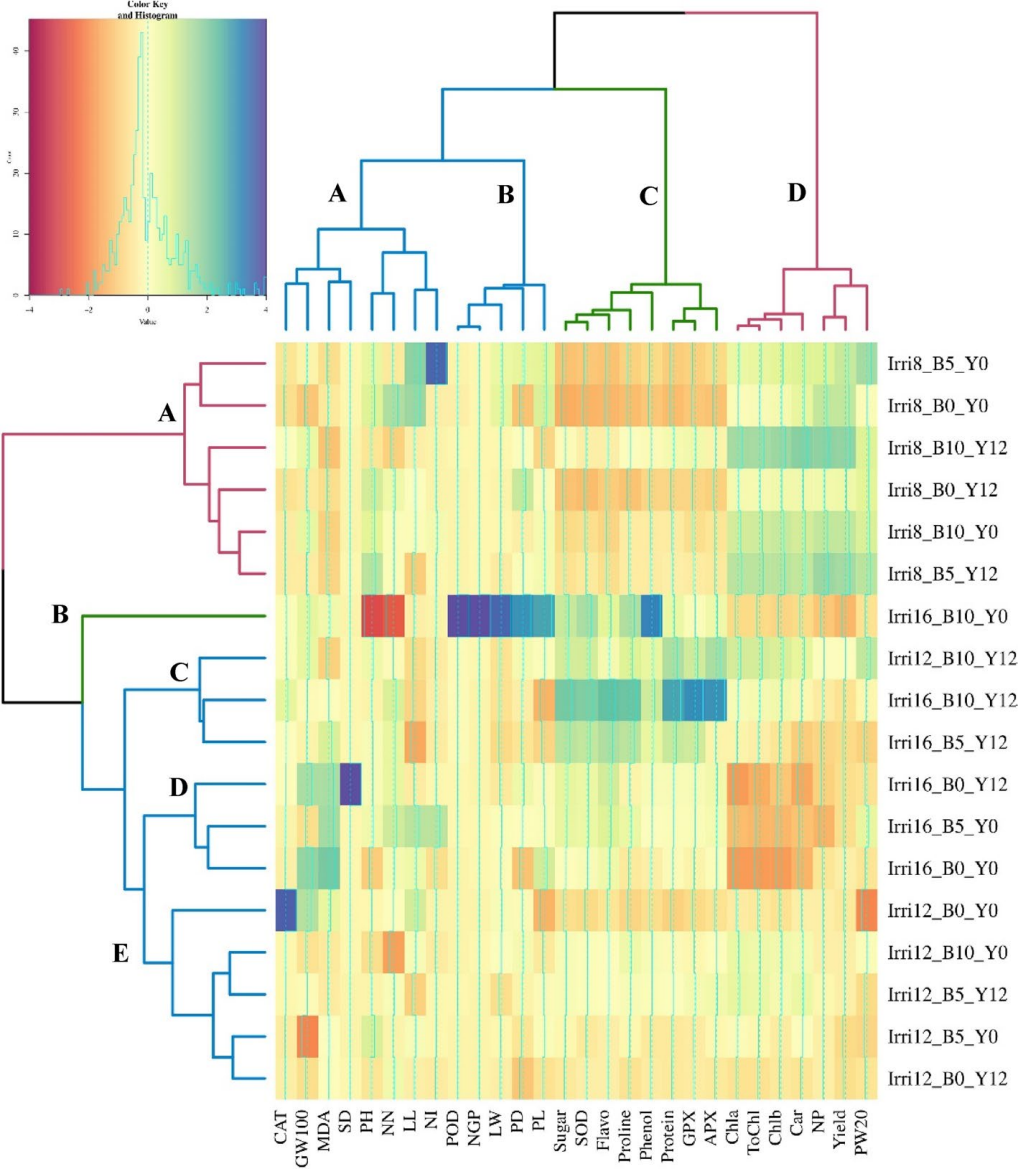
Heatmap plot obtained by cluster analysis of treatments based on the studied traits using ward method

The cluster analysis of the treatments and traits investigated in this study provided valuable insights. A two-dimensional heatmap was created, which revealed that the traits and the treatments were classified into four and five main groups, respectively. Notably, all the normal irrigation treatment (8 days) was grouped together, while the 12 and 16 day treatments, which represent drought stress conditions, were placed in separate clusters. The heatmap analysis revealed that certain physiological traits, such as total sugar content, flavonoids, proline and phenol content, total protein, SOD, APX, and GPX activities, were grouped together in group 3, while other groups consisted of a combination of physiological and morphological traits. The yield, weight of 20 pods, number of pods per plant, chlorophylls, and carotenoids content were grouped together in one group and exhibited the same response to treatments, which was consistent with the results of the trait correlation analysis. Group 1 comprised traits such as plant height, number of nodes, distance internode, stem diameter, weight 20 grains, leaf length, MDA, and CAT activity. This group confirmed the correlation and the same response of these traits to the evaluated treatments (Fig. 2). Group 4 comprised traits such as yield, number of pods, chlorophyll a, chlorophyll b, total chlorophyll, and carotenoid. These results highlight the importance of considering traits such as chlorophylls and carotenoids content, and the number of pods per plant in breeding programs aimed at developing drought-resistant cultivars with high yield. Previous research has also demonstrated a positive and significant correlation between seed yield and pigments content and pod weight [82, 83].

## Conclusion

The microbial-based biostimulants utilization has gained recognition as a cost-effective, time-saving, and environmentally friendly approach to enhance plant tolerance in challenging environmental conditions. Building upon this notion, our investigations have shed light on the promising roles of yeast extract and EBR in managing water deficit stress. These findings suggest that these stimulants hold great potential as effective candidates for developing tailored formulations aimed at improving water deficit stress tolerance in major crops such as cowpea. Nonetheless, prior to widespread implementation, it is imperative to conduct comprehensive field studies to assess the efficacy of these stimulants as biofertilizers in water deficit-prone regions.

## Methods

### Experimental design and application of treatments

The research site for this study is situated at coordinates 36° 29' North latitude and 55° East longitude. The research site is situated at an average elevation of 1366 m above sea level and experiences a climate characterized by cold and arid conditions (Shahrood city, Semnan province, Iran). The studied plant was obtained from the Ministry of Agriculture Jihad in Tehran, Iran. The collection of plant material was conducted in accordance with the supervision and permission granted by the Ministry of Agriculture Jihad of Tehran, Iran, while adhering to both national and local guidelines. All authors of the study fully complied with the prescribed local and national guidelines. Prior to field preparation, soil samples were collected from a depth of 0–30 cm in order to assess the physical and chemical properties of the soil. The experiment was conducted using a randomized complete block design with a factorial split-plot arrangement, replicated three times. The main plots consisted of three levels of the irrigation period (every 8, 12, and 16 days), while the sub-plots included different concentrations of EBR (0, 5, and 10 µm) and yeast extract (0 and 12 g/l). To prepare the treatment solution, the EBR was dissolved in 1 ml of ethanol and subsequently diluted in distilled water to achieve the desired concentrations (0, 5, and 10 µm). In each plot, five planting lines measuring five meters long were designated for each treatment. To mitigate the potential edge effect, only the middle two rows were selected for sampling purposes. EBR and yeast extract were sprayed at the 5-leaf stage post-seeding (*Vigna unguiculata*. L), and a second round of spraying was conducted 24 h later to ensure their effectiveness. A uniform quantity of 100 ml of EBR and yeast extract was evenly applied to every row, ensuring complete coverage of the plant's surface with moisture. The irrigation treatments were initiated following the second spraying, and physiological and morphological traits, such as yield and yield components, were measured during the flowering and complete seed ripening stages, respectively. The leaf samples were initially flash-frozen in liquid nitrogen and subsequently stored at a temperature of -80 °C.

### The evaluation of yield and yield components traits

Twenty plants were chosen from the three middle rows, excluding the first and last 50 cm of each row. Various traits, including plant height, leaf length and width, number of nodes, internodal distance, stem diameter, pod length and diameter, number of seeds in pods, number of pods per plant, weight of 20 pods, weight of 20 seeds, and plant yield, were measured. To accurately assess the morphological traits, yield, and yield components, an exact electronic balance with 0.001 g sensitivity and a digital caliper were utilized.

### The evaluation of physiological traits

#### Chlorophylls and carotenoids content

To determine the contents of chlorophylls and carotenoid, the flag leaf samples (0.2 g) were homogenized in 5 ml of 85% acetone. Subsequently, the homogenate was centrifuged at 3500 rpm and 4 °C for 15 min. The resulting supernatant was then adjusted to a volume of 10 ml with 85% acetone. The absorbance of the supernatant was measured at wavelengths of 480 nm, 649 nm, and 665 nm using a spectrophotometer (UV-1800; Shimadzu Corporation, Kyoto, Japan). The contents of chlorophylls and carotenoid were calculated using standard formulas [90].

#### Lipid peroxidation assay

To determine the content of MDA, 0.25 g of fresh flag leaf were homogenized in 2 ml of extraction buffer containing 1% (w/v) trichloroacetic acid (TCA). The homogenate was then centrifuged at 3500 rpm and 4 °C for 15 min. Next, 1 ml of the resulting supernatant was mixed with 2 ml of 5% (w/v) thiobarbituric acid in 20% (w/v) TCA. The mixture was incubated in boiling water for 30 min, followed by immediate cooling in an ice bath to stop the reaction. Afterward, the samples were centrifuged at 3500 rpm for 15 min, and the absorbance of the supernatant was measured at 532 nm using a spectrophotometer [91, 92].

#### Proline content

In order to prepare the samples for proline content analysis, 0.5 g of flag leaf tissue was homogenized in 4 ml of 3% sulfosalicylic acid and then filtered using Whatman filter paper. Afterwards, 2 ml of the resulting extract was mixed with 2 ml of ninhydrin reagent and 2 ml of pure acetic acid. The samples were subsequently incubated at a temperature of 90 °C for duration of one hour in a hot bath. The reaction was terminated by immediately transferring the samples to an ice container. 4 ml of toluene were introduced into the sample tubes, and the mixture was vigorously agitated for 30 s until it separated into two phases, with a colored toluene phase containing proline on top and a clear blue phase at the bottom. After 20 min, the optical density of the supernatant was measured at 520 nm using a spectrophotometer [93].

#### Soluble protein

To extract soluble protein, 1 g of leaf tissue was homogenized in 5 ml of extraction buffer (Tris-hydrochloric acid, pH = 7.5). The homogenized mixture was then subjected to centrifugation at 3500 rpm and 4 °C for duration of 15 min. The resulting supernatant obtained from the centrifugation step was utilized for assessing the activity of antioxidant enzymes. In order to establish a standard curve, a series of Bovine Serum Albumin (BSA) samples were employed. To each sample, 3 ml of Bradford solution was added and mixed with 50 μl of the extract. The resulting mixture was thoroughly vortexed to ensure proper mixing. After a 20-min incubation period, the protein concentration of each sample was determined using a spectrophotometer at a wavelength of 595 nm [94].

#### Catalase activity

To measure CAT activity, a spectrophotometer was used at a wavelength of 240 nm. The reaction mixture consisted of 3 ml of 50 mM potassium phosphate buffer, 10 μl of 30% H_2_O_2_, and 50 μl of protein extract. Aebi's method [95] was employed to monitor the catalase activity over a period of five minutes, with readings taken at 20-s intervals. The degradation of H_2_O_2_ was quantified by measuring the decrease in absorbance at 240 nm using a spectrophotometer for duration of one min.

#### Ascorbate peroxidase activity

To evaluate APX activity, the reaction mixture consisting of 600 μl of 0.1 mM EDTA, 1500 μl of 50 mM potassium phosphate buffer, 400 μl of 0.5 mM ascorbic acid, 10 μl of 30% H_2_O_2_, and 50 μl of protein extract was observed at a wavelength of 290 nm. The enzyme activity was monitored every 20 s for a period of two minutes [96].

#### Guaiacol peroxidase activity

In order to measure the activity of GPX, a reaction mixture was created by mixing 3000 μl of 50 mM potassium phosphate buffer, 10 μl of 30% H_2_O_2_, 5 μl of 200 mM guaiacol, and 50 μl of protein extract. The change in absorbance at 470 nm was observed using a spectrophotometer for duration of one minute [97].

#### Activity of superoxide dismutase

To determine the activity of APX, the reaction mixture was monitored at a wavelength of 290 nm. The mixture consisted of 600 μl of 0.1 mM EDTA, 1500 μl of 50 mM potassium phosphate buffer, 400 μl of 0.5 mM ascorbic acid, 10 μl of 30% hydrogen peroxide, and 50 μl of protein extract. Following Zhang et al.’s method [98], the enzyme activity was recorded at intervals of every 20 s for duration of two minutes.

#### Polyphenol oxidase activity

The activity of PPO enzyme was evaluated using an extracted enzyme extract. To create the reaction mixture, 1.5 ml of 50 mM potassium phosphate buffer with a pH of 7.6, 0.4 ml of 0.02 M pyrogallol, and 100 μl of the enzyme extract were combined. The mixture was then incubated at a temperature of 30 °C for duration of three minutes. Following the incubation, changes in absorbance were measured at a wavelength of 430 nm [99].

#### Total flavonoid

The quantity of flavonoid was determined following the method described by Wu et al. (2006) [100]. A mixture of 0.15 ml of methanol extract of the leaf, 1.25 ml of distilled water, and 10 μl of 5% sodium nitrate was prepared in a 2 ml tube. Then, 0.15 ml of 10% aluminum chloride and 0.5 ml of 1 M sodium hydroxide were added to the solution. The solution was immediately subjected to spectrophotometric analysis at 510 nm to measure the absorbance. A calibration curve was drawn using Routine (Sigma Company) as the standard.

#### Measurement of total phenol content

To determine the total phenol content of the methanol extract of leaves, the spectrophotometric method developed by Ciocalteu and Folin [101] was employed. In a 2 ml tube, a mixture consisting of 0.1 ml of the methanolic extract, 1.5 ml of distilled water, and 0.1 ml of Folin-Ciocalteu (2 N) (Merck, Germany) was prepared. Following a 10-min incubation period, 0.3 ml of 5% sodium carbonate was added to the solution, and the samples were further incubated at room temperature for 90 min [101]. The absorbance of the solution was then measured at a wavelength of 760 nm using a spectrophotometer.

#### Total sugar content

To extract the sample, the powdered leaf tissue was combined with 1.5 ml of 80% ethanol. The mixture was then vortexed for 10 min and centrifuged at 3000 rpm for 15 min at a temperature of 4 °C. The resulting supernatants were collected and kept at 50 °C to eliminate any residual alcohol. The dried samples were subsequently treated with 10 ml of distilled water, 0.5 ml of normal barium hydroxide, and 0.5 ml of 5% zinc sulfate before undergoing a second round of centrifugation. From the resulting supernatant, 2 ml was mixed with 1 ml of 5% phenol and 5 ml of 98% sulfuric acid. The solutions were incubated at room temperature for 45 min, and the absorbance at 485 nm was measured using a spectrophotometer [102].

### Supplementary Information

**Additional file 1:** **Table S1**. Variance analysis of evaluated traits of Cowpea under different levels of water deficit, 24-epibrassinolide, and yeast extract in two different year crops. **Table S2.** The mean comparison of simple effect of year on evaluated traits of Cowpea. **Table S3.** The mean comparison of simple effect of different levels of water deficit on evaluated traits of Cowpea. **Table S4.** The mean comparison of simple effect of different 24-epibrassinolide levels on evaluated traits of Cowpea. **Table S5.** The mean comparison of simple effect of different yeast extract levels on evaluated traits of Cowpea.**Table S6.** The mean comparison of interaction effects of different levels of water deficit and year crops on evaluated traits of Cowpea. **Table S7.** The mean comparison of interaction effects of different yeast extract levels and 24-epibrassinolide on evaluated traits of Cowpea. **Table S8.** The mean comparison of interaction effects of different year crops and 24-epibrassinolide (B) on evaluated traits of Cowpea. **Table S9.** The mean comparison of interaction effects of different year crops and yeast extract on evaluated traits of Cowpea.**Table S10.** The mean comparison of interaction effects of different levels of water deficit and 24-epibrassinolide (B) on evaluated traits of Cowpea. **Table S11.** The mean comparison of interaction effects of different levels of water deficit and yeast extract on evaluated traits of Cowpea. **Table S12.** The mean comparison of interaction effects of **d**ifferent levels of water deficit, year and 24-epibrassinolide (B) on evaluated traits of Cowpea. **Table S13.** The mean comparison of interaction effects of **d**ifferent levels of water deficit, year and yeast extract (Y) on evaluated traits of Cowpea. **Table S14.** The mean comparison of interaction effects of year, 24-epibrassinolide (B) and yeast extract (Y) on evaluated traits of Cowpea. **Table S15.** The mean comparison of interaction effects of different levels of water deficit, 24-epibrassinolide (B) and yeast extract (Y) on evaluated traits of Cowpea. **Table S16.** The mean comparison of interaction effects of different levels of water deficit, 24-epibrassinolide (B), yeast extract (Y) and year on evaluated traits of Cowpea.

## Data Availability

The data generated or analyzed in this study are included in this article. Other materials that support the findings of this study are available from the corresponding author on reasonable request.
