# Supplementary material for: Assessing the effects of 24-epibrassinolide and yeast extract at various levels on cowpea’s morphophysiological and biochemical responses under water deficit stress
Source: BMC Plant Biol. 2023 Nov 27;23:593. doi: 10.1186/s12870-023-04548-6 (PMC10680335; doi:10.1186/s12870-023-04548-6)
Supplement: Supplementary file 1 — Additional file 1: Table S1. Variance analysis of evaluated traits of Cowpea under different levels of water deficit, 24-epibrassinolide, and yeast extract in two different year crops. Table S2. The mean comparison of simple effect of year on evaluated traits of Cowpea. Table S3. The mean comparison of simple effect of different levels of water deficit on evaluated traits of Cowpea. Table S4. The mean comparison of simple effect of different 24-epibrassinolide levels on evaluated traits of Cowpea. Table S5. The mean comparison of simple effect of different yeast extract levels on evaluated traits of Cowpea.Table S6. The mean comparison of interaction effects of different levels of water deficit and year crops on evaluated traits of Cowpea. Table S7. The mean comparison of interaction effects of different yeast extract levels and 24-epibrassinolide on evaluated traits of Cowpea. Table S8. The mean comparison of interaction effects of different year crops and 24-epibrassinolide (B) on evaluated traits of Cowpea. Table S9. The mean comparison of interaction effects of different year crops and yeast extract on evaluated traits of Cowpea.Table S10. The mean comparison of interaction effects of different levels of water deficit and 24-epibrassinolide (B) on evaluated traits of Cowpea. Table S11. The mean comparison of interaction effects of different levels of water deficit and yeast extract on evaluated traits of Cowpea. Table S12. The mean comparison of interaction effects of different levels of water deficit, year and 24-epibrassinolide (B) on evaluated traits of Cowpea. Table S13. The mean comparison of interaction effects of different levels of water deficit, year and yeast extract (Y) on evaluated traits of Cowpea. Table S14. The mean comparison of interaction effects of year, 24-epibrassinolide (B) and yeast extract (Y) on evaluated traits of Cowpea. Table S15. The mean comparison of interaction effects of different levels of water deficit, 24-epibrassinolide (B) a [file 12870_2023_4548_MOESM1_ESM.docx]

**Table S1**. Variance analysis of evaluated traits of Cowpea under different levels of water deficit, 24-epibrassinolide, and yeast extract in two different year crops.

| **SOV** | **df** | **PH** | **NN** | **NL** | **SD** | **PD** | **PL** | **PW20** | **GW100** | **NP** | **LL** | **Yi** |
| --- | --- | --- | --- | --- | --- | --- | --- | --- | --- | --- | --- | --- |
| **Year** | 1 | 2578.59^ns^ | 18.55^*^ | 137.35^ns^ | 3985.07 ^ns^ | 0.13 ^ns^ | 1.32 ^ns^ | 3108.34^**^ | 227.32^*^ | 0.34^*^ | 13.44^ns^ | 3537597.61^*^ |
| **Year*Rep** | 4 | 85.5^**^ | 1.11 ^ns^ | 4.4 ^ns^ | 4362.91 ^ns^ | 0.36 ^ns^ | 5.2 ^ns^ | 32.57 ^ns^ | 17.9^**^ | 0.02^ns^ | 0.51^ns^ | 259299.18^*^ |
| **Irii** | 2 | 39.84 ^ns^ | 0.35 ^ns^ | 7.3 ^ns^ | 4456.5 ^ns^ | 0.12 ^ns^ | 1.85 ^ns^ | 168.24^ns^ | 2.59 ^ns^ | 137.99^**^ | 1.65^*^ | 4226685.72^**^ |
| **Year*Irri** | 2 | 38.52 ^ns^ | 0.4 ^ns^ | 12.62 ^ns^ | 4531.7 ^ns^ | 0.22 ^ns^ | 3.37 ^ns^ | 28.25 ^ns^ | 14.11^*^ | 0.009^ns^ | 1.26 ^ns^ | 251235.76^ns^ |
| **Year*Irri*Rep** | 8 | 51.08 ^*^ | 2.3 ^ns^ | 8.61 ^ns^ | 4438.15 ^ns^ | 0.74 ^ns^ | 3.16 ^ns^ | 43.83^*^ | 2.99 ^ns^ | 0.82* | 0.34 ^ns^ | 90382.48 ^ns^ |
| **B** | 2 | 31.93 ^ns^ | 2.95 ^ns^ | 13.5 ^ns^ | 4380.4 ^ns^ | 0.48 ^ns^ | 0.07 ^ns^ | 157 ^**^ | 25.08 ^**^ | 1.62^*^ | 2.01^*^ | 75294.54 ^ns^ |
| **Y** | 1 | 6.35 ^ns^ | 1.21 ^ns^ | 13.68 ^ns^ | 4286.58 ^ns^ | 0.02 ^ns^ | 7.92 ^ns^ | 200.25 ^**^ | 23.08 ^**^ | 2.52^*^ | 21.76^**^ | 16.774.45 ^ns^ |
| **B*Y** | 2 | 7.41 ^ns^ | 1.11 ^ns^ | 12.55 ^ns^ | 4121.95 ^ns^ | 0.51 ^ns^ | 1.18 ^ns^ | 131.9 ^*^ | 15.79 ^*^ | 4.84^**^ | 2.66^**^ | 310667.87^*^ |
| **Year*B** | 2 | 39.04 ^ns^ | 0.6 ^ns^ | 2.79 ^ns^ | 4218.21 ^ns^ | 0.3 ^ns^ | 0.68 ^ns^ | 83.93^*^ | 0.12 ^ns^ | 0.58^ns^ | 1.68^*^ | 174982.78 ^ns^ |
| **Year*Y** | 1 | 16.54 ^ns^ | 2.09 ^ns^ | 2.59 ^ns^ | 4432.69 ^ns^ | 1 ^ns^ | 1.52 ^ns^ | 96.78^*^ | 0.05 ^ns^ | 0.46^ns^ | 2.36^*^ | 808.97 ^ns^ |
| **Irri*B** | 4 | 7.18 ^ns^ | 0.44 ^ns^ | 3.47 ^ns^ | 4355.13 ^ns^ | 0.25 ^ns^ | 1.86 ^ns^ | 35.97 ^ns^ | 3.33 ^ns^ | 1.22^*^ | 0.18 ^ns^ | 159722.5 ^ns^ |
| **Irri*Y** | 2 | 19.38 ^ns^ | 1.46 ^ns^ | 5.02 ^ns^ | 4457.61 ^ns^ | 0.26 ^ns^ | 5.84 ^ns^ | 28.73 ^ns^ | 0.34 ^ns^ | 0.01^ns^ | 0.18 ^ns^ | 15136.13 ^ns^ |
| **Year*Irri*B** | 4 | 1.58 ^ns^ | 1.18 ^ns^ | 8.74 ^ns^ | 4371.02 ^ns^ | 0.46 ^ns^ | 5.9 ^ns^ | 78.9^**^ | 5.34 ^ns^ | 0.31^ns^ | 0.39 ^ns^ | 95414.06 ^ns^ |
| **Year*Irri*Y** | 2 | 14.68 ^ns^ | 0.84 ^ns^ | 10.32 ^ns^ | 4429.12 ^ns^ | 0.11 ^ns^ | 2.49 ^ns^ | 71.45^*^ | 2.14 ^ns^ | 0.74 ^ns^ | 0.29 ^ns^ | 43661.22 ^ns^ |
| **Year*B*Y** | 2 | 16.69 ^ns^ | 0.99 ^ns^ | 2.57 ^ns^ | 4280.74 ^ns^ | 0.32 ^ns^ | 2.14 ^ns^ | 34.14 ^ns^ | 7.86 ^ns^ | 0.08^ns^ | 0.88 ^ns^ | 86374.13 ^ns^ |
| **Irri*B*Y** | 4 | 17.82 ^ns^ | 1.32 ^ns^ | 4.25 ^ns^ | 4302.55 ^ns^ | 0.57 ^ns^ | 1.54 ^ns^ | 4.94 ^ns^ | 2.51 ^ns^ | 0.74 ^ns^ | 0.62 ^ns^ | 46832.91 ^ns^ |
| **Year*Irri*B*Y** | 4 | 11.03 ^ns^ | 0.73 ^ns^ | 6.83 ^ns^ | 4398.91 ^ns^ | 0.86 ^ns^ | 6.87 ^ns^ | 16.01 ^ns^ | 0.54 ^ns^ | 0.18 ^ns^ | 0.25 ^ns^ | 4595.45 ^ns^ |
| **Error** | 59 | 20.01 | 1.2 | 7.74 | 4554.37 | 0.45 | 2.51 | 18.47 | 3.95 | 0.38 | 0.51 | 71071.69 |

**Irri:** Different levels of water deficit**, B:** Different concetrations of 24-epibrassinolide**, Y:** Different concetrations of yeast extract, **PH:** Plant height, **NN:** Number of nodes, **NL:** Distance internode, **SD:** Stem diameter, **PD:** PPO diameter, **PL:** PPO length, **PW 20:** Weight 20 PPOs, **GW 100:** Weight 100 grains, **NP:** Number of PPOs , **LL:** Leaf length, **Yi:** Yield**.**

* and ** Significantly at the probability level of %5 and %1, respectively.

| **SOV** | **df** | **LW** | **NGP** | **Chla** | **Chlb** | **ChlT** | **Car** | **Prolin** | **Sugar** | **Phenol** | **Protein** |
| --- | --- | --- | --- | --- | --- | --- | --- | --- | --- | --- | --- |
| **Year** | 1 | 25.05 ns | 12991.81 ns | 0.29^ns^ | 0.02 ^ns^ | 0.48^ns^ | 0.65 ^ns^ | 0.07 ^ns^ | 0.04 ^ns^ | 427.19 ^ns^ | 309.01^ns^ |
| **Year*Rep** | 4 | 8.06 ns | 13881.98 ns | 1.42^ns^ | 0.2 ^ns^ | 0.94 ^ns^ | 0.34 ^ns^ | 0.1 ^ns^ | 0.007 ^ns^ | 428.33 ^ns^ | 675.71 ^ns^ |
| **Irii** | 2 | 5.28 ns | 13439.83 ns | 222.01^**^ | 73.56^**^ | 547.28^**^ | 295.55^**^ | 32.24^**^ | 64.04^**^ | 3974.99^ns^ | 64412.4^**^ |
| **Year*Irri** | 2 | 3.21 ns | 13605.9 ns | 0.06 ^ns^ | 0.04 ^ns^ | 0.21 ^ns^ | 0.43 ^ns^ | 0.02 ^ns^ | 0.008 ^ns^ | 429.02 ^ns^ | 414.4 ^ns^ |
| **Year*Irri*Rep** | 8 | 6.77 ns | 13679.28 ns | 0.46^ns^ | 0.53^*^ | 0.21 ^ns^ | 0.76 ^ns^ | 0.16^*^ | 0.06 ^ns^ | 408.16 ^ns^ | 615.8 ^ns^ |
| **B** | 2 | 5.91 ns | 13430.61 ns | 119.79^**^ | 29.36^**^ | 256.42^**^ | 65.69^**^ | 34.04^**^ | 22.85^**^ | 3405.42^**^ | 59536.73^**^ |
| **Y** | 1 | 57.22 ns | 13815.55 ns | 54.98^**^ | 24.08^**^ | 151.86^**^ | 14.66^**^ | 8.04^**^ | 20.24^**^ | 145.27 ^ns^ | 76604.37^**^ |
| **B*Y** | 2 | 5.44 ns | 13418.37 ns | 16.28^**^ | 1.44^**^ | 26.97^**^ | 14.08^**^ | 0.63^**^ | 0.82^**^ | 541.77 ^ns^ | 10944.62^**^ |
| **Year*B** | 2 | 5.62 ns | 13503.73 ns | 0.25 ^ns^ | 0.03 ^ns^ | 0.39 ^ns^ | 0.01 ^ns^ | 0.005 ^ns^ | 0.006 ^ns^ | 388.38 ^ns^ | 231.66 ^ns^ |
| **Year*Y** | 1 | 23.71 ns | 13545.51 ns | 0.53 ^ns^ | 0.005 ^ns^ | 0.43 ^ns^ | 0.08 ^ns^ | 0.006 ^ns^ | 0.0002 ^ns^ | 442 ^ns^ | 624.21 ^ns^ |
| **Irri*B** | 4 | 10.37 ns | 13424.9 ns | 0.73 ^ns^ | 0.25 ^ns^ | 1.25 ^ns^ | 0.99 ^ns^ | 1.85 ^**^ | 0.24^**^ | 507.26 ^ns^ | 910.6 ^ns^ |
| **Irri*Y** | 2 | 8.97 ns | 13789.34 ns | 2.25 ^ns^ | 1.55^**^ | 6.85^**^ | 0.36 ^ns^ | 0.27^*^ | 0.52^**^ | 335.83 ^ns^ | 2698.37^*^ |
| **Year*Irri*B** | 4 | 8.32 ns | 13761.01 ns | 0.7^ns^ | 0.07 ^ns^ | 0.1 ^ns^ | 0.07 ^ns^ | 0.008 ^ns^ | 0.002 ^ns^ | 430.65 ^ns^ | 506.37 ^ns^ |
| **Year*Irri*Y** | 2 | 11.4 ns | 13371.77 ns | 0.41 ^ns^ | 0.42 ^ns^ | 1.16 ^ns^ | 0.05 ^ns^ | 0.02 ^ns^ | 0.005 ^ns^ | 408.5 ^ns^ | 633.49 ^ns^ |
| **Year*B*Y** | 2 | 8.89 ns | 13660.57 ns | 0.41^ns^ | 0.08 ^ns^ | 0.18 ^ns^ | 0.03 ^ns^ | 0.01 ^ns^ | 0.01 ^ns^ | 464.15 ^ns^ | 926.68 ^ns^ |
| **Irri*B*Y** | 4 | 11.58 ns | 13534.39 ns | 0.36 ^ns^ | 0.2 ^ns^ | 0.7 ^ns^ | 0.94 ^ns^ | 0.08 ^ns^ | 0.02 ^ns^ | 526.26 ^ns^ | 2037.44^**^ |
| **Year*Irri*B*Y** | 4 | 7.29 ns | 13803.52 ns | 0.1 ^ns^ | 0.09 ^ns^ | 0.1 ^ns^ | 0.11 ^ns^ | 0.01 ^ns^ | 0.007 ^ns^ | 465.43 ^ns^ | 632.96 ^ns^ |
| **Error** | 59 | 9.65 | 13866.16 | 0.72 | 0.24 | 0.94 | 0.61 | 0.07 | 0.03 | 442.34 | 556.03 |

**Table S1** (continued).

**Irri:** Different levels of water deficit**, B:** Different concetrations of 24-epibrassinolide**, Y:** Different concetrations of yeast extract, **LW:** Leaf width, **NGP:** Number of seeds in PPO, **Chla:** Chlorophyll a, **Chlb:** Chlorophyll b, **ChlT:** Total chlorophyll, **Car:** Carotenoid.

* and ** Significantly at the probability level of %5 and %1, respectively.

**Table S1** (continued).

| **SOV** | **df** | **Flavonoid** | **MDA** | **SOD** | **APX** | **GPX** | **CAT** | **PPO** |
| --- | --- | --- | --- | --- | --- | --- | --- | --- |
| **Year** | 1 | 0.13 ^ns^ | 3.99 ^ns^ | 0.00003^ns^ | 0.0008^ns^ | 3.39^ns^ | 0.003^**^ | 0.325 ^ns^ |
| **Year*Rep** | 4 | 11.37^*^ | 0.56 ^ns^ | 0.00001^ns^ | 0.00002^*^ | 0.00001 ^ns^ | 0.000005 ^ns^ | 0.327 ^ns^ |
| **Irii** | 2 | 1783.74^**^ | 224.23^**^ | 0.004^**^ | 0.01^**^ | 0.01^**^ | 0.004^**^ | 0.391 ^ns^ |
| **Year*Irri** | 2 | 3.18 ^ns^ | 0.5 ^ns^ | 0.000004^ns^ | 0.001^**^ | 0.00002 ^ns^ | 0.004^**^ | 0.323 ^ns^ |
| **Year*Irri*Rep** | 8 | 4.61 ^ns^ | 1.58 ^ns^ | 0.00002^ns^ | 0.00001^ns^ | 0.00001 ^ns^ | 0.000008 ^ns^ | 0.329 ^ns^ |
| **B** | 2 | 185.71^**^ | 61.76^**^ | 0.001^**^ | 0.01^**^ | 0.008^**^ | 0.001^**^ | 0.384 ^ns^ |
| **Y** | 1 | 385.73^**^ | 30.91^**^ | 0.009^**^ | 0.009^**^ | 0.01^**^ | 0.0004^**^ | 0.234 ^ns^ |
| **B*Y** | 2 | 19.83^**^ | 0.16 ^ns^ | 0.00003^ns^ | 0.005^**^ | 0.003^**^ | 0.004^**^ | 0.307 ^ns^ |
| **Year*B** | 2 | 2.12 ^ns^ | 1.28 ^ns^ | 0.00002^**^ | 0.001^**^ | 0.00001 ^ns^ | 0.003^**^ | 0.325 ^ns^ |
| **Year*Y** | 1 | 4.48 ^ns^ | 0.27 ^ns^ | 0.00001^**^ | 0.001^**^ | 0.00002 ^ns^ | 0.003^**^ | 0.32 ^ns^ |
| **Irri*B** | 4 | 3.87 ^ns^ | 7.29^**^ | 0.00006^ns^ | 0.001^**^ | 0.0004^**^ | 0.004^**^ | 0.327 ^ns^ |
| **Irri*Y** | 2 | 42.9^**^ | 1.46 ^ns^ | 0.00001^ns^ | 0.0006^**^ | 0.001^**^ | 0.004^**^ | 0.316 ^ns^ |
| **Year*Irri*B** | 4 | 2.7 ^ns^ | 0.87 ^ns^ | 0.000007^ns^ | 0.001^**^ | 0.00001 ^ns^ | 0.003^**^ | 0.325 ^ns^ |
| **Year*Irri*Y** | 2 | 4.02 ^ns^ | 0.76 ^ns^ | 0.00001^ns^ | 0.0009^**^ | 0.00001 ^ns^ | 0.003^**^ | 0.327 ^ns^ |
| **Year*B*Y** | 2 | 2.79 ^ns^ | 0.88 ^ns^ | 0.00001^ns^ | 0.001^**^ | 0.00001 ^ns^ | 0.003^**^ | 0.326 ^ns^ |
| **Irri*B*Y** | 4 | 11.26^*^ | 0.15 ^ns^ | 0.000009^ns^ | 0.001^**^ | 0.0005^**^ | 0.003^**^ | 0.325 ^ns^ |
| **Year*Irri*B*Y** | 4 | 3.34 ^ns^ | 0.58 ^ns^ | 0.00001^ns^ | 0.001^**^ | 0.00002 ^ns^ | 0.003^**^ | 0.326 ^ns^ |
| **Error** | 59 | 3.6 | 1.59 | 0.00001 | 0.000009 | 0.00002 | 0.000006 | 0.332 |

**Irri:** Different levels of water deficit**, B:** Different concetrations of 24-epibrassinolide**, Y:** Different concetrations of yeast extract, **MDA**: Malondialdehyde, **CAT:** Catalase, **SOD:** Superoxide dismutase, **GPX:** Guaiacol peroxidase, **APX:** Ascorbate peroxidase, **PPO:** Polyphenol oxidase.

* and ** Significantly at the probability level of %5 and %1, respectively.

**Table S2.** The mean comparison of simple effect of year on evaluated traits of Cowpea.

| **Treatment** | **PH** | **NN** | **NL** | **SD** | **PD** | **PL** | **PW20** | **GW100** | **NP** | **LL** | **Yield** |
| --- | --- | --- | --- | --- | --- | --- | --- | --- | --- | --- | --- |
| **Year** |  |  |  |  |  |  |  |  |  |  |  |
| **1** | 21.08±2.19 b | 10.03±0.63 b | 1.14±0.41 b | 9.93±0.35 a | 7.03±0.62 a | 11.76±1.35 a | 35.89±1.81 a | 15.8±1.46 a | 8.03±1.01 b | 6.02±0.72 b | 1758.04±60.78 a |
| **2** | 30.92±3.55 a | 11.46±0.43 a | 3.24±0.6 a | 8.42±0.74 a | 6.96±0.73 a | 11.99±1.09 a | 22.15±1.94 b | 12.86±1.56 b | 9.95±0.35 a | 7±0.06 a | 1387.2±60.39 b |

**Table S2** (continued).

| **Treatment** | **LW** | **NGP** | **Chla** | **Chlb** | **Total Chl** | **Car** | **Prolin** | **Suger** | **Phenol** | **Protein** |
| --- | --- | --- | --- | --- | --- | --- | --- | --- | --- | --- |
| **Year** |  |  |  |  |  |  |  |  |  |  |
| **1** | 4.2±0.55 a | 8.9±1.71 a | 10.49±2.93 a | 4.9±1.59 a | 15.39±1.39 a | 11.01±2.77 a | 3.67±1.19 a | 4.98±1.39 a | 33.75±1.96 a | 198.3±60.16 a |
| **2** | 5.16±0.93 a | 9±0.28 a | 10.38±2.77 a | 4.9±1.57 a | 15.29±1.24 a | 11.17±2.87 a | 3.7±1.25 a | 4.98±1.37 a | 33.75±2.31 a | 193.9±65.86 a |

**Table S2** (continued).

| **Treatment** | **Flavonoid** | **MDA** | **SOD** | **APX** | **GPX** | **CAT** | **PPO** | **LW** | **NGP** |
| --- | --- | --- | --- | --- | --- | --- | --- | --- | --- |
| **Year** |  |  |  |  |  |  |  |  |  |
| **1** | 20.05±1.73 a | 8.15±2.55 a | 0.046±0.001 a | 0.08±0.003 a | 0.06±0.002 a | 0.04±0.004 a | 0.144±0.01 a | 4.2±0.55 a | 8.9±1.71 a |
| **2** | 19.85±1.91 a | 7.71±2.83 a | 0.047±0.001 a | 0.07±0.003 b | 0.061±0.002 a | 0.03±0.009 b | 0.15±0.081 a | 5.06±0.3 a | 9±1.28 a |

**Irri:** Different levels of water deficit, **PH:** Plant height, **NN:** Number of nodes, **NL:** Distance internode, **SD:** Stem diameter, **PD:** PPO diameter, **PL:** PPO length, **PW 20:** Weight 20 PPOs, **GW 100:** Weight 100 grains, **NP:** Number of PPOs , **LL:** Leaf length , **Yi:** Yield, **LW:** Leaf width, **NGP:** Number of seeds in PPO, **Chla:** Chlorophyll a, **Chlb:** Chlorophyll b, **ChlT:** Total chlorophyll, **Car:** Carotenoid, , **MDA**: Malondialdehyde, **CAT:** Catalase, **SOD:** Superoxide dismutase, **GPX:** Guaiacol peroxidase, **APX:** Ascorbate peroxidase, **PPO:** Polyphenol oxidase.

* and ** Significantly at the probability level of %5 and %1, respectively.*Mean comparison was performed by LSD method at 5% probability. Columns with similar letters did not differ significantly.

**Table S3.** The mean comparison of simple effect of different levels of water deficit on evaluated traits of Cowpea.

| **Treatment** | **PH** | **NN** | **NL** | **SD** | **PD** | **PL** | **PW20** | **GW100** | **NP** |
| --- | --- | --- | --- | --- | --- | --- | --- | --- | --- |
| **Irri** |  |  |  |  |  |  |  |  |  |
| **8** | 27.3±1.37 a | 12.1±0.81 a | 3.2±0.29 a | 7.19±1.01 a | 7.51±0.5 a | 12.99±1.05 a | 32.89±1.61 a | 15.16±0.18 a | 11 ±0.93 a |
| **12** | 23.78±1.58 b | 10.93±0.7 b | 2.41±0.16 b | 7.04±0.67 a | 6.44±0.53 b | 11±0.19 b | 27.41±1.54 b | 13.16±1.05 b | 9±0.56 b |
| **16** | 17.99±1.76 c | 9.12±1.75 c | 1.14±0.32 c | 6.44±0.64 a | 5.05±0.92 c | 9.03±1.48 c | 22.38±1.65 c | 10.64±0.18 c | 7±0.67 c |

**Table S3** (continued).

| **Treatment** | **LL** | **Yield** | **LW** | **NGP** | **Chla** | **Chlb** | **Total Chl** | **Car** | **Prolin** | **Suger** |
| --- | --- | --- | --- | --- | --- | --- | --- | --- | --- | --- |
| **Irri** |  |  |  |  |  |  |  |  |  |  |
| **8** | 6.964±0.06 a | 1982.37±30.25 a | 4.72±0.82 a | 9.26±0.26 a | 12.77±1.7 a | 6.38±1.03 a | 19.15±2.51 a | 13.93±1.51 a | 2.77±0.61 c | 3.64±0.69 c |
| **12** | 6.29±0.81 b | 1421.7±76.46 b | 4.28±0.74 a | 8.26±0.28 b | 10.86±1.89 b | 4.87±0.97 b | 15.73±2.73 b | 11.34±1.46 b | 3.6±0.77 b | 4.94±0.81 b |
| **16** | 6.2±0.98 b | 1320.32±22.57 c | 4.05±1.26 a | 6.33±0.41 c | 7.75±2.19 c | 3.49±1.13 c | 11.24±1.22 c | 8.08±1.46 c | 4.66±1.13 a | 6.33±0.98 a |

| **Treatment** | **Phenol** | **Protein** | **Flavonoid** | **MDA** | **SOD** | **APX** | **GPX** | **CAT** | **PPO** |
| --- | --- | --- | --- | --- | --- | --- | --- | --- | --- |
| **Irri** |  |  |  |  |  |  |  |  |  |
| **8** | 26.17±2.49 c | 157.11±33.43 c | 13.48±2.39 c | 5.73±0.71 c | 0.03±0.005 c | 0.05±0.001 c | 0.05±0.001 c | 0.02±0.006 c | 0.03±0.001 c |
| **12** | 33.83±2.03 b | 188.66±47.38 b | 18.66±2.12 b | 7.33±1.38 b | 0.04±0.006 b | 0.08±0.002 b | 0.06±0.001 b | 0.04±0.005 b | 0.04±0.001 b |
| **16** | 47.05±2.5 a | 241.38±7.31 a | 27.52±1.22 a | 10.66±2.57 a | 0.05±0.009 a | 0.09±0.004 a | 0.08±0.003 a | 0.06±0.009 a | 0.06±0.099 a |

**Table S3** (continued).

**Irri:** Different levels of water deficit, **PH:** Plant height, **NN:** Number of nodes, **NL:** Distance internode, **SD:** Stem diameter, **PD:** PPO diameter, **PL:** PPO length, **PW 20:** Weight 20 PPOs, **GW 100:** Weight 100 grains, **NP:** Number of PPOs , **LL:** Leaf length , **Yi:** Yield, **LW:** Leaf width, **NGP:** Number of seeds in PPO, **Chla:** Chlorophyll a, **Chlb:** Chlorophyll b, **ChlT:** Total chlorophyll, **Car:** Carotenoid, , **MDA**: Malondialdehyde, **CAT:** Catalase, **SOD:** Superoxide dismutase, **GPX:** Guaiacol peroxidase, **APX:** Ascorbate peroxidase, **PPO:** Polyphenol oxidase.* and ** Significantly at the probability level of %5 and %1, respectively. Mean comparison was performed by LSD method at 5% probability. Columns with similar letters did not differ significantly.

**Table S4.** The mean comparison of simple effect of different 24-epibrassinolide levels on evaluated traits of Cowpea.

| **Treatment** | **PH** | **NN** | **NL** | **SD** | **PD** | **PL** | **PW20** | **GW100** | **NP** | **LL** |
| --- | --- | --- | --- | --- | --- | --- | --- | --- | --- | --- |
| **B** |  |  |  |  |  |  |  |  |  |  |
| **0** | 25.16±5.87 a | 11.09±0.72 b | 1.88±1.06 a | 7.08±0.26 a | 6±0.59 c | 11.82±1.26 a | 24.81±4.18 c | 12.57±2.6 c | 8±1.11 b | 6±0.91 b |
| **5** | 26.03±7.79 a | 11.15±0.71 ab | 2±4.82 a | 7.1±0.71 a | 6.97±0.32 b | 11.91±0.94 a | 30.96±1.52 b | 14.09±2.24 b | 9±1.55 b | 6.34±1.14 ab |
| **10** | 26.93±7.08 a | 12.72±1.74 a | 2.99±1.18 a | 7.23±0.9 a | 7.93±0.95 a | 21.9±2.51 a | 35.6±2.08 a | 16.81±2.72 a | 11±1.43 a | 7.14±0.78 a |

**Table S4** (continued).

| **Treatment** | **Yield** | **LW** | **NGP** | **Chla** | **Chlb** | **Total Chl** | **Car** | **Prolin** | **Suger** |
| --- | --- | --- | --- | --- | --- | --- | --- | --- | --- |
| **B** |  |  |  |  |  |  |  |  |  |
| **0** | 1521.96±21.4 c | 4.61±0.79 a | 8 ±0.5 c | 8.37±2.38 c | 3.94±1.21 c | 12.31±3.5 c | 9.61±2.49 c | 2.66±0.47 c | 4.19±1.05 c |
| **5** | 1577.85±29.24 b | 4.32±0.86 a | 9.27±0.46 b | 10.8±2.5 b | 4.88±1.5 b | 15.69±3.9 b | 11.24±2.66 b | 3.75±0.96 b | 4.95±1.21 b |
| **10** | 1611.79±20.97 a | 5.11±1.25 a | 11±0.41 a | 12.08±2.32 a | 5.84±1.41 a | 17.92±3.56 a | 12.38±2.62 a | 4.61±1.19 a | 5.78±1.38 a |

**Table S4** (continued).

| **Treatment** | **Protein** | **Phenol** | **Flavonoid** | **MDA** | **SOD** | **APX** | **GPX** | **CAT** | **PPO** |
| --- | --- | --- | --- | --- | --- | --- | --- | --- | --- |
| **B** |  |  |  |  |  |  |  |  |  |
| **0** | 158.68±32.11 c | 27.34±6.71 b | 17.8±5.63 c | 9.22±2.73 a | 0.04±0.008 c | 0.06±0.01 c | 0.05±0.001 c | 0.04±0.006 a | 0.03±0.001 c |
| **5** | 188.88±42.74 b | 33.33±7.12 b | 19.75±6.36 b | 8.11±2.44 b | 0.04±0.009 b | 0.07±0.02 b | 0.06±0.001 b | 0.03±0.005 b | 0.04±0.001 b |
| **10** | 239.63±75.69 a | 46.41±37.74 a | 22.25±7.64 a | 6.49±2.22 c | 0.05±0.001 a | 0.1±0.03 a | 0.08±0.003 a | 0.03±0.009 b | 0.61±0.099 a |

**B:** Different concetrations of 24-epibrassinolide**, PH:** Plant height, **NN:** Number of nodes, **NL:** Distance internode, **SD:** Stem diameter, **PD:** PPO diameter, **PL:** PPO length, **PW 20:** Weight 20 PPOs, **GW 100:** Weight 100 grains, **NP:** Number of PPOs , **LL:** Leaf length , **Yi:** Yield, **LW:** Leaf width, **NGP:** Number of seeds in PPO, **Chla:** Chlorophyll a, **Chlb:** Chlorophyll b, **ChlT:** Total chlorophyll, **Car:** Carotenoid, , **MDA**: Malondialdehyde, **CAT:** Catalase, **SOD:** Superoxide dismutase, **GPX:** Guaiacol peroxidase, **APX:** Ascorbate peroxidase, **PPO:** Polyphenol oxidase.

* and ** Significantly at the probability level of %5 and %1, respectively. Mean comparison was performed by LSD method at 5% probability. Columns with similar letters did not differ significantly.

**Table S5.** The mean comparison of simple effect of different yeast extract levels on evaluated traits of Cowpea.

| **Treatment** | **PH** | **NN** | **NL** | **SD** | **PD** | **PL** | **PW20** | **GW100** | **NP** | **LL** |
| --- | --- | --- | --- | --- | --- | --- | --- | --- | --- | --- |
| **Yeast** |  |  |  |  |  |  |  |  |  |  |
| **0** | 25.75±6.71 a | 10.94±1.47 a | 1.64±4.02 a | 7.19±0.93 a | 7.01±0.8 a | 12.15±1.15 b | 30.34±0.58 b | 12.3±1.56 b | 7.34±1.34 b | 5.82±0.97 b |
| **12** | 26.34±7.23 a | 11.17±0.78 a | 2.94±1.05 a | 7.97±95.32 a | 7.98±0.52 a | 14.6±0.99 a | 35.61±0.92 a | 15.34±1.51 a | 9.65±1 a | 7.92±0.74 a |

**Table S5** (continued).

| **Treatment** | **Yield** | **LW** | **NGP** | **Chla** | **Chlb** | **Total Chl** | **Car** | **Prolin** | **Suger** |
| --- | --- | --- | --- | --- | --- | --- | --- | --- | --- |
| **Yeast** |  |  |  |  |  |  |  |  |  |
| **0** | 1457.33±69.33 b | 4.21±0.22 b | 7.35±1.23 a | 9.72±2.55 b | 4.4±1.52 b | 14.16±1.99 b | 10.72±1.49 b | 3.4±1.05 a | 4.53±1.08 b |
| **12** | 1684.91±36.41 a | 5.95±0.6 a | 8.55±1.33 a | 11.16±2.95 a | 5.37±1.49 a | 16.54±1.3 a | 13.47±1.07 a | 3.98±1.31 a | 5.44±1.02 a |

**Table S5** (continued).

| **Treatment** | **Phenol** | **Protein** | **Flavonoid** | **MDA** | **SOD** | **APX** | **GPX** | **CAT** | **PPO** |
| --- | --- | --- | --- | --- | --- | --- | --- | --- | --- |
| **Yeast** |  |  |  |  |  |  |  |  |  |
| **0** | 34.48±2.48 b | 168.75±5.66 b | 17.98±3.85 b | 8.45±2.93 a | 0.04±0.001 b | 0.06±0.001 b | 0.05±0.001 b | 0.04±0.004 a | 0.14±0.081 a |
| **12** | 37.09±1.57 a | 223.92±6.03 a | 21.96±2.14 a | 7.4±2.39 b | 0.05±0.001 a | 0.08±0.004 a | 0.08±0.003 a | 0.03±0.009 b | 0.05±0.001 b |

**PH:** Plant height, **NN:** Number of nodes, **NL:** Distance internode, **SD:** Stem diameter, **PD:** PPO diameter, **PL:** PPO length, **PW 20:** Weight 20 PPOs, **GW 100:** Weight 100 grains, **NP:** Number of PPOs , **LL:** Leaf length , **Yi:** Yield, **LW:** Leaf width, **NGP:** Number of seeds in PPO, **Chla:** Chlorophyll a, **Chlb:** Chlorophyll b, **ChlT:** Total chlorophyll, **Car:** Carotenoid, , **MDA**: Malondialdehyde, **CAT:** Catalase, **SOD:** Superoxide dismutase, **GPX:** Guaiacol peroxidase, **APX:** Ascorbate peroxidase, **PPO:** Polyphenol oxidase.

* and ** Significantly at the probability level of %5 and %1, respectively. Mean comparison was performed by LSD method at 5% probability. Columns with similar letters did not differ significantly.

**Table S6.** The mean comparison of interaction effects of different levels of water deficit and year crops on evaluated traits of Cowpea

| **Treatment** | | **PH** | **NN** | **NL** | **SD** | **PD** | **PL** | **PW20** | **GW100** | **NP** | **LL** |
| --- | --- | --- | --- | --- | --- | --- | --- | --- | --- | --- | --- |
| **year** | **Irri** |  |  |  |  |  |  |  |  |  |  |
| **1** | **8** | 21.82±2.76 b | 10.78±0.89 a | 1.57±2.39 b | 6.83±0.61 a | 7.1±0.7 a | 12.13±1.21 a | 39.55±4.98 a | 16.72±3.12 a | 10.08±0.92 a | 6.06±0.83 c |
|  | **12** | 21.24±1.22 b | 10.68±0.39 a | 1.03±0.17 b | 6.6±0.95 a | 7±0.69 a | 11.59±1.4 a | 34.32±6.52 b | 15.66±1.84 ab | 8.09±0.56 b | 6.02±0.66 c |
|  | **16** | 20.15±2.09 b | 10.42±0.5 a | 0.84±0.13 b | 5.57±163.57 a | 7±0.47 a | 11.57±1.41 a | 34.05±7.55 b | 14.99±2.02 b | 7.22±0.76 c | 5.94±0.71 c |
| **2** | **8** | 32.41±6.68 a | 11.55±2.04 a | 2.56±6.12 a | 7.7±0.8 a | 7.11±1.23 a | 12.48±3.19 a | 26.65±2.85 c | 13.33±1.99 c | 10.08±0.97 a | 7.16±1.01 a |
|  | **12** | 31.61±6.66 a | 11.45±0.46 a | 2.09±0.57 ab | 7.42±0.67 a | 6.91±0.16 a | 11.84±0.88 a | 24.44±5.14 c | 12.71±1.32 c | 8.22±0.57 b | 6.63±0.79 b |
|  | **16** | 28.75±6.06 a | 11.39±0.64 a | 1±0.68 b | 7.3±0.7 a | 6.87±0.29 a | 11.64±0.98 a | 24.37±3.23 c | 12.55±1.22 c | 7.35±0.58 c | 6.37±1.22 bc |

**Table S6** (continued).

| **Treatment** | | **Yield** | **LW** | **NGP** | **Chla** | **Chlb** | **Total Chl** | **Car** | **Prolin** | **Suger** |
| --- | --- | --- | --- | --- | --- | --- | --- | --- | --- | --- |
| **year** | **Irri** |  |  |  |  |  |  |  |  |  |
| **1** | **8** | 2225.49±370.89 a | 4.32±0.65 a | 9.56±1.4 a | 12.81±1.78 a | 6.44±1.1 a | 19.25±2.55 a | 13.66±1.5 a | 2.72±0.61 c | 3.6±0.0.71 c |
|  | **12** | 1518.6±186.34 c | 4.22±0.42 a | 8.73±2 a | 10.88±2.02 b | 4.85±0.98 b | 15.73±2.87 b | 11.27±1.52 b | 3.6±0.78 b | 4.91±0.84 b |
|  | **16** | 1513.05±312.96 c | 4.02±0.55 a | 8.36±1.48 a | 7.77±2.28 c | 3.5±1.08 c | 11.28±3.27 c | 8.11±1.49 c | 4.61±1.28 a | 6.33±0.99 a |
| **2** | **8** | 1909.83±280.81 b | 5.87±7.44 a | 8.92±1.03 a | 12.61±1.71 a | 6.33±0.98 a | 18.94±2.53 a | 14.5±1.55 a | 2.77±0.63 c | 3.66±0.7 c |
|  | **12** | 1330.35±204.3 cd | 5.05±0.84 a | 8.16±1.07 bc | 10.83±1.82 b | 4.89±0.99 b | 15.72±2.67 b | 11.41±1.43 b | 3.6±0.78 b | 4.96±0.81 b |
|  | **16** | 1122.03±310.24 d | 4.55±0.82 a | 7.93±0.28 c | 7.72±2.16 c | 3.48±1.21 c | 11.2±3.26 c | 8.05±1.47 c | 4.71±1.37 a | 6.33±1 a |

**Table S6** (continued).

| **Treatment** | | **Protein** | **Phenol** | **Flavonoid** | **MDA** | **SOD** | **APX** | **GPX** | **CAT** | **PPO** |
| --- | --- | --- | --- | --- | --- | --- | --- | --- | --- | --- |
| **year** | **Irri** |  |  |  |  |  |  |  |  |  |
| **1** | **8** | 156.9±33.31 c | 26.01±5.61 b | 13.43±2.37 c | 5.85±0.72 c | 0.035±0.005 c | 0.054±0.001 d | 0.049±0.001 c | 0.026±0.006 d | 0.03±0.009 c |
|  | **12** | 188.05±47.13 b | 33.66±7.32 b | 18.44±2.09 b | 7.46±1.25 b | 0.045±0.006 b | 0.08±0.002 c | 0.064±0.001 b | 0.067±0.007 a | 0.04±0.001 b |
|  | **16** | 247±59.37 a | 40.94±7.14 ab | 27.88±4.25 a | 11±1.87 a | 0.057±0.007 a | 0.109±0.003 a | 0.087±0.003 a | 0.038±0.008 b | 0.05±0.001 ab |
| **2** | **8** | 156.66±34.49 c | 26.11±5.52 b | 13.35±2.47 c | 5.61±0.7 c | 0.036±0.005 c | 0.056±0.001 d | 0.05±0.001 c | 0.026±0.006 d | 0.03±0.001 c |
|  | **12** | 189.27±48.98 b | 34±6.93 b | 18.88±2.19 b | 7.2±1.53 b | 0.046±0.007 b | 0.081±0.002 c | 0.065±0.001 b | 0.03±0.005 c | 0.04±0.01 b |
|  | **16** | 235.77±81.15 a | 53.16±52.59 a | 27.16±6.14 a | 10.36±3.1 a | 0.059±0.001 a | 0.089±0.004 b | 0.086±0.003 a | 0.039±0.009 b | 0.058±0.04 a |

**Irri:** Different levels of water deficit, **PH:** Plant height, **NN:** Number of nodes, **NL:** Distance internode, **SD:** Stem diameter, **PD:** PPO diameter, **PL:** PPO length, **PW 20:** Weight 20 PPOs, **GW 100:** Weight 100 grains, **NP:** Number of PPOs , **LL:** Leaf length , **Yi:** Yield, **LW:** Leaf width, **NGP:** Number of seeds in PPO, **Chla:** Chlorophyll a, **Chlb:** Chlorophyll b, **ChlT:** Total chlorophyll, **Car:** Carotenoid, , **MDA**: Malondialdehyde, **CAT:** Catalase, **SOD:** Superoxide dismutase, **GPX:** Guaiacol peroxidase, **APX:** Ascorbate peroxidase, **PPO:** Polyphenol oxidase.

* and ** Significantly at the probability level of %5 and %1, respectively. Mean comparison was performed by LSD method at 5% probability. Columns with similar letters did not differ significantly.

**Table S7.** The mean comparison of interaction effects of different yeast extract levels and 24-epibrassinolide on evaluated traits of Cowpea

| **Treatment** | | **PH** | **NN** | **NL** | **SD** | **PD** | **PL** | **PW20** | **GW100** | **NP** | **LL** |
| --- | --- | --- | --- | --- | --- | --- | --- | --- | --- | --- | --- |
| **Yeast** | **B** |  |  |  |  |  |  |  |  |  |  |
| **0** | **0** | 24.34±6.92 a | 10.42±2.29 b | 1.78±1.06 b | 6.95±0.97 a | 6.78±0.44 b | 11.42±1.19 a | 28.27±5.75 b | 12.64±0.52 d | 8.04±1.47 d | 5.59±0.64 c |
|  | **5** | 25.79±4.98 a | 11.02±0.66 a | 1.88±1.04 b | 7.04±0.77 a | 6.94±0.35 ab | 11.67±0.91 a | 30±6.73 ab | 14.14±0.31 c | 8.07±1.01 d | 5.93±0.72 bc |
|  | **10** | 26.24±6.85 a | 11.09±0.77 ab | 1.94±1.08 b | 7.12±0.84 a | 7±0.47 ab | 11.73±0.88 a | 30.1±7.8 ab | 16.38±0.3 b | 8.37±1.4 cd | 6.22±0.74 b |
| **12** | **0** | 25.75±7.36 a | 11.03±0.92 ab | 1.95±1.08 b | 7.16±0.67 a | 7±0.31 ab | 11.98±1.53 a | 30.92±9.21 ab | 13.47±0.28 cd | 8.61±1.14 bc | 6.35±0.79 b |
|  | **5** | 26.73±7.8 a | 11.23±0.75 a | 2.12±1.32 b | 7.58±0.96 a | 7.01±0.71 ab | 12.09±0.91 a | 31.82±7.35 a | 16.55±0.52 b | 8.78±1.58 ab | 7.02±0.92 a |
|  | **10** | 27.13±8.01 a | 11.31±0.71 a | 3.03±6.67 a | 9.4±1.34 a | 7.25±1.27 a | 12.38±3.32 a | 32.28±9.16 a | 18.78±0.91 a | 9.1±1.4 a | 7.08±1.05 a |

**Table S7** (continued).

| **Treatment** | | **Yield** | **LW** | **NGP** | **Chla** | **Chlb** | **Total Chl** | **Car** | **Prolin** | **Suger** |
| --- | --- | --- | --- | --- | --- | --- | --- | --- | --- | --- |
| **Yeast** | **B** |  |  |  |  |  |  |  |  |  |
| **0** | **0** | 1425.95±247.16 bc | 5.03±0.22 c | 8.05±0.69 bc | 8.2±2.38 e | 3.77±1.38 d | 12.17±3.69 e | 9.27±2.5 d | 2.53±0.42 e | 3.87±0.9 e |
|  | **5** | 1500.25±154.27 b | 5.14±0.48 c | 8.26±0.55 b | 9.61±2.4 d | 4.33±1.48 c | 13.94±3.77 d | 10.76±2.53 c | 3.39±0.82 c | 4.54±1.12 d |
|  | **10** | 1525.14±228.77 b | 5.9±0.29 b | 8.37±0.3 b | 11.05±2.31 c | 5.21±1.42 b | 16.27±3.63 c | 11.44±2.28 b | 4.27±1 b | 5.18±1.19 c |
| **12** | **0** | 1529.78±366.81 b | 6.29±0.3 ab | 8.69±0.35 b | 8.77±2.46 e | 4.26±1 c | 12.93±3.39 e | 11±2.41 bc | 2.76±0.47 d | 4.46±1.08 d |
|  | **5** | 1628.45±285.68 a | 6.74±0.57 a | 8.92±0.24 ab | 12±2.09 b | 5.44±1.32 b | 17.44±3.26 b | 11.72±2.78 b | 4.12±0.98 b | 5.37±1.23 b |
|  | **10** | 1693.79±248.91 a | 6.94±0.19 a | 9.55±0.39 a | 13.11±1.87 a | 6.47±1.11 a | 19.58±2.65 a | 13.33±2.65 a | 4.95±1.29 a | 6.38±1.32 a |

**Table S7** (continued).

| **Treatment** | | **Phenol** | **Protein** | **Flavonoid** | **MDA** | **SOD** | **APX** | **GPX** | **CAT** | **PPO** |
| --- | --- | --- | --- | --- | --- | --- | --- | --- | --- | --- |
| **Yeast** | **B** |  |  |  |  |  |  |  |  |  |
| **0** | **0** | 24.5±5.13 c | 146.88±30.76 e | 16.33±5.14 e | 9.72±2.84 a | 0.038±0.008 d | 0.05±0.001 f | 0.047±0.009 e | 0.02±0.006 d | 0.03±0.007 ef |
|  | **5** | 29.27±5.34 bc | 165.83±31.77 d | 18.11±5.89 d | 8.66±2.58 b | 0.042±0.008 c | 0.07±0.001 d | 0.055±0.001 d | 0.03±0.005 c | 0.032±0.009 e |
|  | **10** | 49.66±53.45 a | 193.55±58.11 c | 19.5±6.35 c | 6.94±2.78 c | 0.05±0.008 b | 0.08±0.001 c | 0.059±0.001 c | 0.04±0.007 b | 0.037±0.007 d |
| **12** | **0** | 29.9±6.99 bc | 169.73±29.39 d | 18.98±5.86 cd | 8.52±2.64 b | 0.042±0.008 c | 0.06±0.001 e | 0.057±0.001 cd | 0.03±0.006 c | 0.04±0.001 c |
|  | **5** | 37.88±6.4 abc | 211.94±40.32 b | 21.38±6.55 b | 7.56±2.16 c | 0.049±0.001 b | 0.097±0.003 b | 0.075±0.001 b | 0.04±0.008 b | 0.05±0.004 b |
|  | **10** | 43.16±7.27 ab | 285.72±62.61 a | 25±8 a | 6.03±1.48 d | 0.056±0.001 a | 0.12±0.004 a | 0.108±0.003 a | 0.06±0.003 a | 0.06±0.001 a |

**B:** Different 24-epibrassinolide levels, **PH:** Plant height, **NN:** Number of nodes, **NL:** Distance internode, **SD:** Stem diameter, **PD:** PPO diameter, **PL:** PPO length, **PW 20:** Weight 20 PPOs, **GW 100:** Weight 100 grains, **NP:** Number of PPOs , **LL:** Leaf length , **Yi:** Yield, **LW:** Leaf width, **NGP:** Number of seeds in PPO, **Chla:** Chlorophyll a, **Chlb:** Chlorophyll b, **ChlT:** Total chlorophyll, **Car:** Carotenoid, , **MDA**: Malondialdehyde, **CAT:** Catalase, **SOD:** Superoxide dismutase, **GPX:** Guaiacol peroxidase, **APX:** Ascorbate peroxidase, **PPO:** Polyphenol oxidase.

* and ** Significantly at the probability level of %5 and %1, respectively. Mean comparison was performed by LSD method at 5% probability. Columns with similar letters did not differ significantly.

**Table S8.** The mean comparison of interaction effects of different year crops and 24-epibrassinolide (B) on evaluated traits of Cowpea

| **Treatment** | | **PH** | **NN** | **NL** | **SD** | **PD** | **PL** | **PW20** | **GW100** | **NP** | **LL** |
| --- | --- | --- | --- | --- | --- | --- | --- | --- | --- | --- | --- |
| **Year** | **B** |  |  |  |  |  |  |  |  |  |  |
| **1** | **0** | 20.84±1.79 c | 10.74±0.54 c | 0.92±0.17 c | 45.33±168.36 a | 6.87±0.81 a | 11.76±1.7 a | 32.94±6.82 b | 16.1±2.87 a | 8.13±0.98 b | 6.09±0.62 b |
|  | **5** | 21.41±2.02 c | 10.45±0.76 c | 1.53±2.4 bc | 6.88±0.73 a | 7.11±0.35 a | 11.9±1.02 a | 36.49±6.36 a | 15.29±1.99 a | 8.41±1.63 ab | 5.91±0.91 b |
|  | **10** | 29.13±7.77 b | 10.69±0.56 c | 0.98±0.24 c | 6.8±1.08 a | 7.12±0.63 a | 11.63±1.33 a | 38.49±5.91 a | 15.98±2.53 a | 8.77±1.51 a | 6.03±0.62 b |
| **2** | **0** | 20.96±2.72 c | 10.99±2.35 bc | 2.8±0.61 abc | 7.66±0.9 a | 6.92±0.3 a | 11.89±0.68 a | 25.33±2.5 c | 13.06±0.93 b | 8.53±1.21 ab | 7.15±0.86 a |
|  | **5** | 30.62±4.67 ab | 11.6±0.54 ab | 4.45±6.14 a | 7.32±0.64 a | 6.83±0.23 a | 11.91±0.89 a | 25.43±2.79 c | 12.49±1.49 b | 8.42±1.51 ab | 6.76±1.21 a |
|  | **10** | 33.02±6.58 a | 11.8±0.45 a | 3.01±0.8 ab | 7.27±0.62 a | 7.13±1.21 a | 12.17±3.32 a | 24.7±5.84 c | 13.04±2.06 b | 8.7±1.38 a | 6.25±0.91 b |

**Table S8** (continued).

| **Treatment** | | **LW** | **NGP** | **Yield** | **Chla** | **Chlb** | **Total Chl** | **Car** | **Prolin** | **Suger** | **Phenol** |
| --- | --- | --- | --- | --- | --- | --- | --- | --- | --- | --- | --- |
| **year** | **B** |  |  |  |  |  |  |  |  |  |  |
| **1** | **0** | 4.22±0.55 a | 8.5±1.91 a | 1620.96±347.18 b | 8.48±2.49 c | 3.91±1.24 c | 12.54±3.65 c | 9.57±2.4 c | 2.63±0.47 c | 4.13±1.09 c | 27.07±6.86 b |
|  | **5** | 4.15±0.67 a | 9.32±1.81 a | 1795.53±557.53 ab | 10.77±2.51 b | 4.87±1.54 b | 15.64±3.95 b | 11.15±2.68 b | 3.73±0.98 b | 4.95±1.25 b | 33.05±7.1 b |
|  | **10** | 4.19±0.44 a | 8.83±1.36 a | 1840.65±447.53 a | 12.22±2.51 a | 5.86±1.39 a | 18.08±3.65 a | 12.33±2.56 a | 4.57±1.15 a | 5.75±1.41 a | 40.5±7.81 ab |
| **2** | **0** | 4.95±0.85 a | 8.52±1.02 a | 1419.13±252.44 c | 8.38±2.35 c | 3.97±1.2 c | 12.36±3.46 c | 9.75±2.63 c | 2.66±0.49 c | 4.2±1.05 c | 27.33±6.76 b |
|  | **5** | 4.48±1 a | 8.63±0.93 a | 1360.17±302.89 c | 10.83±2.57 b | 4.9±1.51 b | 15.74±3.97 b | 11.33±2.72 b | 3.78±0.98 b | 4.96±1.22 b | 33.61±7.33 b |
|  | **10** | 6.04±7.04 a | 75.85±286.3 a | 1382.92±497.58 c | 11.94±2.18 a | 5.82±1.47 a | 17.76±3.56 a | 12.44±2.74 a | 4.65±1.25 a | 5.8±1.39 a | 52.33±52.89 a |

**Table S8** (continued).

| **Treatment** | | **Protein** | **Flavonoid** | **MDA** | **SOD** | **APX** | **GPX** | **CAT** | **PPO** |
| --- | --- | --- | --- | --- | --- | --- | --- | --- | --- |
| **Year** | **B** |  |  |  |  |  |  |  |  |
| **1** | **0** | 158.01±32.09 c | 17.48±5.65 c | 9.13±2.81 a | 0.04±0.008 d | 0.06±0.01 c | 0.05±0.01 c | 0.06±0.08 a | 0.03±0.01 a |
|  | **5** | 189.72±43.78 b | 19.72±6.46 b | 8.28±2.37 ab | 0.045±0.009 c | 0.08±0.02 b | 0.06±0.01 b | 0.02±0.005 c | 0.04±0.01 a |
|  | **10** | 244.22±65.66 a | 22.55±7.42 a | 6.89±1.94 c | 0.052±0.01 b | 0.09±0.03 a | 0.08±0.03 a | 0.03±0.009 b | 0.05±0.01 a |
| **2** | **0** | 158.61±33.07 c | 17.83±5.78 c | 9.1±2.72 a | 0.04±0.008 d | 0.06±0.01 c | 0.05±0.01 c | 0.02±0.006 d | 0.03±0.01 a |
|  | **5** | 188.05±42.92 b | 19.77±6.44 b | 7.94±2.57 b | 0.046±0.009 c | 0.06±0.02 c | 0.06±0.01 b | 0.03±0.005 c | 0.04±0.01 a |
|  | **10** | 235.05±86.25 a | 21.94±8.06 a | 6.1±2.47 c | 0.055±0.01 a | 0.1±0.04 a | 0.08±0.03 a | 0.04±0.01 b | 0.38±1.4 a |

**B:** Different 24-epibrassinolide levels, **PH:** Plant height, **NN:** Number of nodes, **NL:** Distance internode, **SD:** Stem diameter, **PD:** PPO diameter, **PL:** PPO length, **PW 20:** Weight 20 PPOs, **GW 100:** Weight 100 grains, **NP:** Number of PPOs , **LL:** Leaf length , **Yi:** Yield, **LW:** Leaf width, **NGP:** Number of seeds in PPO, **Chla:** Chlorophyll a, **Chlb:** Chlorophyll b, **ChlT:** Total chlorophyll, **Car:** Carotenoid, , **MDA**: Malondialdehyde, **CAT:** Catalase, **SOD:** Superoxide dismutase, **GPX:** Guaiacol peroxidase, **APX:** Ascorbate peroxidase, **PPO:** Polyphenol oxidase.

* and ** Significantly at the probability level of %5 and %1, respectively. Mean comparison was performed by LSD method at 5% probability. Columns with similar letters did not differ significantly.

**Table S9.** The mean comparison of interaction effects of different year crops and yeast extract on evaluated traits of Cowpea

| **Teatment** | | **PH** | **NN** | **NL** | **SD** | **PD** | **PL** | **PW20** | **GW100** | **NP** | **LL** |
| --- | --- | --- | --- | --- | --- | --- | --- | --- | --- | --- | --- |
| **Year** | **yeast** |  |  |  |  |  |  |  |  |  |  |
| **1** | **0** | 21.22±2.47 b | 10.66±0.71 bc | 1.35±1.96 b | 6.85±0.98 a | 6.95±0.57 a | 11.92±1.54 a | 34.79±7.38 a | 15.74±2.67 a | 8.35±1.47 b | 6.31±0.63 b |
|  | **12** | 20.92±1.88 b | 10.59±0.56 c | 0.94±0.2 b | 32.48±136.14 a | 7.12±0.66 a | 11.61±1.11 a | 37.16±6.09 a | 15.84±2.29 a | 8.52±1.36 ab | 5.7±0.7 c |
| **2** | **0** | 30.28±6.57 a | 11.22±1.94 ab | 3.93±5.07 a | 7.52±0.75 a | 7.07±0.99 a | 12.38±2.64 a | 25.88±4.62 b | 12.86±1.39 b | 8.33±1.22 b | 7.32±0.99 a |
|  | **12** | 31.56±6.59 a | 11.71±0.53 a | 2.9±0.44 a | 7.31±0.73 a | 6.85±0.28 a | 11.59±0.88 a | 24.43±3.02 b | 12.87±1.74 b | 8.77±1.46 a | 6.12±0.73 b |

**Table S9** (continued).

| **Treatment** | | **Yield** | **LW** | **NGP** | **Chla** | **Chlb** | **Total Chl** | **Car** | **Prolin** | **Suger** | **Phenol** |
| --- | --- | --- | --- | --- | --- | --- | --- | --- | --- | --- | --- |
| **year** | **yeast** |  |  |  |  |  |  |  |  |  |  |
| **1** | **0** | 1742.57±467.61 a | 4.45±0.53 b | 8.99±1.81 a | 9.7±2.58 b | 4.46±1.48 b | 14.16±4 b | 10.61±2.42 b | 3.38±1.05 b | 4.51±1.19 b | 30.33±8.14 a |
|  | **12** | 1762.19±462.28 a | 3.92±0.46 b | 8.77±1.62 a | 11.28±3.09 a | 5.4±1.58 a | 16.68±4.49 a | 11.41±3.08 a | 3.91±1.27 a | 5.38±1.44 a | 36.75±8.5 a |
| **2** | **0** | 1372.08±398.86 b | 6.36±5.85 a | 53.7±233.7 a | 9.74±2.56 b | 4.41±1.59 b | 14.15±4.06 b | 10.83±2.61 b | 3.41±1.07 b | 4.55±1.19 b | 38.62±45.27 a |
|  | **12** | 1402.73±32.32 b | 3.95±0.72 b | 8.3±0.95 a | 11.03±2.86 a | 5.38±1.42 a | 16.42±4.19 a | 11.51±3.13 a | 3.98±1.37 a | 5.42±1.43 a | 36.88±8.79 a |

**Table S9** (continued).

| **Treatment** | | **Protein** | **Flavonoid** | **MDA** | **SOD** | **APX** | **GPX** | **CAT** | **PPO** |
| --- | --- | --- | --- | --- | --- | --- | --- | --- | --- |
| **Year** | **yeast** |  |  |  |  |  |  |  |  |
| **1** | **0** | 172.88±43.67 b | 18.22±5.91 b | 8.69±2.66 a | 0.042±0.009 b | 0.06±0.01 c | 0.05±0.01 b | 0.05±0.06 a | 0.03±0.008 a |
|  | **12** | 221.74±64.21 a | 21.62±7.11 a | 7.51±2.34 bc | 0.049±0.01 a | 0.09±0.03 a | 0.07±0.03 a | 0.03±0.009 b | 0.05±0.01 a |
| **2** | **0** | 164.63±48.03 b | 17.74±5.9 b | 8.2±3.22 ab | 0.044±0.01 b | 0.06±0.01 c | 0.05±0.01 b | 0.02±0.007 c | 0.25±1.14 a |
|  | **12** | 223.18±68.95 a | 21.96±7.3 a | 7.22±2.34 c | 0.049±0.01 a | 0.08±0.04 b | 0.08±0.03 a | 0.03±0.009 b | 0.05±0.01 a |

**PH:** Plant height, **NN:** Number of nodes, **NL:** Distance internode, **SD:** Stem diameter, **PD:** PPO diameter, **PL:** PPO length, **PW 20:** Weight 20 PPOs, **GW 100:** Weight 100 grains, **NP:** Number of PPOs , **LL:** Leaf length , **Yi:** Yield, **LW:** Leaf width, **NGP:** Number of seeds in PPO, **Chla:** Chlorophyll a, **Chlb:** Chlorophyll b, **ChlT:** Total chlorophyll, **Car:** Carotenoid, , **MDA**: Malondialdehyde, **CAT:** Catalase, **SOD:** Superoxide dismutase, **GPX:** Guaiacol peroxidase, **APX:** Ascorbate peroxidase, **PPO:** Polyphenol oxidase.

* and ** Significantly at the probability level of %5 and %1, respectively. Mean comparison was performed by LSD method at 5% probability. Columns with similar letters did not differ significantly.

**Table S10.** The mean comparison of interaction effects of different levels of water deficit and 24-epibrassinolide (B) on evaluated traits of Cowpea

| **Treatment** | | **PH** | **NN** | **NL** | **SD** | **PD** | **PL** | **PW20** | **GW100** | **NP** | **LL** |
| --- | --- | --- | --- | --- | --- | --- | --- | --- | --- | --- | --- |
| **Irri** | **B** |  |  |  |  |  |  |  |  |  |  |
| **8** | **0** | 26.16±7.03 ab | 11.11±0.61 a | 2.28±1.4 ab | 7.26±0.71 b | 7.11±0.33 a | 12.09±0.93 a | 31.7±7.53 abcd | 13.97±1.82 ab | 9.48±1.03 b | 6.62±1.15 ab |
|  | **5** | 27.7±8.53 a | 11.05±0.58 a | 2.74±2.79 ab | 7.34±0.81 b | 7.08±0.35 a | 12.16±1.54 a | 32.97 ±5.36ab | 14.7±2.17 ab | 10.14±0.93 a | 6.29±0.84 bc |
|  | **10** | 28.1±6.93 a | 11.45±0.69 a | 3.16±7.88 a | 8.99±0.19 a | 7.34±1.52 a | 12.28±3.98 a | 34.63±9.56 a | 15.44±3.72 a | 10.49±0.64 a | 6.92±1.13 a |
| **12** | **0** | 25.75±7.89 ab | 10.86±0.92 a | 1.93±1.17 ab | 7.07±1.06 b | 6.89±0.36 a | 11.76±1.52 a | 28.75±5.47 de | 13.76±1.54 ab | 8.05±0.78 c | 6.12±1 bc |
|  | **5** | 26.51±6.24 ab | 10.96±0.71 a | 1.94±1 ab | 7.2±0.86 b | 6.92±0.25 a | 11.88±0.89 a | 29.36±10.69 cde | 14.32±2.36 ab | 8.14±0.29 c | 6.17±0.51 bc |
|  | **10** | 26.57±8.97 ab | 11.43±0.82 a | 2.07±1.28 ab | 7.27±1.16 b | 6.96±0.61 a | 12.2±0.93 a | 32.46±10.52 abc | 14.55±1.81 ab | 8.27±0.52 c | 6.57±0.84 ab |
| **16** | **0** | 23.22±6.5 b | 10.64±0.85 a | 1.75±0.91 b | 6.83±1.04 b | 6.84±0.78 a | 11.21±1.06 a | 26.96±5.34 e | 13.62±2.32 b | 6.96±0.75 d | 5.96±0.94 c |
|  | **5** | 25.25±4.27 ab | 10.67±2.85 a | 1.78±0.97 b | 6.7±0.45 b | 6.9±0.44 a | 11.64±1 a | 28.21±4.86 de | 14.09±2.68 ab | 7.45±0.67 d | 6.27±0.129 bc |
|  | **10** | 25.31±6.25 ab | 11.26±0.78 a | 1.89±1.3 ab | 7.2±0.54 b | 6.94±0.55 a | 11.67±1.26 a | 30.04±6.41 bcde | 14.51±3.63 ab | 7.54±0.51 d | 6.36±0.66 abc |

**Table S10** (continued).

| **Treatment** | | **Yield** | **LW** | **NGP** | **Chla** | **Chlb** | **Total Chl** | **Car** | **Prolin** | **Suger** |
| --- | --- | --- | --- | --- | --- | --- | --- | --- | --- | --- |
| **Irri** | **B** |  |  |  |  |  |  |  |  |  |
| **8** | **0** | 1803.31±335.04 b | 4.42±0.67 a | 9.29±1.2 b | 10.97±0.77 c | 5.45±0.5 d | 16.42±1.02 d | 12.44±0.68 c | 2.09±0.13 h | 3.01±0.27 i |
|  | **5** | 2030.17±521.32 a | 4.7±0.82 a | 9.46±1.34 b | 13.08±1.37 b | 6.54±0.78 b | 19.62±1.82 b | 13.97±0.74 b | 2.76±0.38 f | 3.59±0.41 h |
|  | **10** | 2069.5±407.33 a | 4.95±0.91 a | 10.41±350.71 a | 14.08±1.31 a | 7.16±0.83 a | 21.25±1.58 a | 15.16±1.58 a | 3.4±0.31 e | 4.29±0.6 f |
| **12** | **0** | 1393.28±204.66 cd | 4.21±0.66 a | 8.96±1.28 b | 8.83±0.83 e | 3.96±0.27 f | 12.8±0.81 f | 9.79±0.78 d | 2.69±0.19 f | 4.08±0.26 g |
|  | **5** | 1378.88±320.61 cd | 4.23±0.5 a | 9.18±1.79 b | 11.16±1.26 c | 4.76±0.73 e | 15.93±1.87 d | 11.83±1.02 c | 3.67±0.28 d | 4.95±0.33 e |
|  | **10** | 1522.88±318.79 c | 4.57±0.75 a | 8.64±1.57 b | 12.58±1.16 b | 5.87±0.65 c | 18.45±1.45 c | 12.41±0.99 c | 4.45±0.29 c | 5.78±0.59 c |
| **16** | **0** | 1243.18±458.92 d | 4.05±0.77 b | 7.85±1.19 b | 5.5±0.52 f | 2.62±0.56 h | 8.12±0.75 h | 6.75±0.75 f | 3.16±0.17 f | 5.41±0.5 d |
|  | **5** | 1324.49±257.43 cd | 4.2±0.9 a | 8.29±0.99 b | 8.16±1.74 e | 3.35±0.7 g | 11.52±2.41 g | 7.91±0.66 e | 4.83±0.63 b | 6.32±0.61 b |
|  | **10** | 1363.54±148.18 cd | 4±0.5 b | 8.39±1.71 b | 9.58±1.62 d | 4.49±1.15 e | 14.07±2.67 e | 9.58±1.16 d | 5.99±0.78 a | 7.26±0.76 a |

**Table S10** (continued).

| **Treatment** | | **Phenol** | **Protein** | **Flavonoid** | **MDA** | **SOD** | **APX** | **GPX** | **CAT** | **PPO** |
| --- | --- | --- | --- | --- | --- | --- | --- | --- | --- | --- |
| **Irri** | **B** |  |  |  |  |  |  |  |  |  |
| **8** | **0** | 20.19±2 c | 128.85±12.07 e | 11.73±1.36 g | 6.28±0.69 e | 0.03±0.002 f | 0.04±0.004 h | 0.039±0.003 f | 0.02±0.001 g | 0.025±0.004 b |
|  | **5** | 26.58±3.55 bc | 151.08±24.28 d | 13.08±1.78 g | 5.75±0.44 ef | 0.037±0.004 e | 0.05±0.005 g | 0.048±0.008 e | 0.024±0.002 f | 0.033±0.007 b |
|  | **10** | 31.41±2.93 bc | 190.41±24.44 c | 15.58±2.1 f | 5.15±0.46 f | 0.04±0.002 e | 0.06±0.006 f | 0.062±0.01 d | 0.033±0.006 d | 0.042±0.009 b |
| **12** | **0** | 27±2.76 bc | 148.91±14.27 d | 16.66±1.3 f | 8.58±1.05 c | 0.04±0.003 e | 0.05±0.003 g | 0.05±0.005 e | 0.08±0.009 a | 0.033±0.005 b |
|  | **5** | 33.08±4.69 bc | 181.5±16.9 c | 18.58±1.31 e | 7.41±0.9 d | 0.044±0.003 d | 0.08±0.009 d | 0.064±0.001 cd | 0.03±0.004 e | 0.043±0.008 b |
|  | **10** | 41.41±3.65 b | 235.58±49.72 b | 20.75±1.35 d | 6±0.75 ef | 0.052±0.005 c | 0.1±0.02 b | 0.079±0.01 b | 0.036±0.005 c | 0.053±0.001 b |
| 16 | 0 | 34.41±4.39 bc | 197.16±12.31 c | 24.58±2.27 c | 12.5±1 a | 0.05±0.002 c | 0.08±0.004 c | 0.066±0.007 c | 0.032±0.004 d | 0.046±0.006 b |
|  | 5 | 40.33±4.84 b | 234.08±33.01 b | 27.58±2.64 b | 11.16±1.11 b | 0.056±0.004 b | 0.07±0.004 e | 0.083±0.001 b | 0.035±0.004 c | 0.053±0.01 b |
|  | 10 | 66.41±61.65 a | 292.91±96.95 a | 30.41±7.48 a | 8.33±3 cd | 0.067±0.001 a | 0.13±0.04 a | 0.11±0.004 a | 0.048±0.009 b | 0.056±0.0071 a |

**Irri:** Different levels of water deficit, **B:** Different 24-epibrassinolide levels**, PH:** Plant height, **NN:** Number of nodes, **NL:** Distance internode, **SD:** Stem diameter, **PD:** PPO diameter, **PL:** PPO length, **PW 20:** Weight 20 PPOs, **GW 100:** Weight 100 grains, **NP:** Number of PPOs , **LL:** Leaf length , **Yi:** Yield, **LW:** Leaf width, **NGP:** Number of seeds in PPO, **Chla:** Chlorophyll a, **Chlb:** Chlorophyll b, **ChlT:** Total chlorophyll, **Car:** Carotenoid, , **MDA**: Malondialdehyde, **CAT:** Catalase, **SOD:** Superoxide dismutase, **GPX:** Guaiacol peroxidase, **APX:** Ascorbate peroxidase, **PPO:** Polyphenol oxidase.

* and ** Significantly at the probability level of %5 and %1, respectively. Mean comparison was performed by LSD method at 5% probability. Columns with similar letters did not differ significantly.

**Table S11.** The mean comparison of interaction effects of different levels of water deficit and yeast extract on evaluated traits of Cowpea

| **Treatment** | | **PH** | **NN** | **NL** | **SD** | **PD** | **PL** | **PW20** | **GW100** | **NP** |
| --- | --- | --- | --- | --- | --- | --- | --- | --- | --- | --- |
| **Irri** | **yeast** |  |  |  |  |  |  |  |  |  |
| **8** | **0** | 27.34±5.96 a | 11.21±0.85 a | 3.58±6.45 a | 7.22±1.11 a | 6.93±0.62 a | 12.28±1.08 ab | 33.06±7.39 a | 14.16±1.86 a | 9.89±0.69 a |
|  | **12** | 26.89±8.82 ab | 10.96±0.78 a | 2.01±1.1 a | 7.07±0.93 a | 7.09±0.35 a | 11.69±0.95 ab | 33.14±8.06 a | 14.21±2.53 a | 10.18±1.12 a |
| **12** | **0** | 26.01±7.72 ab | 10.7±0.65 a | 1.97±1.21 a | 7.16±0.67 a | 6.96±0.31 a | 11.47±1.2 b | 28.01±5.27 b | 14.04±1.77 a | 8.02±0.59 b |
|  | **12** | 25.74±7.65 ab | 11.12±0.73 a | 1.86±1.15 a | 6.91±0.66 a | 6.91±0.69 a | 11.75±1.2 ab | 30.41±9.29 ab | 14.29±2.51 a | 8.29±0.5 b |
| **16** | **0** | 23.9±6.24 b | 10.86±2.35 a | 2.38±2.42 a | 7.19±1 a | 7.14±1.23 a | 12.69±3.29 a | 29.94±9.12 b | 14.71±3.68 a | 7.11±0.72 c |
|  | **12** | 26.09±5.18 ab | 11.38±0.81 a | 1.9±0.94 a | 45.69±163.54 a | 6.96±0.46 a | 11.36±0.8 b | 28.82±6.06 b | 14.57±2.62 a | 7.47±0.59 c |

**Table S11** (continued).

| **Treatment** | | **LL** | **Yield** | **LW** | **NGP** | **Chla** | **Chlb** | **Total Chl** | **Car** | **Prolin** | **Suger** |
| --- | --- | --- | --- | --- | --- | --- | --- | --- | --- | --- | --- |
| **Irri** | **yeast** |  |  |  |  |  |  |  |  |  |  |
| **8** | **0** | 7.13±0.97 a | 1974.94±431.99 a | 5.15±0.78 ab | 9.37±1.24 a | 12±1.23 b | 6.05±0.76 b | 18.05±1.82 b | 13.37±0.76 b | 2.52±0.48 f | 3.25±0.43 f |
|  | **12** | 6.09±0.91 c | 1960.38±441.55 a | 4.23±0.61 b | 9.1±1.31 a | 13.42±1.83 a | 6.71±1.17 a | 20.14±2.67 a | 14.35±1.87 a | 2.98±0.63 e | 4.01±0.68 e |
| **12** | **0** | 6.66±0.81 ab | 1387.61±251.22 b | 4.71±0.65 ab | 8.25±1.14 a | 10.38±1.37 d | 4.48±0.76 d | 14.87±1.99 d | 11.02±1.11 d | 3.39±0.7 d | 4.58±0.59 d |
|  | **12** | 5.91±0.64 c | 1455.79±302.96 b | 3.85±0.57 b | 8.27±1.44 a | 11.33±2.24 c | 5.25±1.03 c | 16.58±3.14 c | 11.66±1.71 c | 3.82±0.79 c | 5.29±0.86 c |
| **16** | **0** | 6.65±1.07 b | 1309.43±395.95 b | 6.36±7.29 a | 76.42±286.17 a | 6.77±1.3 f | 2.77±0.64 e | 9.55±1.83 f | 7.77±0.94 f | 4.28±1.04 b | 5.75±0.7 b |
|  | **12** | 5.74±0.64 c | 1331.2±239.16 b | 3.73±0.54 b | 8.23±1.09 a | 8.72±2.49 e | 4.2±1.06 d | 12.93±3.45 e | 8.38±1.81 e | 5.04±1.46 a | 6.9±0.89 a |

**Table S11**(continued).

| **Treatment** | | **Phenol** | **Protein** | **Flavonoid** | **MDA** | **SOD** | **APX** | **GPX** | **CAT** | **PPO** |
| --- | --- | --- | --- | --- | --- | --- | --- | --- | --- | --- |
| **Irri** | **yeast** |  |  |  |  |  |  |  |  |  |
| **8** | **0** | 23.55±4.44 b | 137.77±22.27 d | 11.94±1.47 f | 6.03±0.67 de | 0.033±0.004 f | 0.05±0.008 f | 0.04±0.004 f | 0.023±0.003 e | 0.027±0.005 a |
|  | **12** | 28.57±5.21 b | 175.78±31.33 c | 14.98±2.08 e | 5.42±0.63 e | 0.038±0.004 e | 0.06±0.009 e | 0.05±0.01 d | 0.029±0.007 d | 0.039±0.009 a |
| **12** | **0** | 30.5±5.94 b | 163.38±22.67 c | 17.61±1.81 d | 7.98±1.34 c | 0.042±0.004 d | 0.06±0.009 d | 0.05±0.007 e | 0.063±0.08 a | 0.035±0.005 a |
|  | **12** | 37.16±6.55 ab | 213.94±54.48 b | 19.72±1.9 c | 6.68±1.12 d | 0.049±0.006 c | 0.09±0.02 b | 0.07±0.01 b | 0.034±0.005 c | 0.052±0.01 a |
| **16** | **0** | 49.38±53.45 a | 205.11±55.17 b | 24.38±4.38 b | 11.33±3.07 a | 0.055±0.01 b | 0.08±0.006 c | 0.06±0.01 c | 0.033±0.006 c | 0.375±1.4 a |
|  | **12** | 44.72±5.3 a | 277.66±65.96 a | 30.66±4.01 a | 10±1.81 b | 0.061±0.007 a | 0.11±0.05 a | 0.1±0.03 a | 0.043±0.008 b | 0.064±0.01 a |

**Irri:** Different levels of water deficit**, PH:** Plant height, **NN:** Number of nodes, **NL:** Distance internode, **SD:** Stem diameter, **PD:** PPO diameter, **PL:** PPO length, **PW 20:** Weight 20 PPOs, **GW 100:** Weight 100 grains, **NP:** Number of PPOs , **LL:** Leaf length , **Yi:** Yield, **LW:** Leaf width, **NGP:** Number of seeds in PPO, **Chla:** Chlorophyll a, **Chlb:** Chlorophyll b, **ChlT:** Total chlorophyll, **Car:** Carotenoid, , **MDA**: Malondialdehyde, **CAT:** Catalase, **SOD:** Superoxide dismutase, **GPX:** Guaiacol peroxidase, **APX:** Ascorbate peroxidase, **PPO:** Polyphenol oxidase.

* and ** Significantly at the probability level of %5 and %1, respectively. Mean comparison was performed by LSD method at 5% probability. Columns with similar letters did not differ significantly.

**Table S12.** The mean comparison of interaction effects of **d**ifferent levels of water deficit, year and 24-epibrassinolide (B) on evaluated traits of Cowpea

| **Traetment** | | | **PH** | **NN** | **NL1** | **SD** | **PD** | **PL** | **PW20** | **GW100** | **NP** |
| --- | --- | --- | --- | --- | --- | --- | --- | --- | --- | --- | --- |
| **year** | **Irri** | **B** |  |  |  |  |  |  |  |  |  |
| **1** | **8** | **0** | 22.78±1.65 de | 11.07±0.68 a-e | 1.04±0.19 b | 6.79±0.98 b | 6.93±0.86 ab | 12.59±0.53 ab | 38.04±5.34 abc | 14.91±1.48 b-g | 9.12±0.92 c |
|  |  | **5** | 20.98±2.32 de | 10.81±0.94 bcde | 1.01±0.16 b | 6.95±0.87 b | 7.36±0.28 ab | 12.25±1.31abc | 43.04±5.27 a | 16.24±1.98 abc | 10.18±0.83 ab |
|  |  | **10** | 21.72±3.76 de | 10.48±1 de | 1.01±0.21 b | 6.06±0.84 b | 7.01±0.88 ab | 11.54±1.4 bc | 37.56±2.07 bc | 15.83±2.01 abcd | 10.68±0.47 a |
|  | **12** | **0** | 19.78±1.52 e | 10.63±0.35 cde | 0.76±0.14 b | 6.7±0.69 b | 6.66±1.1 b | 10.56±1 c | 28.99±6.6 fg | 15.39±1.99 bcde | 7.86±0.19 def |
|  |  | **5** | 20.09±1.57 e | 10.55±0.14 de | 0.87±0.08 b | 6.71±0.6 b | 7.05±0.3 ab | 11.83±1.04abc | 32.03±3.12 def | 14.46±2.1 c-g | 8.21±0.84 de |
|  |  | **10** | 20.57±3.11 e | 10.07±0.7 e | 0.88±0.14 b | 7.06±0.59 b | 7.29±0.33 ab | 12.38±1.57 abc | 41.13±6.72 ab | 15.13±2.22 b-f | 8.2±0.48 de |
|  | **16** | **0** | 21.67±1.46 de | 10.53±0.41 de | 0.96±0.11 b | 122.47±283.42a | 7±0.46 ab | 12.12±2.27 abc | 31.79±6.46 ef | 18.01±3.77 a | 7.4±0.6 fg |
|  |  | **5** | 21.44±1.37 de | 10.73±0.34 bcde | 2.72±4.12 b | 6.97±0.81 b | 6.94±0.36 ab | 11.62±0.71 bc | 34.4±5.62 cde | 15.18±1.8 bcde | 6.83±0.94 g |
|  |  | **10** | 20.59±0.5 e | 10.79±0.45 bcde | 1.04±0.34 b | 7.28±1.36 b | 7.05±0.63 ab | 10.98±0.61 bc | 36.78±7.51 bcd | 16.98±3.28 ab | 7.45±0.69 fg |
| **2** | **8** | **0** | 32.19±7.05 ab | 11.83±0.6 abc | 2.83±0.42 b | 7.76±1.26 b | 6.96±0.13 ab | 11.59±0.89 bc | 25.35±3.09 ghi | 12.61±0.44 gh | 9.85±1.12 bc |
|  |  | **5** | 34.43±6.8 a | 11.11±0.42 a-e | 7.31±10.6 a | 7.73±0.56 b | 6.86±0.14 ab | 12.14±0.44 abc | 26.22±1.93 ghi | 11.95±0.91 h | 10.1±1.1 ab |
|  |  | **10** | 30.61±6.87abc | 11.23±0.72 a-e | 3.53±0.76 b | 7.6±0.48 b | 6.92±0.22 ab | 11.8±1.22 abc | 28.38±2.92 fgh | 13.58±1.81 d-h | 10.3±0.77 ab |
|  | **12** | **0** | 30.83±3.24abc | 11.6±0.39 abcd | 3.02±0.8 b | 7.71±0.75 b | 7.02±0.26 ab | 11.86±0.66 abc | 24.92±3.03 ghi | 13.71±1.25 d-h | 8.25±0.26 d |
|  |  | **5** | 33.06±8.58 ab | 11.55±0.37 abcd | 3.27±0.41 b | 6.69±0.29 b | 6.73±0.36 b | 11.92±0.81 abc | 24.4±2.71 ghi | 12.78±2.38 gh | 8.06±0.79 def |
|  |  | **10** | 30.94±7.93abc | 11.2±0.58 a-e | 2.69±0.33 b | 7.34±0.51 b | 6.86±0.22 ab | 11.13±1.31 bc | 23.79±4.26 hi | 13.5±2.39 efgh | 8.35±0.59 d |
|  | **16** | **0** | 28.83±2.69 bc | 11.98±0.29 ab | 2.54±0.57 b | 7.51±0.75 b | 6.8±0.43 b | 12.21±0.33 abc | 25.72±1.46 ghi | 12.87±0.63 fgh | 7.51±0.46 efg |
|  |  | **5** | 31.58±4.77 ab | 12.13±0.24 a | 2.76±0.46 b | 7.55±0.51 b | 6.91±0.11 ab | 11.66±1.31 bc | 25.67±3.65 ghi | 12.75±0.72 gh | 7.1±0.56 g |
|  |  | **10** | 25.84±8.72 dc | 10.55±4.2 de | 2.82±1 b | 6.86±0.71 b | 7.62±2.12 a | 13.58±5.52 a | 21.94±7.94 i | 12.05±1.93 h | 7.45±0.71 fg |

**Table S12** (continued).

| **Traetment** | | | **LL** | **Yield** | **Chla** | **Chlb** | **Total Chl** | **Car** | **Prolin** | **Suger** | **Phenol** | **Protein** |
| --- | --- | --- | --- | --- | --- | --- | --- | --- | --- | --- | --- | --- |
| **year** | **Irri** | **B** |  |  |  |  |  |  |  |  |  |  |
| **1** | **8** | **0** | 6.21±0.96 cdef | 2065.78±151.42 bc | 11.11±0.83 d | 5.57±0.56 cd | 16.69±1.34 e | 12.22±0.54 d | 2.08±0.13 i | 2.96±0.21 i | 20.21±2.28 c | 127.4±14.01 e |
|  |  | **5** | 6.13±0.86 def | 2431.36±413.98 a | 13±1.41 bc | 6.58±0.8 b | 19.58±1.85 bc | 13.78±0.74 c | 2.75±0.4 h | 3.58±0.44 h | 26.16±3.25 bc | 151.5±24.57 e |
|  |  | **10** | 5.84±0.78 ef | 2281.53±421.11 ab | 14.33±1.5 a | 7.16±1.13 a | 21.5±1.7 a | 15±1.67 ab | 3.35±0.37 efg | 4.25±0.64 f | 31.66±3.07 bc | 188.83±25.76 d |
|  | **12** | **0** | 6.15±0.59 def | 1364.82±204.39 fghi | 8.83±0.98 ef | 4.01±0.34 g | 12.85±0.92 gh | 9.66±0.81 e | 2.7±0.22 h | 4.03±0.24 g | 26.66±3.2 bc | 148±14.62 e |
|  |  | **5** | 5.69±1.08 f | 1450.28±359.85 e-i | 11.16±1.47 d | 4.7±0.78 f | 15.86±2.21 e | 11.83±1.16 d | 3.65±0.3 de | 4.93±0.34 e | 33±5.13 bc | 182.33±18.87 d |
|  |  | **10** | 5.99±0.26 def | 1724.04±277.91 cde | 12.66±1.36 c | 5.83±0.75 cd | 18.5±1.76 cd | 12.33±1.03 d | 4.46±0.3 c | 5.77±0.64 c | 41.33±3.98 bc | 233.83±50.03 c |
|  | **16** | **0** | 5.91±0.25 ef | 1534.48±201.03 efgh | 5.5±0.54 g | 2.58±0.51 j | 8.03±0.51 j | 6.83±0.75 g | 3.11±0.14 g | 5.4±0.53 d | 34.33±4.5 bc | 195.66±12.98 d |
|  |  | **5** | 5.91±0.91 ef | 1504.93±162.71 efgh | 8.16±1.72 f | 3.23±0.64 i | 11.49±2.31 i | 7.83±0.75 f | 4.81±0.67 b | 6.33±0.68 b | 40±4.47 bc | 235.33±35.25 c |
|  |  | **10** | 6.26±0.71 cdef | 1516.38±225.04 efgh | 9.66±1.96 e | 4.6±0.89 f | 14.26±2.77 f | 9.66±1.21 e | 5.91±0.59 a | 7.24±0.76 a | 48.5±3.33 b | 310±49.88 a |
| **2** | **8** | **0** | 7.63±0.96 a | 1643.05±329.72 cdef | 10.83±0.75 d | 5.33±0.51 de | 16.16±0.75 e | 12.66±0.81 d | 2.1±0.15 i | 3.05±0.32 i | 20.16±1.94 c | 127.33±11.6 e |
|  |  | **5** | 7.11±1.27 ab | 1628.98±200.62 defg | 13.16±1.47 bc | 6.5±0.83 b | 19.66±1.96 b | 14.16±0.75 bc | 2.78±0.41 h | 3.6±0.43 h | 27±4.09 bc | 150.66±26.31 e |
|  |  | **10** | 6.75±0.66 bcd | 1857.47±282.48 cd | 13.83±1.16 ab | 7.16±0.51 a | 21±1.58 a | 15.33±1.63 a | 3.45±0.27 def | 4.33±0.61 f | 31.16±3.06 bc | 192±25.4 d |
|  | **12** | **0** | 7±0.88 abc | 1362.26±80.78 fghi | 8.83±0.75 ef | 3.92±0.18 gh | 12.75±0.77 h | 9.91±0.8 e | 2.68±0.18 h | 4.12±0.29 fg | 27.33±2.5 bc | 149.83±15.25 e |
|  |  | **5** | 6.55±0.79 bcde | 1307.48±290.53 hi | 11.16±1.16 d | 4.83±0.75 ef | 16±1.67 e | 11.83±0.98 d | 3.69±0.29 d | 4.97±0.35 e | 33.16±4.7 bc | 180.66±16.46 d |
|  |  | **10** | 6.35±0.66 b-f | 1321.32±221.44 ghi | 12.5±1.04 c | 5.91±0.6 c | 18.41±1.23 d | 12.5±1.04 d | 4.43±0.31 c | 5.79±0.6 c | 41.5±3.67 bc | 237.33±54.11 c |
|  | **16** | **0** | 6.81±0.64 abcd | 1252.08±62.35 hij | 5.5±0.54 g | 2.67±0.65 j | 8.17±0.98 j | 6.66±0.81 g | 3.21±0.2 fg | 5.41±0.51 d | 34.5±4.72 bc | 198.66±12.62 d |
|  |  | **5** | 6.63±1.59 bcde | 1144.04±202.92 ij | 8.16±1.94 f | 3.39±0.82 hi | 11.55±2.73 i | 8±0.62 f | 4.86±0.64 b | 6.31±0.6 b | 40.66±5.6 bc | 232.83±33.94 c |
|  |  | **10** | 5.66±1.11 f | 969.98±483.29 j | 9.5±1.37 e | 4.38±1.45 fg | 13.88±2.82 fg | 9.5±1.22 e | 6.08±0.98 a | 7.27±0.84 a | 84.33±87.6 a | 275.83±132.26 b |

**Table S12** (continued).

| **Traetment** | | | **Flavonoid** | **MDA** | **SOD** | **APX** | **GPX** | **CAT** | **PPO** | **LL** | **LW** | **NGP** |
| --- | --- | --- | --- | --- | --- | --- | --- | --- | --- | --- | --- | --- |
| **year** | **Irri** | **B** |  |  |  |  |  |  |  |  |  |  |
| **1** | **8** | **0** | 11.63±1.34 i | 6.28±0.89 fgh | 0.03±0.002 h | 0.043±0.004 k | 0.039±0.004 e | 0.02±0.001 j | 0.024±0.003 b | 6.21±0.96 cdef | 4.37±0.78 b | 10.06±0.49 b |
|  |  | **5** | 13.16±1.72 i | 6.01±0.28 gh | 0.036±0.004 g | 0.056±0.006 i | 0.047±0.008 d | 0.024±0.002 i | 0.033±0.006 b | 6.13±0.86 def | 4.46±0.67 b | 9.89±1.79 b |
|  |  | **10** | 15.5±2.07 h | 5.25±0.44 h | 0.04±0.003 fg | 0.064±0.006 fg | 0.061±0.01 c | 0.032±0.005 fgh | 0.041±0.009 b | 5.84±0.78 ef | 4.14±0.57 b | 9.01±1.46 b |
|  | **12** | **0** | 16.5±1.37 gh | 8.46±0.86 cde | 0.04±0.003 ef | 0.058±0.003 hi | 0.05±0.005 d | 0.134±0.1 a | 0.033±0.005 b | 6.15±0.59 def | 4.17±0.53 b | 7.61±1.42 b |
|  |  | **5** | 18.33±1.21 efg | 7.66±1.03 def | 0.044±0.003 ef | 0.08±0.01 e | 0.063±0.01 c | 0.029±0.004 h | 0.042±0.008 b | 5.69±1.08 f | 3.8±0.78 b | 8.14±1.31 b |
|  |  | **10** | 20.5±1.37 de | 6.26±0.73 fgh | 0.51±0.005 d | 0.102±0.02 c | 0.079±0.02 b | 0.036±0.005 d | 0.053±0.01 b | 5.99±0.26 def | 4.08±0.23 b | 9.37±1.37 b |
|  | **16** | **0** | 24.33±2.16 c | 12.66±1.03 a | 0.05±0.002 d | 0.085±0.005 d | 0.065±0.007 c | 0.032±0.003 fgh | 0.046±0.007 b | 5.91±0.25 ef | 4.12±0.3 b | 8.13±2.45 b |
|  |  | **5** | 27.66±2.8 b | 11.16±1.16 b | 0.056±0.004 c | 0.108±0.02 b | 0.083±0.01 b | 0.035±0.004 def | 0.053±0.01 b | 5.91±0.91 ef | 4.2±0.46 b | 9.92±1.9 b |
|  |  | **10** | 31.66±4.08 a | 9.16±1.47 c | 0.064±0.006 b | 0.132±0.04 a | 0.113±0.04 a | 0.046±0.009 c | 0.064±0.01 b | 6.26±0.71 cdef | 4.34±0.49 b | 8.13±1.16 b |
| **2** | **8** | **0** | 11.83±1.47 i | 6.28±0.56 fgh | 0.03±0.002 h | 0.044±0.003 k | 0.04±0.003 e | 0.02±0.001 j | 0.025±0.004 b | 7.63±0.96 a | 5.53±0.79 b | 8.82±1.37 b |
|  |  | **5** | 13±2 i | 5.5±0.44 h | 0.037±0.004 g | 0.058±0.005 hi | 0.049±0.009 d | 0.02±0.002 j | 0.033±0.007 b | 7.11±1.27 ab | 4.95±0.94 b | 9.02±0.55 b |
|  |  | **10** | 15.66±2.33 h | 5.05±0.5 h | 0.04±0.002 efg | 0.066±0.006 f | 0.062±0.01 c | 0.033±0.006 ef | 0.043±0.009 b | 6.75±0.66 bcd | 4.69±0.69 b | 8.91±1.2 b |
|  | **12** | **0** | 16.83±1.32fgh | 8.7±1.29 cd | 0.04±0.003 fg | 0.06±0.003 gh | 0.051±0.006 d | 0.026±0.002 i | 0.034±0.007 b | 7±0.88 abc | 4.98±0.76 b | 8.1±0.99 b |
|  |  | **5** | 18.83±1.47 def | 7.16±0.75 efg | 0.044±0.004 e | 0.082±0.009 e | 0.066±0.01 c | 0.03±0.004 gh | 0.044±0.009 b | 6.55±0.79 bcde | 4.31±0.74 b | 8.44±0.63 b |
|  |  | **10** | 21±1.41 d | 5.75±0.75 gh | 0.054±0.005 cd | 0.102±0.02 c | 0.079±0.01 b | 0.036±0.005 d | 0.054±0.01 b | 6.35±0.66 b-f | 4.35±0.93 b | 7.94±1.55 b |
|  | **16** | **0** | 24.83±2.56 c | 12.33±1.03 ab | 0.05±0.002 d | 0.086±0.004 d | 0.067±0.007 c | 0.033±0.004 efg | 0.043±0.006 b | 6.81±0.64 abcd | 4.33±0.65 b | 8.65±0.57 b |
|  |  | **5** | 27.5±2.73 b | 11.16±1.16 b | 0.056±0.004 c | 0.049±0.04 j | 0.083±0.01 b | 0.035±0.004 de | 0.053±0.01 b | 6.63±1.59 bcde | 4.2±1.25 b | 8.44±1.41 b |
|  |  | **10** | 29.16±10.14 b | 7.51±4.01 def | 0.07±0.01 a | 0.131±0.04 a | 0.108±0.04 a | 0.05±0.008 b | 1.05±2.42 a | 5.66±1.11 f | 9.1±12.96 a | 210.69±495.95 a |

**Irri:** Different levels of water deficit**, B:** 24-epibrassinolide, **PH:** Plant height, **NN:** Number of nodes, **NL:** Distance internode, **SD:** Stem diameter, **PD:** PPO diameter, **PL:** PPO length, **PW 20:** Weight 20 PPOs, **GW 100:** Weight 100 grains, **NP:** Number of PPOs , **LL:** Leaf length , **Yi:** Yield, **LW:** Leaf width, **NGP:** Number of seeds in PPO, **Chla:** Chlorophyll a, **Chlb:** Chlorophyll b, **ChlT:** Total chlorophyll, **Car:** Carotenoid, , **MDA**: Malondialdehyde, **CAT:** Catalase, **SOD:** Superoxide dismutase, **GPX:** Guaiacol peroxidase, **APX:** Ascorbate peroxidase, **PPO:** Polyphenol oxidase.

* and ** Significantly at the probability level of %5 and %1, respectively. Mean comparison was performed by LSD method at 5% probability. Columns with similar letters did not differ significantly.

**Table S13.** The mean comparison of interaction effects of **d**ifferent levels of water deficit, year and yeast extract (Y) on evaluated traits of Cowpea

| **Ttraetment** | | | **PH** | **NN** | **NL** | **SD** | **PD** | **PL** | **PW20** | **GW100** | **NP** |
| --- | --- | --- | --- | --- | --- | --- | --- | --- | --- | --- | --- |
| **year** | **Irri** | **y** |  |  |  |  |  |  |  |  |  |
| **1** | **8** | **0** | 22.92±3.22 cd | 10.91±0.96 bcd | 1.05±0.21 b | 6.58±1.1 b | 6.94±0.9 a | 12.6±1.1 ab | 39.07±5.39 ab | 15.34±1.44 abc | 10.07±0.59 ab |
|  |  | **12** | 20.73±1.63 d | 10.66±0.84 cd | 1.01±0.13 b | 6.62±0.82 b | 7.26±0.37 a | 11.66±1.18 b | 40.07±4.69 a | 15.98±2.23 ab | 10.1±1.24 ab |
|  | **12** | **0** | 19.94±1.66 d | 10.35±0.57 d | 0.9±0.1 b | 6.98±0.61 b | 6.98±0.34 a | 11.25±1.28 b | 30.31±6.04de | 14.9±2.02 bcd | 7.98±0.64cde |
|  |  | **12** | 20.35±2.53 d | 10.48±0.44 cd | 0.77±0.13 b | 6.67±0.62 b | 7.02±0.95 a | 11.93±1.5 b | 37.79±7.03ab | 15.09±2.13 bc | 8.2±0.48 c |
|  | **16** | **0** | 20.80±1.18 d | 10.72±0.43 bcd | 2.09±3.39 b | 7±1.2 b | 6.93±0.38 a | 11.9±1.97 b | 35.04±8.28 bc | 16.99±3.77 a | 6.98±0.86 f |
|  |  | **12** | 21.68±1.15 d | 10.64±0.37 cd | 1.05±0.23 b | 84.14±231.32 a | 7.07±0.56 a | 11.25±0.37 b | 33.6±4.54 cd | 16.46±2.52 ab | 7.46±0.61 ef |
| **2** | **8** | **0** | 31.77±4.59 a | 11.52±0.64 abc | 6.11±8.61 a | 7.87±0.69 b | 6.91±0.09 a | 11.96±1.02 b | 27.09±2.6 ef | 12.98±1.47 e | 9.71±0.77 b |
|  |  | **12** | 33.05±8.54 a | 11.26±0.66abcd | 3.01±0.49 b | 7.53±0.9 b | 6.91±0.22 a | 11.73±0.77 b | 26.22±3.17 fg | 12.44±1.18 e | 10.45±1.04 a |
|  | **12** | **0** | 32.08±6.42 a | 11.15±0.46abcd | 3.04±0.74 b | 7.33±0.72 b | 6.95±0.29 a | 11.7±1.14 b | 25.71±3.62 fg | 13.18±0.96 de | 8.05±0.58 cd |
|  |  | **12** | 31.14±7.24 ab | 11.75±0.2 ab | 2.95±0.39 b | 7.16±0.65 b | 6.79±0.29 a | 11.58±0.86 b | 23.03±2.73 g | 13.48±2.73 cde | 8.38±0.53 c |
|  | **16** | **0** | 27±7.73 bc | 10.99±3.39 bcd | 2.66±0.89 b | 7.37±0.78 b | 7.36±1.73 a | 13.49±4.27 a | 24.85±7.05 fg | 12.43±1.69 e | 7.23±0.56 f |
|  |  | **12** | 30.50±3.45 ab | 12.12±0.16 a | 2.75±0.44 b | 7.24±0.65 b | 6.85±0.34 a | 11.48±1.09 b | 24.04±2.47 fg | 12.68±0.54 e | 7.47±0.61 def |

**Table S13** (continued).

| **Traetment** | | | **Chla** | **Chlb** | **Total Chl** | **Car** | **Prolin** | **Suger** | **Phenol** | **Protein** | **Yield** |
| --- | --- | --- | --- | --- | --- | --- | --- | --- | --- | --- | --- |
| **year** | **Irri** | **y** |  |  |  |  |  |  |  |  |  |
| **1** | **8** | **0** | 12.11±1.26 b | 6.05±0.68 b | 18.16±1.9 b | 13.18±0.62 c | 2.49±0.44 f | 3.23±0.43 f | 23.55±4.76 c | 137.55±22.2 f | 2245.54±408.4 a |
|  |  | **12** | 13.52±1.88 a | 6.82±1.35 a | 20.35±2.6 a | 14.14±1.92 ab | 2.96±0.64 e | 3.96±0.72 e | 28.47±5.17 bc | 180.24±29.61 de | 2205.44±349.34 a |
|  | **12** | **0** | 10.22±1.48 e | 4.41±0.77 de | 14.63±2.06 d | 10.88±1.05 e | 3.38±0.72 d | 4.56±0.59 d | 30.11±6.29 bc | 163.11±24.05 e | 1442.95±281.1 cd |
|  |  | **12** | 11.55±2.35 bc | 5.28±1.02 c | 16.84±3.24 c | 11.66±1.87 d | 3.83±0.82 c | 5.26±0.93 c | 37.22±6.77 bc | 213±52.36 bc | 1583.15±343.6 bc |
|  | **16** | **0** | 6.77±1.2 g | 2.92±0.78 f | 9.7±1.85 f | 7.77±0.97 f | 4.27±1.06 b | 5.74±0.73 b | 37.33±6.81 bc | 218±36.41 b | 1539.23±180.89 bcd |
|  |  | **12** | 8.77±2.78 f | 4.08±1.05 e | 12.86±3.69 e | 8.44±1.87 f | 4.95±1.45 a | 6.91±0.88 a | 44.55±5.7 ab | 276±65.37 a | 1497.97±200.28 bcd |
| **2** | **8** | **0** | 11.88±1.26 bc | 6.05±0.88 b | 17.94±1.84 b | 13.55±0.88 bc | 2.55±0.53 f | 3.27±0.45 f | 23.55±4.41 c | 138±23.68 f | 1704.34±254.98 b |
|  |  | **12** | 13.33±1.87 a | 6.61±1.05 a | 19.94±2.88 a | 14.55±1.94 a | 3±0.66 e | 4.05±0.7 e | 28.66±5.54 bc | 175.33±34.41 de | 1715.33±320.14 b |
|  | **12** | **0** | 10.55±1.33 de | 4.56±0.8 d | 15.12±2 d | 11.16±1.22 de | 3.39±0.73 d | 4.6±0.62 d | 30.88±5.29 bc | 163.66±22.66 e | 1332.27±219.57 cde |
|  |  | **12** | 11.11±2.26 cd | 5.21±1.1 c | 16.32±3.21 c | 11.66±1.65 d | 3.81±0.81 c | 5.32±0.85 c | 37.11±6.7 bc | 214.88±55.76 b | 1328.43±201.2 def |
|  | **16** | **0** | 6.77±1.48 g | 2.63±0.46 f | 9.41±1.91 f | 7.77±0.97 f | 4.3±1.07 b | 5.77±0.71 b | 61.44±75.84 a | 192.22±69.05 cd | 1079.63±426.17 f |
|  |  | **12** | 8.66±2.39 f | 4.33±1.31 de | 12.99±3.42 e | 8.33±1.87 f | 5.13±1.56 a | 6.9±0.96 a | 44.88±5.2 ab | 279.33±70.42 a | 1164.44±137.35 f |

**Table S13** (continued).

| **Traetment** | | | **Flavonoid** | **MDA** | **SOD** | **APX** | **GPX** | **CAT** | **PPO** | **LL** | | **LW** | **NGP** |
| --- | --- | --- | --- | --- | --- | --- | --- | --- | --- | --- | --- | --- | --- |
| **year** | **Irri** | **y** |  |  |  |  |  |  |  |  |  |  |  |
| **1** | **8** | **0** | 12±1.5 h | 6.12±0.63 efg | 0.033±0.003 g | 0.049±0.008 f | 0.04±0.004 f | 0.023±0.003 f | 0.027±0.005 b | | 6.54±0.6 bc | 4.71±0.57 b | 9.67±1.28 a |
|  |  | **12** | 14.86±2.1 g | 5.57±0.74 fg | 0.038±0.004 f | 0.059±0.009 e | 0.058±0.01 de | 0.028±0.007 e | 0.039±0.009 b | | 5.58±0.78 d | 3.94±0.53 b | 9.45±1.61 a |
|  | **12** | **0** | 17.33±1.8 f | 8.07±1.09 c | 0.041±0.004 ef | 0.068±0.01 d | 0.053±0.007 e | 0.099±0.1 a | 0.035±0.005 b | | 6.13±0.65 cd | 4.22±0.48 b | 8.13±1.19 a |
|  |  | **12** | 19.55±1.81 de | 6.85±1.13 de | 0.048±0.005 d | 0.092±0.02 b | 0.075±0.01 b | 0.034±0.005 c | 0.051±0.01 b | | 5.75±0.75 d | 3.81±0.56 b | 8.59±1.77 a |
|  | **16** | **0** | 25.33±2.59 b | 11.88±1.45 a | 0.053±0.004 c | 0.087±0.006 c | 0.069±0.008 c | 0.032±0.003 d | 0.044±0.005 b | | 6.26±0.63 cd | 4.41±0.47 b | 9.18±2.5 a |
|  |  | **12** | 30.44±4.12 a | 10.11±1.9 b | 0.061±0.008 a | 0.13±0.03 a | 0.106±0.03 a | 0.043±0.009 b | 0.064±0.01 b | | 5.78±0.64 d | 4.03±0.26b | 8.27±1.34 a |
| **2** | **8** | **0** | 11.88±1.53 h | 5.94±0.72 efg | 0.034±0.004 g | 0.051±0.008 f | 0.042±0.004 f | 0.023±0.003 f | 0.027±0.006 b | | 7.73±0.92 a | 5.59±0.73 ab | 9.07±1.2 a |
|  |  | **12** | 15.11±2.2 g | 5.27±0.54 g | 0.038±0.005 f | 0.06±0.01 e | 0.059±0.01 d | 0.029±0.008 e | 0.04±0.009 b | | 6.6±0.76 bc | 4.52±0.58 b | 8.76±0.89 a |
|  | **12** | **0** | 17.88±1.9 ef | 7.88±1.61 cd | 0.042±0.005 e | 0.07±0.009 d | 0.054±0.007 e | 0.027±0.003 e | 0.035±0.006 b | | 7.2±0.59 ab | 5.2±0.4 b | 8.36±1.51 a |
|  |  | **12** | 19.88±2.08 d | 6.52±1.16 ef | 0.05±0.007 cd | 0.093±0.02 b | 0.077±0.01 b | 0.034±0.005 cd | 0.053±0.01 b | | 6.06±0.5 cd | 3.9±0.6 b | 7.95±1.02 a |
|  | **16** | **0** | 23.44±5.65 c | 10.78±4.15 ab | 0.057±0.01 b | 0.086±0.006 c | 0.065±0.01 c | 0.034±0.008 c | 0.706±1.98 a | | 7.04±1.31 b | 8.31±10.21 a | 143.66±404.77 a |
|  |  | **12** | 30.88±4.13 a | 9.88±1.83 b | 0.061±0.007 a | 0.092±0.06 b | 0.107±0.03 a | 0.044±0.009 b | 0.064±0.01 b | | 5.7±0.67 d | 3.44±0.59 b | 8.2±0.84 a |

**Irri:** Different levels of water deficit**, Y:** yeast extract, **PH:** Plant height, **NN:** Number of nodes, **NL:** Distance internode, **SD:** Stem diameter, **PD:** PPO diameter, **PL:** PPO length, **PW 20:** Weight 20 PPOs, **GW 100:** Weight 100 grains, **NP:** Number of PPOs , **LL:** Leaf length , **Yi:** Yield, **LW:** Leaf width, **NGP:** Number of seeds in PPO, **Chla:** Chlorophyll a, **Chlb:** Chlorophyll b, **ChlT:** Total chlorophyll, **Car:** Carotenoid, , **MDA**: Malondialdehyde, **CAT:** Catalase, **SOD:** Superoxide dismutase, **GPX:** Guaiacol peroxidase, **APX:** Ascorbate peroxidase, **PPO:** Polyphenol oxidase.

* and ** Significantly at the probability level of %5 and %1, respectively. Mean comparison was performed by LSD method at 5% probability. Columns with similar letters did not differ significantly.

**Table S14.** The mean comparison of interaction effects of year, 24-epibrassinolide (B) and yeast extract (Y) on evaluated traits of Cowpea

| **Traetment** | | | **PH** | **NN** | **NL** | **SD** | **PD** | **PL** | **PW20** | **GW100** | **NP** |
| --- | --- | --- | --- | --- | --- | --- | --- | --- | --- | --- | --- |
| **year** | **B** | **y** |  |  |  |  |  |  |  |  |  |
| **1** | **0** | **0** | 21.89±1.99 c | 10.83±0.61abcd | 0.9±0.1 b | 7.15±1.02 b | 6.69±0.51 b | 12.06±2.15 ab | 29.98±7.64 b | 16.44±3.3 a | 8.45±1.12 bcde |
|  |  | **12** | 20.93±2.05 c | 10.65±0.46bcd | 0.94±0.24 b | 93.49±245.5a | 7.04±1.06 ab | 11.46±1.01 ab | 35.9±4.72 a | 15.77±2.49 ab | 7.8±0.69 f |
|  | **5** | **0** | 20.65±2.26 c | 10.68±0.69bcd | 2.11±3.38 b | 6.76±0.79 b | 7.12±0.36 ab | 11.92±1.16 ab | 36.94±7.2 a | 14.5±1.71 bc | 8.06±1.77 ef |
|  |  | **12** | 21.03±1.26 c | 10.71±0.43bcd | 0.96±0.17 b | 7±0.7 b | 7.11±0.35 ab | 11.88±0.92 ab | 36.04±6.41 a | 16.09±2.02 ab | 8.75±1.5 abcd |
|  | **10** | **0** | 21.12±3.13 c | 10.48±0.83 cd | 1.04±0.27 b | 6.66±1.15 b | 7.04±0.75 ab | 11.77±1.3 ab | 37.45±5.24 a | 16.29±2.59 ab | 8.53±1.59 bcde |
|  |  | **12** | 20.8±2.41 c | 10.42±0.74 d | 0.93±0.22 b | 6.94±1.04 b | 7.2±0.52 ab | 11.49±1.41 ab | 39.53±6.65 a | 15.67±2.59 ab | 9.02±1.48 ab |
| **2** | **0** | **0** | 29.69±3.69ab | 11.78±0.42 a | 2.67±0.78 b | 8±0.71 b | 6.86±0.38 ab | 11.9±0.57 ab | 26.56±2.31bc | 13.13±1 cd | 8.76±1.21 abcd |
|  |  | **12** | 31.55±5.56ab | 11.82±0.49 a | 2.93±0.38 b | 7.31±0.98 b | 6.98±0.19 ab | 11.88±0.8 ab | 24.1±2.13 c | 12.99±0.9 cd | 8.31±1.23 cdef |
|  | **5** | **0** | 33.61±6.07 a | 11.51±0.62abc | 5.94±8.65 a | 7.32±0.67 b | 6.89±0.2 ab | 12.25±0.83 ab | 26.7±2.02 bc | 12.79±0.59 cd | 8.02±1.19 ef |
|  |  | **12** | 32.43±7.38 a | 11.68±0.47 ab | 2.95±0.48 b | 4.32±0.65 b | 6.77±0.26 b | 11.57±0.85 ab | 24.16±2.97 c | 12.19±2.04 d | 8.82±1.75 abc |
|  | **10** | **0** | 27.55±8.29 b | 10.36±3.23 d | 3.19±1.02 b | 7.25±0.68 b | 7.46±1.69 a | 12.99±4.57 a | 24.38±7.53 c | 12.66±2.19 cd | 8.21±1.26 def |
|  |  | **12** | 30.7±7.36 ab | 11.63±0.65 ab | 2.83±0.49 b | 7.29±0.6 b | 6.8±0.35 b | 11.34±1 b | 25.03±3.95 c | 13.43±1.98 cd | 9.18±1.39 a |

**Table S14** (continued).

| **Traetment** | | | **Chla** | **Chlb** | **Total Chl** | **Car** | **Prolin** | **Suger** | **Phenol** | **Protein** | **Yield** |
| --- | --- | --- | --- | --- | --- | --- | --- | --- | --- | --- | --- |
| **year** | **B** | **y** |  |  |  |  |  |  |  |  |  |
| **1** | **0** | **0** | 8.55±2.45 e | 3.76±1.42 d | 12.32±3.83 e | 9.88±2.57 fg | 2.53±0.44 e | 3.86±0.92 f | 24.22±5.3 b | 145.88±31.01 e | 1711.03±378.21 ab |
|  |  | **12** | 8.41±2.69 e | 4.35±1.07 d | 12.76±3.7 e | 9.25±2.26 g | 2.72±0.45de | 4.4±1.2 e | 29.92±6.91 b | 170.13±28.32 cd | 1530.89±307.35 bc |
|  | **5** | **0** | 9.55±2.45 d | 4.33±1.49 d | 13.88±3.88 d | 10.63±2.5 de | 3.36±0.82 c | 4.52±1.11 e | 29.22±5.58 b | 166.33±32.65cde | 1702.93±567.68 ab |
|  |  | **12** | 12±2 b | 5.4±1.46 b | 17.4±3.37 b | 11.66±2.91 b | 4.11±1.02 b | 5.38±1.29 b | 36.88±6.56 b | 213.11±42.14 b | 1888.12±564.79 a |
|  | **10** | **0** | 11±2.5 c | 5.28±1.26 b | 16.28±3.63 c | 11.33±2.23bcd | 4.42±1.07 b | 5.15±1.25 d | 37.55±7.29 b | 206.44±42.12 b | 1813.76±486.57 a |
|  |  | **12** | 13.44±1.94 a | 6.44±1.33 a | 19.88±2.8 a | 13.33±2.59 a | 4.91±1.2 a | 6.36±1.35 a | 43.44±7.55ab | 282±62.56 a | 1867.55±432.69 a |
| **2** | **0** | **0** | 8.44±2.45 e | 3.77±1.42 d | 12.22±3.79 e | 10.05±2.74 ef | 2.52±0.44 e | 3.87±0.93 f | 24.77±5.19 b | 147.88±32.36 de | 1518.87±303.8 bc |
|  |  | **12** | 8.33±2.39 e | 4.17±0.99cd | 12.5±3.21 e | 9.44±2.65 fg | 2.81±0.52 d | 4.52±1.12 e | 29.88±7.45 b | 169.33±31.9 cd | 1319.39±144 cd |
|  | **5** | **0** | 9.66±2.5 d | 4.33±1.56 d | 14±3.89 d | 10.88±2.71 cd | 3.42±0.86 c | 4.56±1.12 e | 29.33±5.43 b | 165.33±32.83cde | 1351.56±267.13 cd |
|  |  | **12** | 12±2.17 b | 5.48±1.29 b | 17.48±3.4 b | 11.77±2.81 b | 4.13±1.01 b | 5.36±1.25bc | 37.88±6.6 b | 210.77±40.93 b | 1368.77±351.33 cd |
|  | **10** | **0** | 11.11±2.26 c | 5.14±1.65 b | 16.25±3.85 c | 11.55±2.45 bc | 4.3±0.99 b | 5.2±1.21cd | 61.77±75.42a | 180.66±69.04 c | 1245.81±557.44 d |
|  |  | **12** | 12.77±1.85ab | 6.5±0.93 a | 19.27±2.63 a | 13.33±2.87 a | 5±1.44 a | 6.4±1.37 a | 42.88±7.44ab | 289.44±66.21 a | 1520.03±416 bc |

**Table S14** (continued).

| **Ttraetment** | | | **Flavonoid** | **MDA** | **SOD** | **APX** | **GPX** | **CAT** | **PPO** | **LL** | **LW** | **NGP** |
| --- | --- | --- | --- | --- | --- | --- | --- | --- | --- | --- | --- | --- |
| **year** | **B** | **y** |  |  |  |  |  |  |  |  |  |  |
| **1** | **0** | **0** | 16.22±5.33 f | 9.66±3 ab | 0.038±0.008 ef | 0.058±0.01 f | 0.046±0.01 e | 0.096±0.1 a | 0.03±0.007 b | 6.54±0.54 bc | 4.53±0.57 b | 8.49±2.13 b |
|  |  | **12** | 18.75±5.83 cd | 8.61±2.73abcd | 0.042±0.008 d | 0.066±0.01 e | 0.056±0.01 cd | 0.029±0.006 d | 0.039±0.01 b | 5.73±0.5 de | 3.91±0.33 b | 8.51±1.78 b |
|  | **5** | **0** | 18.11±6 de | 8.83±2.62 abc | 0.042±0.008 de | 0.069±0.01 d | 0.055±0.01 d | 0.026±0.003 ef | 0.035±0.007 b | 6.33±0.84bcd | 4.49±0.62 b | 9.75±1.9 b |
|  |  | **12** | 21.33±6.83 b | 7.73±2.09 cd | 0.049±0.009 bc | 0.093±0.02 b | 0.075±0.01 b | 0.033±0.005 c | 0.051±0.01 b | 5.49±0.82 e | 3.82±0.58 b | 8.89±1.71 b |
|  | **10** | **0** | 20.33±6.28 bc | 7.58±2.17 d | 0.047±0.009 c | 0.077±0.01 c | 0.061±0.01 c | 0.032±0.004 c | 0.042±0.007 b | 6.16±0.48bcde | 4.32±0.42 b | 8.74±1.23 b |
|  |  | **12** | 24.77±8.15 a | 6.2±1.47 ef | 0.056±0.01 a | 0.122±0.04 a | 0.108±0.03 a | 0.044±0.008 b | 0.064±0.01 b | 5.9±0.74 cde | 4.05±0.45 b | 8.91±1.55 b |
| **2** | **0** | **0** | 16.44±5.27 ef | 9.77±2.86 a | 0.038±0.008 f | 0.061±0.01 f | 0.047±0.01 e | 0.024±0.004 f | 0.03±0.008 b | 7.58±0.88 a | 5.52±0.74ab | 8.82±1.2 b |
|  |  | **12** | 19.22±6.24 cd | 8.43±2.57 cd | 0.042±0.009 d | 0.066±0.01 e | 0.058±0.01 cd | 0.029±0.006 d | 0.04±0.009 b | 6.71±0.62 b | 4.37±0.53 b | 8.23±0.75 b |
|  | **5** | **0** | 18.11±6.13 de | 8.5±2.8 bcd | 0.042±0.008 d | 0.071±0.01 d | 0.055±0.01 d | 0.026±0.004 de | 0.034±0.007 b | 7.84±0.58 a | 5.31±0.52 b | 8.76±0.97 b |
|  |  | **12** | 21.44±6.67 b | 7.38±2.34 de | 0.05±0.008 bc | 0.055±0.03 g | 0.076±0.01 b | 0.033±0.005 c | 0.051±0.01 b | 5.68±0.43 de | 3.66±0.58 b | 8.5±0.93 b |
|  | **10** | **0** | 18.66±6.68 cd | 6.34±3.3 ef | 0.052±0.01 b | 0.075±0.01 c | 0.058±0.01 cd | 0.034±0.008 c | 0.703±1.98 a | 6.45±1.02 bc | 8.27±10.21a | 143.51±404.9 a |
|  |  | **12** | 25.22±8.33 a | 5.86±1.4 f | 0.057±0.01 a | 0.124±0.04 a | 0.109±0.03 a | 0.045±0.008 b | 0.065±0.01 b | 5.96±0.74 cde | 3.82±0.88 b | 8.18±1.19 b |

**B:** 24-epibrassinolide**, Y:** yeast extract, **PH:** Plant height, **NN:** Number of nodes, **NL:** Distance internode, **SD:** Stem diameter, **PD:** PPO diameter, **PL:** PPO length, **PW 20:** Weight 20 PPOs, **GW 100:** Weight 100 grains, **NP:** Number of PPOs , **LL:** Leaf length , **Yi:** Yield, **LW:** Leaf width, **NGP:** Number of seeds in PPO, **Chla:** Chlorophyll a, **Chlb:** Chlorophyll b, **ChlT:** Total chlorophyll, **Car:** Carotenoid, , **MDA**: Malondialdehyde, **CAT:** Catalase, **SOD:** Superoxide dismutase, **GPX:** Guaiacol peroxidase, **APX:** Ascorbate peroxidase, **PPO:** Polyphenol oxidase.

* and ** Significantly at the probability level of %5 and %1, respectively. Mean comparison was performed by LSD method at 5% probability. Columns with similar letters did not differ significantly.

**Table S15.** The mean comparison of interaction effects of different levels of water deficit, 24-epibrassinolide (B) and yeast extract (Y) on evaluated traits of Cowpea

| **Traetment** | | | **PH** | **NN** | **NL1** | **SD** | **PD** | **PL** | **PW20** | **GW100** | **NP** |
| --- | --- | --- | --- | --- | --- | --- | --- | --- | --- | --- | --- |
| **Irri** | **B** | **y** |  |  |  |  |  |  |  |  |  |
| **8** | **0** | **0** | 27.54±5.76 a | 11.72±0.56 a | 1.87±1.1 b | 7.69±1.05 b | 6.69±0.53 b | 12.47±0.77 ab | 30.88±5.89 abcd | 13.58±1.49 ab | 10.03±071 bc |
|  |  | **12** | 27.43±8.81 a | 11.18±0.83 | 2±0.96 b | 6.86±1.31 b | 7.2±0.45 ab | 11.72±1 ab | 32.51±9.91 abcd | 13.93±1.78 ab | 8.94±1.17 de |
|  | **5** | **0** | 27.09±6.49 a | 10.81±0.97abc | 6.28±11.15 a | 7.04±1.01 b | 7.17±0.32ab | 12.34±1.32 ab | 35.14±9.66 a | 14.15±1.76 ab | 9.55±0.79 cd |
|  |  | **12** | 28.32±10.82 a | 11.11±0.36abc | 2.05±1.27 b | 7.64±0.44 b | 7.04±0.37ab | 12.06±0.32 ab | 34.12±10.36 ab | 14.05±3.56 ab | 10.73±0.66ab |
|  | **10** | **0** | 27.41±6.74 a | 11.11±0.84abc | 2.59±1.62 b | 6.93±1.29 b | 6.92±0.88 b | 12.03±1.23 ab | 33.15±6.83 abc | 14.74±2.39 ab | 10.1±0.55 bc |
|  |  | **12** | 24.92±7.71 ab | 10.6±0.99 abc | 1.98±1.21 b | 6.72±0.83 b | 7.01±0.24ab | 11.3±1.29 b | 32.8±4.07 abcd | 14.66±2.16 ab | 10.88±0.49 a |
| **12** | **0** | **0** | 25.7±5.75 ab | 11.03±0.61abc | 2.01±1.43 b | 7.31±0.88 b | 6.97±0.33 b | 10.95±1.05 b | 25.04±3.17 e | 15.24±1.6 ab | 8.06±0.38fgh |
|  |  | **12** | 24.91±7.25 ab | 11.2±0.67 ab | 1.78±1.27 b | 7.1±0.91 b | 6.71±1.09 b | 11.48±1.09 b | 28.87±6.62 cde | 13.85±1.86 ab | 8.05±0.21fgh |
|  | **5** | **0** | 27.59±10.52 a | 10.94±0.38abc | 2.1±1.4 b | 6.9±0.52 b | 6.85±0.32 b | 11.95±0.78 ab | 28.11±3.6 de | 12.98±1.33 b | 8±0.89fgh |
|  |  | **12** | 25.56±7.98 ab | 11.15±0.76abc | 2.04±1.28 b | 6.49±0.28 b | 6.93±0.42 b | 11.81±1.06 ab | 28.32±6.24 cde | 14.27±3.01 ab | 8.28±0.72efg |
|  | **10** | **0** | 24.75±7.35 ab | 10.28±0.71 bc | 1.8±0.94 b | 7.25±0.62 b | 7.07±0.29ab | 11.53±1.6 b | 30.88±7.13 abcd | 13.89±1.8 ab | 8±0.51 fgh |
|  |  | **12** | 26.76±8.97 a | 11±0.88 abc | 1.76±1.09 b | 7.15±0.51 b | 7.08±0.43ab | 11.98±1.56 ab | 34.04±13.66 ab | 14.75±2.92 ab | 8.55±0.39 ef |
| **16** | **0** | **0** | 24.12±3.09 ab | 11.18±0.84 ab | 1.47±0.61 b | 7.27±1.06 b | 6.68±0.46 b | 12.52±2.09 ab | 28.9±6.82 cde | 15.52±4.63 a | 7.73±0.33ghij |
|  |  | **12** | 26.38±5.24 a | 11.33±0.88 ab | 2.03±1.13 b | 122.25±238.5a | 7.12±0.3 ab | 11.8±0.76 ab | 28.61±4.39 cde | 15.36±3 a | 7.18±0.54 ijk |
|  | **5** | **0** | 26.71±8.06 a | 11.53±0.75 ab | 3.7±3.78 ab | 7.17±0.82 b | 6.99±0.23ab | 11.97±0.95 ab | 32.21±6.92 abcd | 13.81±1.45 ab | 6.58±0.8 k |
|  |  | **12** | 26.31±4.6 a | 11.33±0.87 ab | 1.78±0.82 b | 7.35±0.64 b | 6.85±0.28 b | 11.31±1.03 b | 27.86±5.6 de | 14.12±2.26 ab | 7.35±0.5 hij |
|  | **10** | **0** | 20.86±6.11 b | 9.86±3.95 c | 1.96±1.41 b | 6.67±0.97 b | 7.76±2.04 a | 13.58±5.54 a | 28.72±13.38 cde | 14.79±4.55 ab | 7.01±0.44 jk |
|  |  | **12** | 25.57±6.49 ab | 11.48±0.81 ab | 1.9±1 b | 7.48±1.06 b | 6.91±0.71 b | 10.97±0.33 b | 30±8.46 bcde | 14.23±2.86 ab | 7.88±0.58fghi |

**Table S15** (continued).

| **Traetment** | | | **Flavonoid** | **Chla** | **Chlb** | **Total Chl** | **Car** | **Prolin** | **Suger** | **Phenol** | **Protein** | **Yield** |
| --- | --- | --- | --- | --- | --- | --- | --- | --- | --- | --- | --- | --- |
| **Irri** | **B** | **y** |  |  |  |  |  |  |  |  |  |  |
| **8** | **0** | **0** | 10.66±0.81 k | 10.83±0.75 f | 5.33±0.51 f | 16.16±0.98 f | 12.83±0.75de | 2.02±0.04 n | 2.8±0.19 m | 18.5±1.04 d | 117.5±2.07 i | 1984.88±272.83 a |
|  |  | **12** | 12.8±0.83 ijk | 11.11±0.83def | 5.57±0.54ef | 16.69±1.14ef | 12.05±0.44efg | 2.17±0.16mn | 3.21±0.16 l | 21.88±1.09cd | 140.2±6.18 ghi | 1621.74±337.09 b |
|  | **5** | **0** | 11.5±0.54 jk | 12±0.89 d | 6.08±0.66de | 18.08±1.02 d | 13.45±0.5 cd | 2.41±0.12 lm | 3.21±0.18 l | 23.33±0.81cd | 128.33±1.96 i | 1953.58±565.88 a |
|  |  | **12** | 14.66±0.81 hi | 14.16±0.75 ab | 7±0.63 b | 21.66±0.75 b | 14.5±0.54 b | 3.11±0.14 ij | 3.97±0.04 j | 29.83±1.32bcd | 173.83±7.13 ef | 2106.77±513.43 a |
|  | **10** | **0** | 13.66±0.81 hij | 13.16±0.75 c | 6.75±0.3 bc | 19.91±0.91 c | 13.83±0.75 bc | 3.12±0.15 hij | 3.74±0.18 k | 28.83±1.16bcd | 167.5±4.23 fg | 1986.37±488.89 a |
|  |  | **12** | 17.5±0.54 fg | 15±1.09 a | 7.58±1.02 a | 22.58±0.66 a | 16.5±0.83 a | 3.67±0.12 fg | 4.85±0.18gh | 34±1.26 bcd | 213.33±6.05cd | 2152.63±330.79 a |
| **12** | **0** | **0** | 15.66±0.81 gh | 9.16±0.75hi | 3.85±0.23gh | 13.01±0.82h | 10.08±0.91ij | 2.58±0.12kl | 3.87±0.12jk | 24.66±1.63cd | 135.83±4.35 hi | 1396.89±154.73 bc |
|  |  | **12** | 17.66±0.81 fg | 8.5±0.83ij | 4.08±0.27g | 12.58±0.81hi | 9.5±0.54jk | 2.84±0.11jk | 4.29±0.17i | 29.33±1.03bcd | 162±4.33 fgh | 1330.19±147.29 bc |
|  | **5** | **0** | 17.5±0.54 fg | 10.16±0.75fg | 4.2±0.4g | 14.36±0.85g | 11.16±0.98gh | 3.43±0.16gh | 4.65±0.11h | 28.66±0.81bcd | 166±5.83 fg | 1295.58±180.92 bc |
|  |  | **12** | 19.66±0.81 ef | 12.16±0.75d | 5.33±0.51f | 17.5±1.04de | 12.5±0.54ef | 3.91±0.09ef | 5.25±0.13f | 37.5±1.04 bcd | 197±4.28 de | 1462.18±420.42 b |
|  | **10** | **0** | 19.66±0.81 ef | 11.83±0.98 de | 5.41±0.41 f | 17.25±0.92def | 11.83±0.75 fg | 4.2±0.07 de | 5.23±0.2 f | 38.16±1.16bcd | 188.33±5.39def | 1470.36±373.4 b |
|  |  | **12** | 21.83±0.75 de | 13.33±0.81 bc | 6.33±0.51cd | 19.66±0.51 c | 13±0.89 cde | 4.7±0.2 c | 6.33±0.07 d | 44.66±1.63 bc | 282.83±7.19 b | 1575±278.54 b |
| **16** | **0** | **0** | 22.66±1.21 d | 5.5±0.54 l | 2.13±0.2 k | 7.63±0.42 k | 7±0.89 no | 3.03±0.05 ij | 4.93±0.06 g | 30.33±1.36 bcd | 187.33±7 def | 1463.08±249.8 b |
|  |  | **12** | 26.5±1.04 c | 5.5±0.54 l | 3.12±0.26 ij | 8.62±0.69 jk | 6.5±0.54 o | 3.29±0.16 hi | 5.89±0.09 e | 38.5±0.83 bcd | 207±7.23 cd | 1323.48±134.2 bc |
|  | **5** | **0** | 25.33±1.36 c | 6.66±0.81 k | 2.71±0.23 j | 9.38±1 j | 7.66±0.51 mn | 4.31±0.15 d | 5.76±0.21 e | 35.83±1.16 bcd | 203.16±5.87 cd | 1332.58±244.88 bc |
|  |  | **12** | 29.83±1.16 b | 9.66±0.81 gh | 4±0.21 gh | 13.66±0.92 gh | 8.16±0.75 lm | 5.33±0.51 b | 6.88±0.2 b | 44.83±1.32 bc | 265±8.36 b | 1316.39±292.7 bc |
|  | **10** | **0** | 25.1±7.526 c | 8.16±0.75 j | 3.48±0.45hi | 11.65±0.73 i | 8.66±0.51 kl | 5.49±0.17 b | 6.56±0.15 c | 82±88.19 a | 224.83±97.03 c | 1132.62±586.65 c |
|  |  | **12** | 35.66±0.81 a | 11±0.63 ef | 5.5±0.54 f | 16.5±1.04 ef | 10.5±0.83 hi | 6.5±0.83 a | 7.95±0.34 a | 50.83±1.32 b | 361±11.91 a | 1353.74±299.74 bc |

**Table S15** (continued).

| **Traetment** | | | **MDA** | **SOD** | **APX** | **GPX** | **CAT** | **PPO** | **LL** | **LW** | **NGP** |
| --- | --- | --- | --- | --- | --- | --- | --- | --- | --- | --- | --- |
| **Irri** | **B** | **y** |  |  |  |  |  |  |  |  |  |
| **8** | **0** | **0** | 6.66±0.51 ef | 0.029±0.002 j | 0.04±0.001 l | 0.036±0.001 h | 0.019±0.001 k | 0.021±0.001 b | 7.43±1.09 ab | 5.62±0.75 b | 9.74±1.09 b |
|  |  | **12** | 5.9±0.71 fg | 0.031±0.001 j | 0.047±0.001 k | 0.043±0.002 g | 0.021±0.0005 k | 0.028±0.002 b | 6.41±1.1 cde | 4.28±0.63 b | 8.84±1.31 b |
|  | **5** | **0** | 6±0.31 fg | 0.033±0.002 ij | 0.052±0.003 j | 0.041±0.002gh | 0.022±0.0005 k | 0.026±0.001 b | 7.55±0.81 a | 5.39±0.5 b | 9.47±1.17 b |
|  |  | **12** | 5.51±0.44 fg | 0.041±0.001 fgh | 0.062±0.001 h | 0.055±0.004 ef | 0.027±0.001 ij | 0.039±0.001 b | 5.7±0.48 ef | 4.02±0.3 b | 9.44±1.61 b |
|  | **10** | **0** | 5.43±0.49 fg | 0.038±0.001 gh | 0.059±0.002 hi | 0.046±0.001 g | 0.027±0.0009 ij | 0.034±0.003 b | 6.43±0.67 cde | 4.44±0.58 b | 8.89±1.5 b |
|  |  | **12** | 4.86±0.19 g | 0.042±0.001 fg | 0.07±0.001 g | 0.077±0.002 c | 0.038±0.001 cd | 0.05±0.002 b | 6.16±1.03 def | 4.39±0.8 b | 9.04±1.15 b |
| **12** | **0** | **0** | 9.33±1.03 bc | 0.037±0.0009 hi | 0.057±0.003 i | 0.045±0.001 g | 0.132±0.1 a | 0.028±0.001 b | 6.99±0.84 abc | 4.99±0.76 b | 7.65±1.19 b |
|  |  | **12** | 7.83±0.19 de | 0.042±0.001 f | 0.062±0.001 h | 0.056±0.002 ef | 0.029±0.0009 ghi | 0.039±0.002 b | 6.15±0.67 def | 4.16±0.5 b | 8.06±1.27 b |
|  | **5** | **0** | 8±0.63 cde | 0.041±0.001 fgh | 0.072±0.002 g | 0.054±0.003 f | 0.026±0.0007 j | 0.035±0.001 b | 6.51±1.04 cde | 4.45±0.65 b | 8.36±0.85 b |
|  |  | **12** | 6.83±0.75 ef | 0.047±0.001 e | 0.09±0.002 c | 0.075±0.002cd | 0.033±0.001 ef | 0.051±0.001 b | 5.73±0.88 ef | 3.66±0.73 b | 8.21±1.2 b |
|  | **10** | **0** | 6.61±0.49 ef | 0.048±0.001 e | 0.078±0.001 f | 0.061±0.002 e | 0.032±0.001 fg | 0.041±0.001 b | 6.5±0.52 cde | 4.69±0.53 b | 8.73±1.25 b |
|  |  | **12** | 5.4±0.37 fg | 0.057±0.002 c | 0.127±0.004 b | 0.097±0.002 b | 0.041±0.001 c | 0.065±0.003 b | 5.84±0.23 def | 3.74±0.38 b | 8.55±1.97 b |
| **16** | **0** | **0** | 13.16±0.75 a | 0.048±0.001 de | 0.082±0.002 e | 0.059±0.001 ef | 0.029±0.0008 hij | 0.04±0.001 b | 6.63±0.76 bcd | 4.47±0.57 b | 8.57±2.11 b |
|  |  | **12** | 11.83±0.75 a | 0.052±0.001 d | 0.089±0.002 cd | 0.072±0.002cd | 0.036±0.001 de | 0.052±0.001 b | 6.09±0.44 def | 3.98±0.26 b | 8.21±1.4 b |
|  | **5** | **0** | 12±0.89 a | 0.052±0.001 d | 0.086±0.002 d | 0.07±0.002 d | 0.031±0.001 fgh | 0.043±0.003 b | 7.2±1.16 abc | 4.86±0.65 b | 9.93±2.13 b |
|  |  | **12** | 10.33±0.51 b | 0.06±0.001 bc | 0.07±0.06 g | 0.096±0.002 b | 0.039±0.001 c | 0.063±0.002 b | 5.34±0.54 f | 3.54±0.57 b | 8.43±1.08 b |
|  | **10** | **0** | 8.84±4.33 cd | 0.064±0.01 b | 0.091±0.008 c | 0.071±0.01 d | 0.04±0.006 c | 1.04±2.42 a | 6.13±1.15 def | 9.75±12.6 a | 210.76±495.9 a |
|  |  | **12** | 7.83±7.52 de | 0.07±0.002 a | 0.172±0.004 a | 0.15±0.006 a | 0.055±0.001 b | 0.077±0.003 b | 5.79±0.75 ef | 3.68±0.68 b | 8.06±0.91 b |

**Irri:** Different levels of water deficit**, B:** 24-epibrassinolide**, Y:** yeast extract, **PH:** Plant height, **NN:** Number of nodes, **NL:** Distance between internode, **SD:** Stem diameter, **PD:** PPO diameter, **PL:** PPO length, **PW 20:** Weight 20 PPOs, **GW 100:** Weight 100 grains, **NP:** Number of PPOs , **LL:** Leaf length , **Yi:** Yield, **LW:** Leaf width, **NGP:** Number of seeds in PPO, **Chla:** Chlorophyll a, **Chlb:** Chlorophyll b, **ChlT:** Total chlorophyll, **Car:** Carotenoid, , **MDA**: Malondialdehyde, **CAT:** Catalase, **SOD:** Superoxide dismutase, **GPX:** Guaiacol peroxidase, **APX:** Ascorbate peroxidase, **PPO:** Polyphenol oxidase.

* and ** Significantly at the probability level of %5 and %1, respectively. Mean comparison was performed by LSD method at 5% probability. Columns with similar letters did not differ significantly.

**Table S16.** The mean comparison of interaction effects of different levels of water deficit, 24-epibrassinolide (B), yeast extract (Y) and year on evaluated traits of Cowpea

| **Traetment** | | | | **PH** | **NN** | **NL** | **SD** | **PD** | **PL** | **PW20** | **GW100** | **NP** |
| --- | --- | --- | --- | --- | --- | --- | --- | --- | --- | --- | --- | --- |
| **year** | **Irri** | **B** | **y** |  |  |  |  |  |  |  |  |  |
| **1** | **8** | **0** | **0** | 23.55±1.61 cdef | 11.38±0.65abcd | 0.91±0.17 b | 7.37±1.12 b | 6.45±0.74 b | 12.91±0.09 b | 34.86±4.75defg | 14.55±1.63 c-i | 9.9±0.36bcd |
|  |  |  | **12** | 22.63±2.16 def | 10.76±0.84 a-e | 1.17±0.02 b | 6.26±0.79 b | 7.42±0.53 b | 12.27±0.94 bc | 41.22±3.9abcd | 15.27±1.65 a-j | 8.34±0.28fghi |
|  |  | **5** | **0** | 21.59±3.42 ef | 10.52±1.29 a-e | 1.09±0.21 b | 6.42±0.84 b | 7.38±0.33 b | 12.2±2.06 bc | 43.2±6.16 ab | 15.65±0.97 a-h | 9.9±0.79 bcd |
|  |  |  | **12** | 20.36±0.81 ef | 11.1±0.55 abcd | 0.93±0.06 b | 7.48±0.6 b | 7.33±0.29 b | 12.3±0.17 bc | 42.88±5.61abc | 16.83±2.8abcd | 10.46±0.92abc |
|  |  | **10** | **0** | 23.62 ±4.83cdef | 10.83±1.04abcd | 1.15±0.24 b | 6±1.25 b | 6.99±1.38 b | 12.69±0.48 bc | 39±1.95 a-e | 15.82±1.82 a-g | 10.43±0.6 abc |
|  |  |  | **12** | 19.83±1.17 ef | 10.14±1.02 cde | 0.93±0.13 b | 6.11±0.46 b | 7.03±0.22 b | 10.39±0.87 bc | 36.12±0.82 c-g | 15.83±2.61 a-g | 10.93±0.15 a |
|  | **12** | **0** | **0** | 20.6±1.83 ef | 10.63±0.45 a-e | 0.87±0.03 b | 6.78±0.5 b | 6.75±0.3 b | 10.3±1.13 bc | 23.5±3.94 klm | 16.22±1.7 a-e | 7.83±0.28 g-l |
|  |  |  | **12** | 18.96±0.64 f | 10.63±0.32 a-e | 0.66±0.14 b | 6.63±0.96 b | 6.58±1.71 b | 10.83±1.04bc | 34.4±1.7 d-h | 14.55±2.21 c-i | 7.9±0.1 g-k |
|  |  | **5** | **0** | 19.68±2.25 ef | 10.61±0.12 a-e | 0.85±0.09 b | 6.95±0.74 b | 6.94±0.38 b | 11.65±0.05 bc | 31±0.09 f-j | 13.42±1.86 e-k | 8±1 g-k |
|  |  |  | **12** | 20.5±0.79 ef | 10.48±0.14 a-e | 0.89±0.09 b | 6.47±0.44 b | 7.17±0.2 b | 12.02±1.61 bc | 33.06±4.6 e-i | 15.5±2.09 a-i | 8.43±0.81 fgh |
|  |  | **10** | **0** | 19.55±1.27 ef | 9.82±0.67 de | 0.99±0.12 b | 7.21±0.73 b | 7.26±0.21 b | 11.82±1.79 bc | 36.43±2.05 b-g | 15.04±2.04 b-j | 8.13±0.7 ghij |
|  |  |  | **12** | 21.59±4.41 ef | 10.33±0.77 b-e | 0.77±0.05 b | 6.9±0.52 b | 7.33±0.48 b | 12.95±1.41 b | 45.83±6.52 a | 15.22±2.86 b-j | 8.26±0.25 fghi |
|  | **16** | **0** | **0** | 21.52±1.77 ef | 10.5±0.45 a-e | 0.91±0.09 b | 7.35±1.53 b | 6.89±0.52 b | 12.97±3.21 b | 31.58±9.65 f-j | 18.54±5.09 a | 7.63±0.4 h-l |
|  |  |  | **12** | 21.83±1.46 ef | 10.57±0.46 a-e | 1±0.12 b | 237.58±401.a | 7.12±0.45 b | 11.26±0.63 bc | 32±3.33 e-j | 17.48±2.96 abc | 7.16±0.76 jklm |
|  |  | **5** | **0** | 20.68±1.01 ef | 10.9±0.36 abcd | 4.38±5.84 b | 6.9±1.01 b | 7.04±0.33 b | 11.92±0.99 bc | 36.63±7.61 b-f | 14.42±1.9 c-k | 6.3±1.12 m |
|  |  |  | **12** | 22.21±1.39 def | 10.57±0.28 a-e | 1.05±0.3 b | 7.04±0.79 b | 6.84±0.44 b | 11.33±0.17 bc | 32.18±2.49 e-j | 15.94±1.67 a-f | 7.36±0.32 ijkl |
|  |  | **10** | **0** | 20.2±0.26 ef | 10.78±0.55abcd | 0.99±0.43 b | 6.75±1.46 b | 6.86±0.4 b | 10.81±0.86 bc | 36.92±9.81 b-f | 18±3.51 ab | 7.03±0.5 klm |
|  |  |  | **12** | 20.99±0.29 ef | 10.79±0.46abcd | 1.09±0.32 b | 7.81±1.3 b | 7.24±0.85 b | 11.15±0.32 bc | 36.64±6.68 b-f | 15.95±3.39 a-f | 7.86±0.65 g-l |
| **2** | **8** | **0** | **0** | 31.53±5.71 ab | 12.06±0.11 ab | 2.83±0.49 b | 8.07±1.05 b | 6.92±0.06 b | 12.02±0.94 bc | 26.9±4.08 i-m | 12.62±0.35 g-k | 10.16±1.04abcd |
|  |  |  | **12** | 32.85±9.5 ab | 11.6±0.85 abcd | 2.84±0.46 b | 7.45±1.62 b | 6.99±0.19 b | 11.16±0.75 bc | 23.81±0.26 klm | 12.59±0.61 g-k | 9.53±1.34 cde |
|  |  | **5** | **0** | 32.58±1.74 ab | 11.1±0.65 abcd | 11.46±15.2a | 7.67±0.84 b | 6.96±0.12 b | 12.84±0.33 bc | 27.08±0.58 i-m | 12.64±0.24 g-k | 9.2±0.75 def |
|  |  |  | **12** | 36.27±10.11 a | 11.13±0.11abcd | 3.17±0.54 b | 7.79±0.26 b | 6.75±0.08 b | 11.81±0.2 bc | 25.37±2.61 jklm | 11.25±0.76 k | 11±0.2 a |
|  |  | **10** | **0** | 31.2±6.87 ab | 11.4±0.69 abcd | 4.03±0.55 b | 7.86±0.05 b | 6.85±0.08 b | 11.38±1.51 bc | 27.29±3.17 i-m | 13.66±2.73 d-k | 9.76±0.25 cde |
|  |  |  | **12** | 30.02±8.34abcd | 11.06±0.87abcd | 3.03±0.62 b | 7.34±0.62 b | 6.99±0.31 b | 12.21±0.97 bc | 29.48±2.78 g-k | 13.5±0.88 e-k | 10.83±0.76 ab |
|  | **12** | **0** | **0** | 30.81±1.12 abc | 11.43±0.49abcd | 3.15±1.11b | 7.85±0.91 b | 7.2±0.2 b | 11.6±0.46 bc | 26.58±1.6 i-m | 14.26±0.79 c-k | 8.3±0.36 fghi |
|  |  |  | **12** | 30.86±5 abc | 11.76±0.25 abc | 2.9±0.55 b | 7.56±0.72 b | 6.84±0.17 b | 12.13±0.8 bc | 23.26±3.48 klm | 13.15±1.53 e-k | 8.2±0.2 fghi |
|  |  | **5** | **0** | 35.5±9.17 a | 11.26±0.2 abcd | 3.53±0.53 b | 6.86±0.35 b | 6.77±0.3 b | 12.25±1.13 bc | 25.22±2.72 jklm | 12.54±0.63 hijk | 8±1 g-k |
|  |  |  | **12** | 30.62±9.06 abc | 11.83±0.25 abc | 3.19±0.36 b | 6.52±0.04 b | 6.69±0.48 b | 11.6±0.26 bc | 23.57±2.98 klm | 13.03±3.69 e-k | 8.13±0.75 ghij |
|  |  | **10** | **0** | 29.94±7.25abcd | 10.75±0.43 a-e | 2.62±0.49 b | 7.29±0.65 b | 6.89±0.25 b | 11.25±1.71 bc | 25.34±5.5 jklm | 12.74±0.15 f-k | 7.86±0.32 g-l |
|  |  |  | **12** | 31.94±10.07 ab | 11.66±0.15 abc | 2.76±0.15 b | 7.39±0.47 b | 6.84±0.23 b | 11.01±1.14 bc | 22.25±2.72 jklm | 14.27±3.53 c-k | 8.83±0.28 efg |
|  | **16** | **0** | **0** | 26.73±0.68bcde | 11.86±0.4 abc | 2.03±0.03 b | 8.1±0.2 b | 6.48±0.37 b | 12.08±0.14 bc | 26.22±1.32 i-m | 12.5±0.62 hijk | 7.83±0.28 g-l |
|  |  |  | **12** | 30.93±2.1 ab | 12.1±0.1 ab | 3.06±0.19 b | 6.92±0.59 b | 7.12±0.16 b | 12.35±0.44 bc | 25.22±1.69 jklm | 13.23±0.45 e-k | 7.2±0.4 i-m |
|  |  | **5** | **0** | 32.75±7.32 ab | 12.17±0.28 a | 3.02±0.53 b | 7.43±0.69 b | 6.95±0.16 b | 12.03±1.12 bc | 27.8±1.84 h-l | 13.2±0.75 e-k | 6.86±0.32 lm |
|  |  |  | **12** | 30.42±0.73 abc | 12.1±0.26 ab | 2.5±0.22 b | 7.66±0.37 b | 6.87±0.03 b | 11.3±1.62 bc | 23.55±4.06 klm | 12.3±0.36 ijk | 7.33±0.73 ijkl |
|  |  | **10** | **0** | 21.53±9.6 ef | 8.93±6.01 e | 2.93±1.41 b | 6.58±0.47 b | 8.66±0.279a | 16.36±7.29 a | 20.52±12.24 m | 11.59±2.92 jk | 7±0.5 klm |
|  |  |  | **12** | 30.16±6.5 abcd | 12.17±0.16 a | 2.71±0.68 b | 7.14±0.89 b | 6.57±0.47 b | 10.8±0.27 bc | 23.36±1.42 klm | 12.51±0.4 hijk | 7.9±0.65 g-k |

**Table S16** (continued).

| **Traetment** | | | | **Chla** | **Chlb** | **Total Chl** | **Car** | **Prolin** | **Suger** | **Phenol** | **Protein** | **Yield** |
| --- | --- | --- | --- | --- | --- | --- | --- | --- | --- | --- | --- | --- |
| **year** | **Irri** | **B** | **Y** |  |  |  |  |  |  |  |  |  |
| **1** | **8** | **0** | **0** | 11±1 g-k | 5.33±0.57h | 16.33±1.52 gh | 12.66±0.57efg | 2.0±0.05 n | 2.8±0.2p | 18.33±1.52b | 117.33±2.51 m | 2129.31±71.71 abcd |
|  |  |  | **12** | 11.23±0.7 g-k | 5.81±0.7 efgh | 17.05±1.41fgh | 11.77±0 f-ij | 2.13±0.21mn | 3.13±0 op | 22.1±0 b | 143.4±3.53 h-m | 1797.84±226 cdef |
|  |  | **5** | **0** | 12±1 efgh | 6.16±0.28defg | 18.16±1.25 ef | 13.23±0.4cdef | 2.41±0.11lmn | 3.2±0.2 o | 23.33±1.15 b | 129.33±1.15klm | 2329.42±580.05 ab |
|  |  |  | **12** | 14±1 abc | 7±1 abc | 21±1 bc | 14.33±0.57 bc | 3.1±0.17 ij | 3.96±0.05mn | 29±1 b | 173.66±5.85 d-i | 2533.31±245.39 a |
|  |  | **10** | **0** | 13.33±0.57bcde | 6.66±0.35 cde | 20±0.7 cd | 13.66±0.57bcde | 3.03±0.15 ij | 3.7±0.26 n | 29±1 b | 166±6.08 f-k | 2277.9±540.9 abc |
|  |  |  | **12** | 15.33±1.52 a | 7.66±1.52 a | 23±0 a | 16.33±1.15 a | 3.66±0.15 gh | 4.81±0.22 ij | 34.33±1.15 b | 211.66±7.63 cd | 2285.17±388.2 abc |
|  | **12** | **0** | **0** | 9±1 mnop | 3.83±0.28iijkl | 12.83±1.04klmn | 10±1 lmn | 2.54±0.17klm | 3.86±0.15 n | 24±1.73 b | 135±4.58 jklm | 1376.76±231.7 hijk |
|  |  |  | **12** | 8.66±1.15 nop | 4.2±0.34 i | 12.86±1.02klmn | 9.33±0.57 nop | 2.85±0.16 jk | 4.2±0.2 lm | 29.33±1.15 b | 161±2.64 g-l | 1352.89±224.3 hijk |
|  |  | **5** | **0** | 10±1 j-n | 4.06±0.11 i | 14.06±1.1 jk | 11±1 ijkl | 3.4±0.17 hi | 4.63±0.15 jk | 28.33±0.57 b | 165.66±7.37 f-l | 1271.01±92.34 hijk |
|  |  |  | **12** | 12.33±0.57defg | 5.33±0.57 h | 17.66±1.15 fg | 12.66±0.57 efg | 3.9±0.1 fg | 5.24±0.05 fg | 37.66±0.57 b | 199±1.73 c-g | 1629.55±467.7 efgh |
|  |  | **10** | **0** | 11.66±1.15 fghi | 5.33±0.57 h | 17±1 fgh | 11.66±0.57 ghij | 4.19±0.08 ef | 5.2±0.2 fgh | 38±1 b | 188.66±8.08 c-g | 1681.08±343.35 efg |
|  |  |  | **12** | 13.66±0.57 bcd | 6.33±0.57cdef | 20±0 cd | 13±1 def | 4.73±0.11 c | 6.34±0.05 d | 44.66±2.3 b | 279±8.54 b | 1767.01±263.94 def |
|  | **16** | **0** | **0** | 5.66±0.57 r | 2.13±0.23 n | 7.8±0.34 pq | 7±1 rst | 3.03±0.05 ij | 4.92±0.06hij | 30.33±1.52 b | 185.33±9.23 c-h | 1627.02±269.7 efgh |
|  |  |  | **12** | 5.33±0.57 r | 3.03±0.05 lm | 8.36±0.55 opq | 6.66±0.57 st | 3.19±0.17 ij | 5.89±0.1 e | 38.33±0.57 b | 206±4 cde | 1441.94±51.08 f-j |
|  |  | **5** | **0** | 6.66±0.57 qr | 2.76±0.2 mn | 9.43±0.66 o | 7.66±0.57 qrs | 4.29±0.01 def | 5.73±0.27 e | 36±1 b | 204±5.29 cdef | 1508.36±134.47 f-j |
|  |  |  | **12** | 9.66±0.57 k-o | 3.89±0.19 ijk | 13.55±0.5 jkl | 8±1 qr | 5.33±0.57 b | 6.93±0.11 b | 44±1 b | 266.66±11.54b | 1501.51±219.25 f-j |
|  |  | **10** | **0** | 8±1 pq | 3.86±0.23 ijk | 11.86±0.8 mn | 8.66±0.57 opq | 5.49±0.15 b | 6.56±0.2 cd | 45.66±1.52 b | 264.66±5.5 b | 1482.31±149.06 f-j |
|  |  |  | **12** | 11.33±0.57 ghij | 5.33±0.57 h | 16.66±1.15 fgh | 10.66±0.57jklm | 6.33±0.57 a | 7.92±0.12 a | 51.33±1.15 b | 355.33±5.03 a | 1550.45±317.65 f-j |
| **2** | **8** | **0** | **0** | 10.66±0.57 g-l | 5.33±0.57 h | 16±0 hi | 13±1 def | 2±0.01 n | 2.81±0.22 p | 18.66±0.57 b | 117.66±2.08m | 1840.45±344 cdef |
|  |  |  | **12** | 11±1 g-k | 5.33±0.57 h | 16.33±1.15 gh | 12.33±0.57 fgh | 2.2±0.17 mn | 3.3±0.17 o | 21.66±1.52 b | 137±7.21 i-m | 1445.65±191.13 f-j |
|  |  | **5** | **0** | 12±1 efgh | 6±1 efgh | 18±1 ef | 13.66±0.57bcde | 2.42±0.15klmn | 3.23±0.2 o | 23.33±0.57 b | 127.33±2.3lm | 1557.73±199.2 fghi |
|  |  |  | **12** | 14.33±0.57 abc | 7±0 abc | 21.33±0.57 bc | 14.66±0.57 b | 3.13±0.15 ij | 3.98±0.03mn | 30.66±1.15 b | 174±9.64 d-i | 1680.24±230.28 efg |
|  |  | **10** | **0** | 13±1 cdef | 6.83±0.28 bcd | 19.83±1.25 cd | 14±1 bcd | 3.21±0.09 ij | 3.78±0.1 n | 28.66±1.52 b | 169±1 e-j | 1694.84±223.6 defg |
|  |  |  | **12** | 14.66±0.57 ab | 7.5±0.5 ab | 22.16±0.76 ab | 16.66±0.57 a | 3.68±0.12 gh | 4.89±0.19 ij | 33.66±1.52 b | 215±5 c | 2020.09±264.8 bcde |
|  | **12** | **0** | **0** | 9.33±0.57 l-p | 3.86±0.23 ijk | 13.2±0.72 jklm | 10.16±1.04klmn | 2.53±0.09 klm | 3.87±0.12 n | 25.33±1.52 b | 136.66±4.93 i-m | 1417.01±70.44 f-j |
|  |  |  | **12** | 8.33±0.57 op | 3.97±0.16 ij | 12.3±0.62 lmn | 9.66±0.57 mno | 2.83±0.04 jkl | 4.38±0.1 kl | 29.33±1.15 b | 163±6.08 g-l | 1307.5±48.56 hijk |
|  |  | **5** | **0** | 10.33±0.57 i-m | 4.33±0.57 i | 14.66±0.57 ij | 11.33±1.15 hijk | 3.46±0.18 hi | 4.67±0.08 ijk | 29±1 b | 166.33±5.5 f-k | 1320.15±26.37 hijk |
|  |  |  | **12** | 12±1 efgh | 5.33±0.57 h | 17.33±1.15 fgh | 12.33±0.57 fgh | 3.93±0.11 fg | 5.27±0.2 f | 37.33±1.52 b | 195±5.56 c-g | 1294.81±372.9 hijk |
|  |  | **10** | **0** | 12±1 efgh | 5.5±0.26 gh | 17.5±0.98 fgh | 12±1 fghi | 4.2±0.08 ef | 5.26±0.25 f | 38.33±1.52 b | 188±2.64 c-g | 1259.65±312.2 hijk |
|  |  |  | **12** | 13±1 cdef | 6.33±0.57 cdef | 19.33±0.57 de | 13±1 def | 4.66±0.25 cd | 6.32±0.11 d | 44.66±1.15 b | 286.66±3.51 b | 1382.98±117 hijk |
|  | **16** | **0** | **0** | 5.33±0.57 r | 2.13±0.23 n | 7.46±0.5 q | 7±1 rst | 3.03±0.05 ij | 4.95±0.07 ghi | 30.33±1.52 b | 189.33±5.03 c-g | 1299.15±52.49 hijk |
|  |  |  | **12** | 5.66±0.57 r | 3.21±0.32jklm | 8.88±0.83 opq | 6.33±0.57 t | 3.39±0.09 hi | 5.88±0.11 e | 38.66±1.15 b | 208±10.58 cd | 1205.01±17.84 ijkl |
|  |  | **5** | **0** | 6.66±1.15 qr | 2.66±0.28 mn | 9.33±1.44 op | 7.66±0.57 qrs | 4.39±0.22 cde | 5.8±0.2 e | 35.66±1.52 b | 202.33±7.5 cdef | 1156.81±197.84 jkl |
|  |  |  | **12** | 9.66±1.15 k-o | 4.11±0.19 i | 13.78±1.35 jkl | 8.33±0.57 pq | 5.33±0.57 b | 6.83±0.28 bc | 45.66±1.15 b | 263.33±5.77 b | 1131.27±251.62 kl |
|  |  | **10** | **0** | 8.33±0.57 po | 3.1±0.17 klm | 11.43±0.75 n | 8.66±0.57 opq | 5.49±0.23 b | 6.56±0.11 cd | 118.33±124.42a | 185±136.92 c-h | 782.93±686.53 l |
|  |  |  | **12** | 10.66±0.57 g-l | 5.66±0.57 fgh | 16.33±1.15 gh | 10.33±1.15 j-n | 6.66±1.15 a | 7.98±0.52 a | 50.33±1.52 b | 366.66±15.27 a | 1157.03±87.34 jkl |

**Table S16** (continued).

| **Traetment** | | | | **Flavonoid** | **MDA** | **SOD** | **APX** | **GPX** | **CAT** | **PPO** | **LL** | **LW** | **NGP** |
| --- | --- | --- | --- | --- | --- | --- | --- | --- | --- | --- | --- | --- | --- |
| **year** | **Irri** | **B** | **y** |  |  |  |  |  |  |  |  |  |  |
| **1** | **8** | **0** | **0** | 10.66±1.15 k | 6.66±0.57 g-n | 0.029±0.002 m | 0.039±0.001 p | 0.035±0.001 k | 0.019±0.001 r | 0.021±0.001 b | 6.86±0.55 d-h | 5±0.46 b | 9.9±0.54 b |
|  |  |  | **12** | 12.6±0.7 ijk | 5.9±1.41 h-n | 0.031±0.002 lm | 0.047±0.001 o | 0.043±0.002 ijk | 0.021±0.0007 pqr | 0.027±0.0007 b | 5.56±0.98 d-i | 3.74±0.62 b | 9.62±0.42 b |
|  |  | **5** | **0** | 11.66±0.57 jk | 6.16±0.28 h-n | 0.033±0.001klm | 0.051±0.001 no | 0.04±0.002 ijk | 0.022±0.0005opqr | 0.027±0.001 b | 6.83±0.29 d-h | 5.02±0.21 b | 9.94±1.66 b |
|  |  |  | **12** | 14.66±0.57 hij | 5.86±0.23 j-n | 0.04±0.002 j | 0.062±0.002 k | 0.054±0.003 fg | 0.027±0.0005 lmn | 0.039±0.002 b | 5.43±0.55 ij | 3.9±0.37 b | 9.85±2.3 b |
|  |  | **10** | **0** | 13.66±0.57 ijk | 5.53±0.5 lmn | 0.037±0.001 jkl | 0.058±0.001 klm | 0.046±0.001 hi | 0.027±0.001 lmn | 0.033±0.002 b | 5.92±0.49ghij | 4.1±0.48 b | 9.17±1.73 b |
|  |  |  | **12** | 17.33±0.57 gh | 4.96±0.05 mn | 0.042±0.001 hij | 0.069±0.001 j | 0.076±0.003 c | 0.038±0.001 def | 0.05±0.002 b | 5.75±1.13 hij | 4.18±0.77 b | 8.86±1.52 b |
|  | **12** | **0** | **0** | 15.33±0.57 hi | 9±1 def | 0.037±0.001 jkl | 0.055±0.004 lmn | 0.045±0.002 ij | 0.24±0.01 a | 0.029±0.001 b | 6.48±0.51 d-i | 4.46±0.65 b | 7.29±1.54 b |
|  |  |  | **12** | 17.66±0.57 gh | 7.93±0.11 efghi | 0.043±0.001 f-j | 0.061±0.001 k | 0.055±0.003 fg | 0.029±0.0005 klm | 0.037±0.002 b | 5.81±0.53ghij | 3.88±0.25 b | 7.93±1.55 b |
|  |  | **5** | **0** | 17.33±0.57 gh | 8.33±0.57 defg | 0.041±0.001 ij | 0.071±0.001 j | 0.054±0.003 fgh | 0.026±0.001mnop | 0.034±0.002 b | 5.82±1.01ghij | 4±0.57 b | 8±0.86 b |
|  |  |  | **12** | 19.33±0.57 fg | 7±1 f-m | 0.047±0.0005 e-i | 0.09±0.003 cd | 0.073±0.004 c | 0.033±0.001 ghij | 0.05±0.001 b | 5.56±1.35 hij | 3.6±1.04 b | 8.28±1.88 b |
|  |  | **10** | **0** | 19.33±0.57 fg | 6.9±0.17 g-m | 0.047±0.001 efgh | 0.078±0.002 ghi | 0.06±0.002 ef | 0.032±0.001 hijk | 0.041±0.001 b | 6.1±0.31 e-j | 4.22±0.22 b | 9.11±0.2 b |
|  |  |  | **12** | 21.66±0.57 ef | 5.63±0.32 klmn | 0.056±0.002 bcd | 0.126±0.005 b | 0.097±0.002 b | 0.041±0.001 cd | 0.065±0.004 b | 5.89±0.2 ghij | 3.94±0.19 b | 9.56±2.12 b |
|  | **16** | **0** | **0** | 22.66±1.52 de | 13.33±0.57 a | 0.048±0.001 efg | 0.081±0.002 fgh | 0.059±0.001 ef | 0.029±0.001 klm | 0.039±0.001 b | 6±0.24 ghij | 4.14±0.38 b | 8.29±3.22 b |
|  |  |  | **12** | 26±1 c | 12±1 ab | 0.052±0.002 de | 0.09±0.003 cd | 0.071±0.002 cd | 0.036±0.001 efgh | 0.052±0.001 b | 5.82±0.27ghij | 4.1±0.29 b | 7.98±2.13 b |
|  |  | **5** | **0** | 25.33±1.52 cd | 12±1 ab | 0.052±0.0005 de | 0.087±0.002 de | 0.07±0.003 cd | 0.031±0.001 ijkl | 0.043±0.002 b | 6.33±1 d-j | 4.46±0.61 b | 11.32±1.64 b |
|  |  |  | **12** | 30±1 b | 10.33±0.57 bcd | 0.061±0.001 b | 0.129±0.001 b | 0.096±0.002 b | 0.039±0.001 de | 0.063±0.002 b | 5.48±0.73 ij | 3.95±0.15 b | 8.53±0.89 b |
|  |  | **10** | **0** | 28±1 bc | 10.33±0.57 bcd | 0.058±0.002 bc | 0.094±0.004 c | 0.077±0.002 c | 0.037±0.0005defg | 0.051±0.001 b | 6.47±0.59 d-i | 4.64±0.43 b | 7.95±1.27 b |
|  |  |  | **12** | 35.33±0.57 a | 8±1 efgh | 0.07±0.002 a | 0.171±0.003 a | 0.15±0.01 a | 0.055±0.001 b | 0.078±0.003 b | 6.05±0.89 f-j | 4.04±0.39 b | 8.31±1.3 b |
| **2** | **8** | **0** | **0** | 10.66±0.57 k | 6.66±0.57 g-n | 0.029±0.002 m | 0.041±0.001 p | 0.037±0.002 jk | 0.19±0.001 r | 0.021±0.001 b | 8±1.32 abc | 6.23±0.3 b | 9.58±1.61 b |
|  |  |  | **12** | 13±1 ijk | 5.9±0.17 i-n | 0.032±0.001 lm | 0.047±0.002 o | 0.043±0.002 ijk | 0.021±0.0005 qr | 0.029±0.002 b | 7.26±0.4 a-e | 4.83±0.15 b | 8.05±0.59 b |
|  |  | **5** | **0** | 11.33±0.57 k | 5.83±0.28 j-n | 0.033±0.003 klm | 0.054±0..04 mn | 0.041±0.002 ijk | 0.022±0.0005opqr | 0.26±0.001 b | 8.26±0.2 a | 5.76±0.41 b | 9.01±0.24 b |
|  |  |  | **12** | 14.66±1.15 hij | 5.16±0.28 mn | 0.041±0.001 hij | 0.062±0.001 k | 0.057±0.005 ef | 0.027±0.002 lmn | 0.04±0.001 b | 5.96±0.25ghij | 4.13±0.25 b | 9.03±0.83 b |
|  |  | **10** | **0** | 13.66±1.15 ijk | 5.33±0.57 lmn | 0.039±0.001 jk | 0.06±0.003 kl | 0.047±0.002 ghi | 0.027±0.001 lmn | 0.035±0.005 b | 6.93±0.37 b-g | 4.78±0.51 b | 8.61±1.55 b |
|  |  |  | **12** | 17.66±0.57 gh | 4.76±0.25 n | 0.042±0.002 ghij | 0.072±0.001 j | 0.078±0.002 c | 0.039±0.0005 de | 0.051±0.002 b | 6.56±0.92 hij | 4.6±0.95 b | 9.21±0.97 b |
|  | **12** | **0** | **0** | 16±1 hi | 9.66±1.15 cde | 0.037±0.001 jkl | 0.058±0.001 klm | 0.045±0.002 ij | 0.024±0.001 nopq | 0.028±0.002 b | 7.5±0.86 abcd | 5.53±0.45 b | 8.01±0.89 b |
|  |  |  | **12** | 17.66±1.15 gh | 7.73±0.23 e-j | 0.042 ghij | 0.063±0.002 k | 0.056±0.002 f | 0.028±0.001 klm | 0.04±0.002 b | 6.5±0.69 d-i | 4.43±0.58 b | 8.18±1.27 b |
|  |  | **5** | **0** | 17.66±0.57 gh | 7.66±0.57 e-k | 0.041±0.001 ij | 0.073±0.003 ij | 0.055±0.002 fg | 0.026±0.0005mno | 0.035±0.001 b | 7.2±0.52 a-f | 4.9±0.36 b | 8.73±0.82 b |
|  |  |  | **12** | 20±1 efg | 6.66±0.57 g-n | 0.048±0.002 efg | 0.09±0.002 cd | 0.077±0.002 c | 0.034±0.002 fghi | 0.052±0.002 b | 5.9±0.17 ghij | 3.73±0.49 b | 8.15±0.27 b |
|  |  | **10** | **0** | 20±1 efg | 6.33±0.57 g-n | 0.049±0.002 ef | 0.078±0.002 hi | 0.061±0.002 ef | 0.032±0.001 hijk | 0.042±0.002 b | 6.9±0.34 b-h | 5.16±0.57 b | 8.35±1.85 b |
|  |  |  | **12** | 22±1 ef | 5.16±0.28 mn | 0.059±0.001 b | 0.127±0.002 b | 0.097±0.002 b | 0.041±0.001 cd | 0.066±0.003 b | 5.8±0.3 ghij | 3.53±0.45 b | 7.53±1.45 b |
|  | **16** | **0** | **0** | 22.66±1.15 de | 13±1 a | 0.049±0.001 ef | 0.083±0.002 efg | 0.06±0.002 ef | 0.029±0.001 jklm | 0.04±0.002 b | 7.26±0.46 a-e | 4.8±0.6 b | 8.86±0.7 b |
|  |  |  | **12** | 27±1 bc | 11.66±0.57 abc | 0.052±0.001 cde | 0.089±0.002 cd | 0.074±0.002 c | 0.037±0.001 efg | 0.052±0.001 b | 6.36±0.45 d-j | 3.86±0.23 b | 8.45±0.42 b |
|  |  | **5** | **0** | 25.33±1.52 cd | 12±1 ab | 0.053±0.001 cde | 0.085±0.003 def | 0.07±0.002 cd | 0.031±0.001 ijk | 0.043±0.004 b | 8.06±0.3 ab | 5.26±0.46 b | 8.55±1.7 b |
|  |  |  | **12** | 29.66±1.52 b | 10.33±0.57 bcd | 0.06±0.001 b | 0.012±0.0002 q | 0.095±0.003 b | 0.039±0.002 de | 0.063±0.002 b | 5.2±0.36 j | 3.13±0.55 b | 8.33±1.44 b |
|  |  | **10** | **0** | 22.3±10.78def | 7.35±6.31 f-l | 0.07±0.02 a | 0.089±.01 cd | 0.065±0.02 de | 0.044±0.008 c | 2.03±3.4 a | 5.8±1.63 ghij | 14.8±17.8 a | 413.5±701 a |
|  |  |  | **12** | 36±1 a | 7.66±0.57 e-k | 0.07±0.002 a | 0.174±0.005 a | 0.151±0.002 a | 0.056±0.002 b | 0.077±0.004 b | 5.53±0.64 ij | 3.33±0.8 b | 7.81±0.46 b |

**Irri:** Different levels of water deficit**, B:** 24-epibrassinolide**, Y:** yeast extract, **PH:** Plant height, **NN:** Number of nodes, **NL:** Distance internode, **SD:** Stem diameter, **PD:** PPO diameter, **PL:** PPO length, **PW 20:** Weight 20 PPOs, **GW 100:** Weight 100 grains, **NP:** Number of PPOs , **LL:** Leaf length , **Yi:** Yield, **LW:** Leaf width, **NGP:** Number of seeds in PPO, **Chla:** Chlorophyll a, **Chlb:** Chlorophyll b, **ChlT:** Total chlorophyll, **Car:** Carotenoid, , **MDA**: Malondialdehyde, **CAT:** Catalase, **SOD:** Superoxide dismutase, **GPX:** Guaiacol peroxidase, **APX:** Ascorbate peroxidase, **PPO:** Polyphenol oxidase.

* and ** Significantly at the probability level of %5 and %1, respectively. Mean comparison was performed by LSD method at 5% probability. Columns with similar letters did not differ significantly.
